# Supplementary material for: Neighbourhood Socioeconomic Processes and Dynamics and Healthy Ageing: A Scoping Review
Source: Int J Environ Res Public Health. 2022 May 31;19(11):6745. doi: 10.3390/ijerph19116745 (PMC9180257; doi:10.3390/ijerph19116745)
Supplement: Supplementary file 1 [file ijerph-19-06745-s001.zip › Supplementary material - II - KEYWORDS.pdf]

Table S2: Terms used in the search expression of Scopus, Web of Science and Pubmed

KEYWORDS		
OLDER ADULTS	NEIGHBOURHOOD	NEIGHBOURHOOD SOCIOECONOMIC PROCESSES AND DYNAMICS
"Ageing" OR "Aging" OR "Older adult*" OR "Older person*" OR "Older people" OR "Elder*" OR "Old age" OR "Senior*" OR "Late life" OR "Later life"	"Neighborhood*" OR "Neighbourhood*" OR "Spatial" OR "Zip code*" OR "Census tract*"	"Deprivation" OR "Disadvantage*" OR "Segregation" OR "Polarization" OR "Socioeconomic" OR "Deprived" OR "Unemployment" OR "Gentrification" OR "Urban renewal" OR "Poverty" OR "Income"

EXACT EXPRESSION USED IN EACH DATABASE

SCOPUS EXPRESSION:

<u>TITLE, ABSTRACT, KEYWORD</u>

TITLE-ABS-KEY ("Ageing" OR "Aging" OR "Older adult*" OR "Older person*" OR "Older people" OR "Elder*" OR "Old age" OR "Senior*" OR "Late life" OR "Later life") AND TITLE-ABS-KEY ("Neighborhood*" OR "Neighbourhood*" OR "Spatial" OR "Zip code*" OR "Census tract*") AND TITLE-ABS-KEY ("Deprivation" OR "Disadvantage*" OR "Segregation" OR "Polarization" OR "Socioeconomic" OR "Deprived" OR "Unemployment" OR "Gentrification" OR "Urban renewal" OR "Poverty" OR "Income")

RESULTS: 3874

Web of Science EXPRESSION:

<u>TITLE, ABSTRACT, KEYWORDS</u>

#1

TI=(("Ageing" OR "Aging" OR "Older adult*" OR "Older person*" OR "Older people" OR "Elder *" OR "Old age" OR "Senior*" OR "Late life" OR "Later life") AND ("Neighborhood*" OR "Neighbourhood*" OR "Spatial" OR "Zip code*" OR "Census tract*") AND ("Deprivation" OR "Disadvantage*" OR "Segregation" OR "Polarization" OR "Socioeconomic" OR "Deprived" OR "Unemployment" OR "Gentrification" OR "Urban renewal" OR "Poverty" OR "Income"))

#2

AB=(("Ageing" OR "Aging" OR "Older adult*" OR "Older person*" OR "Older people" OR "Elde r*" OR "Old age" OR "Senior*" OR "Late life" OR "Later life") AND ("Neighborhood*" OR "Neighbourhood*" OR "Spatial" OR "Zip code*" OR "Census tract*") AND ("Deprivation" OR "Disadvantage*" OR "Segregation" OR "Polarization" OR "Socioeconomic" OR "Deprived" OR "Unemployment" OR "Gentrification" OR "Urban renewal" OR "Poverty" OR "Income"))

#3

AK=(("Ageing" OR "Aging" OR "Older adult*" OR "Older person*" OR "Older people" OR "Elde r*" OR "Old age" OR "Senior*" OR "Late life" OR "Later life") AND ("Neighborhood*" OR "Neighbourhood*" OR "Spatial" OR "Zip code*" OR "Census tract*") AND ("Deprivation" OR

"Disadvantage*" OR "Segregation" OR "Polarization" OR "Socioeconomic" OR "Deprived" OR "Unemployment" OR "Gentrification" OR "Urban renewal" OR "Poverty" OR "Income"))

RESULTS #1 OR #2 OR #3: 1815

PUBMED EXPRESSION:

<u>TITLE/ABSTRACT:</u>

(("Ageing"[Title/Abstract] OR "Aging"[Title/Abstract] OR "Older adult*"[Title/Abstract] OR "Older person*"[Title/Abstract] OR "Older people"[Title/Abstract] OR "Elder*"[Title/Abstract] OR "Old age"[Title/Abstract] OR "Senior*"[Title/Abstract] OR "Late life"[Title/Abstract] OR "Later life"[Title/Abstract]) AND ("Neighborhood*"[Title/Abstract] OR "Neighbourhood*"[Title/Abstract] OR "Spatial"[Title/Abstract] OR "Zip code*"[Title/Abstract] OR "Census tract*"[Title/Abstract])) AND ("Deprivation"[Title/Abstract] OR "Disadvantage*"[Title/Abstract] OR "Segregation"[Title/Abstract] OR "Polarization"[Title/Abstract] OR "Socioeconomic"[Title/Abstract] OR "Deprived"[Title/Abstract] OR "Unemployment"[Title/Abstract] OR "Gentrification"[Title/Abstract] OR "Urban renewal"[Title/Abstract] OR "Poverty"[Title/Abstract] OR "Income"[Title/Abstract])

RESULTS: 1411
