# Supplementary material for: Neighbourhood Socioeconomic Processes and Dynamics and Healthy Ageing: A Scoping Review
Source: Int J Environ Res Public Health. 2022 May 31;19(11):6745. doi: 10.3390/ijerph19116745 (PMC9180257; doi:10.3390/ijerph19116745)
Supplement: Supplementary file 1 [file ijerph-19-06745-s001.zip › Supplementary material - III - Table studies characteristics.pdf]

Table S3: Characteristics of the included studies (n=122)

Author, years [Reference]	Country	Study Design	Age	Sample size	Exposure	Exposure measurement	Healthy Ageing Outcome	Healthy Ageing Measurement	Association
Alaazi, D. A. et al., 2021 [1]	Ghana	Cross-sectional study	≥60	603	Neighbourhood socioeconomic status/ Neighbourhood deprivation	Two neighbourhoods in Accra, Ghana, were selected based on being classified as a slum - Nima - (densely populated and marked by poverty, congestion, environmental decay, and governmental neglect) or as a non-slum neighbourhood - Adabraka-Asylum Down (largely middle-income neighbourhoo	Quality of life	WHO quality of life assessment tool (WHOQoL-BREF) across four domains: physical, psychological, social, and environment	Multivariable linear regression analyses of the data revealed no statistically significant difference between the slum and non-slum respondents in physical (coeff: 0.5; 95% CI: -1.7, 2.8; p = 0.642) and psychological (coeff: -0.2; 95% CI: -3.0, 2.6; p = 0.893) quality of life (QoL). However, the slum respondents reported significantly higher social QoL than the non-slum respondents (coeff: -3.2; 95% CI: -5.6, -0.8; p = 0.010), while the reverse was true in environmental QoL (coeff: 4.2; 95% CI: 2.3, 6.2; p < 0.001).

						d with well-served municipal services and about 70% of households with solid waste disposal systems and piped water on-premises)			
Almeida, O. P. et al., 2012 [2]	Australia	Longitudinal study	≥60	21 417	Neighbourhood socioeconomic status/ Neighbourhood deprivation	Assessed by the Index of Relative Socio-Economic Disadvantage (IRSED) using geographical postcode area which includes: the proportion of occupied dwellings with connection to the internet, the proportion of employed	Depression	Assessed by the Primary Health Questionnaire (PHQ-9)	For the most disadvantaged quintile, the adjusted odds of clinical depression and major depression were OR=1.36, 95%CI=1.12–1.65 and OR=1.84, 95%CI=1.35–2.52, respectively. Between the baseline and 24-month assessment, the odds of persisting major depression increased progressively with relative socioeconomic disadvantage, reaching a maximum of 2.43,

						people classified as 'labourers', the proportion of people aged 15 years and over with no post-school qualifications, the proportion of people with stated annual household equivalised income between $13,000 and $20,799, the proportion of households renting from government or community organisations, the proportion of people in the labour force that is			95%CI=1.32–4.48 for participants in the most compared with the least disadvantaged quintile group.

						unemployed, the proportion of one parent families with dependent offspring, the proportion of households paying rent less than $120 per week, the proportion of people under 70 years who have a long-term health condition or disability and need assistance, the proportion of occupied dwellings with no car, the proportion of people who identify themselves as			

						Aboriginal, the proportion of occupied dwellings requiring one or more extra bedrooms, the proportion of people aged 15 years or over who are separated or divorced, the proportion of people classified as machinery operators and drivers, the proportion of people aged 15 years and over who did not go to school, the proportion of people classified as low skill			

						community and personal service workers, and the proportion of people who do not speak English well. The IRSED was divided into quintiles			
Amuzu A., et al., 2009 [3]	United Kingdo m	Longitudinal study	≥60	3522	Neighbourhoo d socioeconomic status/ Neighbourhoo d deprivation	Super output area was used to assign scores for area deprivation to each woman, based on residential postcode using ten indicators: manual social class, no bathroom in the house, no hot water in the house, shared	Health behaviours	Diet was assessed using a Food Frequency Questionn aire summariz ed by frequency of consumpti on. Smoking habits (current and past)	Elderly women living in higher deprived areas have higher odds of eating fewer fruits and vegetables (OR = 2.10; 95% CI: 1.56-2.82) as well as chicken and fish (OR = 1.42; 95% CI: 1.04-1.94), higher odds of smoking (OR = 2.27; 95% CI: 1.72-3.01), and higher odds of lack of regular physical exercise (OR = 1.70; 95% CI: 1.27-2.27) compared to the ones living in more affluent areas

						bedroom, no access to a car, left full-time education early, manual social class, local authority housing, no car access, state pension only		were asked. Physical activity was assessed by asking participants their habitual physical activity in hours per week and to rate their own walking speeds	
Aneshensel, C. S. et al., 2007 [4]	United States of America	Cross-sectional study	≥70	3 442	Neighbourhood socioeconomic status/ Neighbourhood deprivation	Neighbourhood-level socioeconomic status was assessed with two measures using data from the 1990 Census at the tract level	Depressive symptoms	Measured with eight items from the longer Center for Epidemiologic Studies–Depression scale	Symptoms tended to be high in tracts that were socioeconomically disadvantaged (β=0.258; standard error (SE)=0.040; p<0.001), had few affluent residents (β=-0.917; SE=0.209; p<0.001), and had large communities of color

						(n=1217). The first one included the proportion of residents aged 25 or older without a high school degree, households receiving public assistance income, residents living below the poverty level, and residents aged 16 or older who were unemployed. The second one assessed the proportion of households with incomes of $50,000 or more			(African American: β=0.468; SE=0.122; p<0.001; Hispanic: β=1.123; SE=0.226; p<0.001). Symptoms were also high in neighbourhoods with a high proportion of long-term residents (β=0.830; SE=0.296; p<0.01). The relative presence of older residents was not significantly associated with symptoms. However, most of this variation was accounted for by the characteristics of residents

Annear, M. J. et al., 2009 [5]	New Zealand	Mixed-methods study	65-91	63	Neighbourhood socioeconomic status/ Neighbourhood deprivation	Measured by the New Zealand Deprivation Index constructed from indicators of socioeconomic status taken from the national census. The neighbourhoods in urban Christchurch were selected based on their contrasting positions on the New Zealand Deprivation Index - the grouping of high-deprivation suburbs to the	Physical activity	Leisure-time physical activity (LTPA) was assessed by a recall survey that indicated the number of times during the previous two weeks that the participant engaged in LTPAs. Participants also did a semi-structured interview to identify how the	Neighbourhood deprivation affected the elderly's LTPA (β=0.346, p=0.004). Participants identified health problems, lack of interest or motivation, no activity partner and traffic as barriers to participate in LTPAs. In addition, high-deprived neighbourhood participants identified the lack of appropriate facilities and resources and the unattractive environment and crime and antisocial behaviour, contributing to their lack of participation in LTPAs. Contrarily, low-deprived neighbourhoods identified that their LTPA was supported by having a well-served leisure environment, attractive and walkable

						east of the city centre, referred to as "East-town", and the cluster of low-deprivation suburbs to the west, referred to as "West-town". After determining these areas, a 1000-metre radius was selected for the neighbourhood boundary		same group of older adults from high- and low-deprivation Neighbourhoods perceived their local environment and identify factors that older adults perceived to influence their LTPA participation.	surroundings, and socially responsible residents.
Annear, M. J. et al., 2009 [6]	New Zealand	Mixed-methods study	Mean age of high-	63	Neighbourhood socioeconomic	Measured by the New Zealand	Physical activity	Q methodology to	Low-deprived neighbourhoods had preferences for leisure

			and low-deprived neighbourhoods: 76 years and 77 years, respectively		status/ Neighbourhood deprivation	Deprivation Index constructed from indicators of socioeconomic status taken from the national census. The neighbourhoods in urban Christchurch were selected based on their contrasting positions on the New Zealand Deprivation Index - the grouping of high-deprivation suburbs to the east of the city centre, referred to as		identify neighbourhood leisure settings and also a semi-structured interview identified how older adults from high- and low-deprivation neighbourhoods perceived their local environment and identified factors that older adults perceived to	settings with aesthetic activities, walkable nature and heritage walk and perceived that their neighbourhood facilitated active leisure participation. Conversely, high-deprived neighbourhoods had preferences for leisure settings with a restful nature, functional facilities, and social interaction and generally perceived their neighbourhood as a constraint to active leisure participation

						"East-town", and the cluster of low-deprivation suburbs to the west, referred to as "West-town". After determining these areas, a 1000-metre radius was selected for the neighbourhood boundary		influence their active leisure participation	
Araújo, C. A. H. et al., 2018 [7]	Brazil	Cross-sectional study	≥60	1 197	Neighbourhood socioeconomic status/ Neighbourhood deprivation	Assessed by the contextual income (mean monthly nominal income of heads of permanent private households), population density, percentage of	Obesity	Abdominal obesity was defined as waist circumference (WC) according to World Health Organization (WHO) guidelines	After adjusted, women with an intermediate income (OR=0.62, 95% IC: 0.41-0.94) or higher paved streets (OR=0.66, 95% IC: 0.44-0.99) had lower odds of abdominal obesity while, in men, there was not a significant association. Women with intermediate income (OR=0.52, 95% IC: 0.33-

						public lighting, paved streets, sidewalks, and commerce in the census tract, street density and connectivity, mixed land use (calculated as the presence or absence of five types of land use: residential, commercial, recreational green areas, institutional, and others) and recreational green areas at the census tract level		(obesity: men=102cm and women=88cm), and overall obesity was defined according to the WHO cutoff points (obesity: BMI > 30kg/m2)	0.82) and men living in an area with high street connectivity (OR=0.43, 95% IC: 0.20-0.94) and intermediate percentage of local commerce (OR=0.46, 95% IC: 0.23-0.95) had lower odds of overall obesity.

Auchincloss, A. H. et al., 2001 [8]	United States of America	Cross-sectional study	≥65	12 341	Neighbourhood socioeconomic status/ Neighbourhood deprivation	Neighbourhood characteristics were assessed through block-group geographic identifiers including: population density and geographic distribution, primary health care professional shortages, the proportion of residents with poverty incomes, proportion of residents sharing a common ancestry, and proportion of	Access to healthcare	Respondents who met any of the following criteria were categorized as having problems accessing health care: The respondent had no particular person or place that he or she usually goes when he or she is sick or needs health advice. During	Considering personal, structural and neighbourhood characteristics, poorer neighbourhoods have higher odds of health access problems (OR=1.05; 95% IC: 1.01-1.10). Living in relatively heterogeneous neighbourhoods had higher odds of having health access problems compared with those living in homogeneous neighbourhoods (OR=1.34; 95% IC: 1.11-1.63)

						elderly residents		the past 12 months, the respondent delayed seeking medical care because of worry about the cost. During the past 12 months, the respondent needed care but did not get at least one of the following: (a) medical or surgical care, (b) dental	

								care, (c) mental health care, (d) prescription medication, or (e) eyeglasses	
Balamurugan, A. et al., 2013 [9]	United States of America	Longitudinal study	≥35 (Results only consulted for ≥60)	8 930	Neighbourhood socioeconomic status/ Neighbourhood deprivation	Census block groups (n=2136) were used as proxies for neighbourhoods. Block groups were ranked by racial/ethnicity, education, poverty, population density, and mobility. The Principal Component Analysis method	Stroke mortality	Death certificates from 2005 to 2009 for Arkansas that coded stroke as an underlying cause of death with International Classification of Diseases, Tenth Revision codes I60	Together, the five-block groups' covariates explain 6.1% of the deviance in stroke mortality among block groups at age 65 to 74

						combined neighbourhoods (census block groups) with similar profiles based on these five covariates		to I69. The residential address of each case was geocoded to a latitude/longitude	
Basta, N. E., et al., 2008 [10]	United Kingdom	Cross-sectional study	≥65	13004	Neighbourhood socioeconomic status/ Neighbourhood deprivation	Assessed using the Townsend deprivation score with considers the proportion of unemployed individuals aged 16–59/64, the proportion of households without a car, with more than one person per room and that are not owner-occupied, at the	Cognitive and functional impairment	Cognitive functioning was determined by the Mini-Mental State Examination and physical functioning was determined by the activities of daily living self-	Elderly living in more deprived areas have higher odds of cognitive (OR = 2.3; 95% CI: 1.8-3.0) and functional impairment (OR = 1.6; 95% CI: 1.4-1.9) than elderly living in the least deprived areas

						enumeration district		reported scale	
Beard, J. R. et al., 2009 [11]	United States of America	Cross-sectional study	≥65	Undisclosed (ca. 150,000)	Neighbourhood socioeconomic status/ Neighbourhood deprivation	Data from the 2000 U.S. Census was used to capture the population composition in each census tract (n=2138). In addition, a range of secondary administrative data sources, including the NYC Housing and Vacancy Survey and the fiscal 2002 NYC Mayor's Management Report, describe the physical environment and a survey	Disability	Disability data from the long-form (SF3) of the 2000 U.S. Census, which is collected from approximately one in six households	High neighbourhood socioeconomic status (β=-5.6, 95% CI: -6.1, -5.1), residential stability (β=-1.9, 95% CI: -2.4, -1.4), living in areas with high proportions of foreign born and low proportion of African American (β=-3.3, 95% CI: -3.9, -2.7), low crime levels (β=-0.9, 95% CI: -1.5, -0.2), and good street characteristics (β=-0.7, 95% CI: -1.2, -0.2) were significantly associated with low prevalence of physical disability. In addition, high neighbourhood socioeconomic status (β=-4.6, 95% CI: -5.2, -4.0), residential stability (β=-0.8, 95% CI: -1.3, -0.3), and and good street characteristics (β=-0.8,

						of 4,000 NYC residents to capture social characteristics			95% CI: -1.3, -0.3) were significantly associated with low prevalence of going-outside-the-home disability
Beere, P. et al., 2019 [12]	New Zealand	Cross-sectional study	≥65	52 973	Neighbourhood socioeconomic status/ Neighbourhood deprivation	Measured with the New Zealand Deprivation Index (NZDep13) using the domicile code	Loneliness	Self-reported loneliness was assessed through the question: “Says or indicates that he/she feels lonely.”	Socioeconomic deprivation positively correlated with loneliness (OR=1.002)
Behanova, M. et al., 2015 [13]	Slovakia and Netherlands	Cross-sectional study	≥65	Slovak sample: 644 and Dutch sample: 760	Neighbourhood socioeconomic status/ Neighbourhood deprivation	Area deprivation was measured in Slovakia with the Census data for neighbourhood-level	Health-risk behaviours	Five indicators: daily smoking ("Do you currently smoke?"), binge drinking	When adjusting for age, sex, interactions and ethnicity - Netherlands: The least favourable neighbourhood was more likely to smoke daily (OR:2.32, 95% IC: 1.25-4.30) and be overweight (OR=1.84,

						unemployment and in the Netherlands with registered unemployment municipality data for Dutch neighbourhoods		(How often do you drink six portions or more of alcohol at once"?), physical activity (In your leisure time, how often do you do physical exercise for at least 30 min which makes you at least mildly short of breath or perspire?") , consumpti	95% CI: 1.24-2.75). Slovakia: The least favourable neighbourhood were less likely to smoke daily (OR:0.30, 95% IC: 0.10-0.88). When adjusting for the individual-level socio-economic position - Netherlands: The least favourable neighbourhood were more likely to be overweight (OR:1.83, 95% IC: 1.14-2.95), to be overweight (OR=1.84, 95% CI: 1.24-2.75). Slovakia: The least favourable neighbourhood were less likely to be overweight (OR:0.55, 95% IC: 0.31-0.98)

								on of fruits and vegetables ("On average, how many portions of fruit/vegetables do you eat a day?" and BMI (calculated from the self-reported height and weight)	
Behanova, M. et al., 2017 [14]	Slovakia and Netherlands	Cross-sectional study	≥65	Slovak sample: 638 and Dutch sample: 750	Neighbourhood socioeconomic status/ Neighbourhood deprivation	Area deprivation was measured in Slovakia with the Census data for neighbourhood-level	Mental health	Measured by the General Health Questionnaire (GHQ12) tool to detect	Neighbourhood deprivation was not associated with the mental health of elderly in Slovakia and the Netherlands

						unemployment and in the Netherlands with registered unemployment municipality data for Dutch neighbourhoods		mental disorders in the general population, where having mental health problems was defined as a GHQ-total score ≥2	
Bhardwaj, R. et al., 2020 [15]	United States of America	Longitudinal study	≥75	144 665	Neighbourhood socioeconomic status/ Neighbourhood deprivation	Area deprivation index was used based on 17 census variables representing the social and economic characteristics of neighbourhoods in the	Years of potential life lost	Calculated by subtracting the age at the time of death from a predetermined endpoint age (100 years old)	Area-level deprivation was negatively associated with the likelihood of becoming a centenarian (Hazard ratio=0.96, 95%CI: 0.94-0.98), p < 0,001)

						United States at the block group level including education, employment, income and poverty, and housing			
Bolstad, C. J. et al., 2020 [16]	United States of America	Cross-sectional study	≥65	998	Neighbourhood socioeconomic status/ Neighbourhood deprivation	The neighbourhood disadvantage index was used to measure neighbourhood disadvantage using the prevalence rates of poverty and female-headed households in the census tract	Depression	The sort form (15-item questionnaire) of the Geriatric Depression Scale was used to capture depressive symptoms	Mid (OR=1.75, 95% CI: 1.04-2.94) and high disadvantaged neighbourhoods (OR=1.71, 95% CI: 1.09-2.68) had greater odds of endorsing significant depressive symptoms than participants in low disadvantaged neighbourhoods, but this association was not significantly related to having a verified depression diagnosis

Bowling, A. & Stafford, M., 2007 [17]	United Kingdom	Cross-sectional study	≥65	761	Neighbourhood socioeconomic status/ Neighbourhood deprivation	Measured by the affluence of the area by using ACORN (A Classification of Residential Neighbourhoods) including age, marital status, ethnic group, reported long-term illness, household size, household tenure, unemployment, occupation and car ownership based on participants' postcodes	Social and physical functioning	Social functioning: social activity and social contacts in the past month and physical functioning was measured by a scale of level of physical difficulty with everyday activities and instrumental Activities of Daily Living	Low neighbourliness had 2.92 (s.e. 1.13) fewer social contacts each month than those reporting high neighbourliness. The affluence of the area and perceived neighbourhood were associated with physical functioning, although these associations disappeared once the adjustment was made for individuals' characteristics
Breeze, E. et al., 2005	Brazil	Cross-sectional study	≥75	5 581	Neighbourhood	The Carstairs Deprivation	Quality of life	Measured in	The relative risk of a poor outcome if doubly

[18]					socioeconomic status/ Neighbourhood deprivation	Index was used based on the prevalence of unemployment, low social class, no car, and overcrowding using Enumeration District census data		interviews based on a 17-item measure of the UK version of the Sickness Impact Profile and the Philadelphia Geriatric Morale Scale	disadvantaged (social class IV/V and most deprived quartile of deprivation) was 1.62 (95% IC:1.2-2.2) for home management, 1.38 (95% IC:1.1-1.8) for mobility, and 1.63 (95% IC:1.1-2.4) for social interaction when compared with social class I/II and least deprived quartile of deprivation
Brenner, A. B. & Clarke, P. J., 2019 [19]	United Kingdom	Cross-sectional study	≥65	5 504	Neighbourhood socioeconomic status/ Neighbourhood deprivation	The local neighbourhood environment was assessed by physical and social aspects of the neighbourhood reported by either interviewer observation or	Shopping difficulty	Assessed as a 5-level measure of shopping difficulty based on participants' past month	Men and women living in neighbourhoods with more socioeconomic disadvantage had a 4–6% greater risk of not shopping independently due to health (vs no difficulty) after accounting for home and local environment characteristics. Neighbourhood social

						the participant. The macro socioeconomic environment was evaluated using the 2010 US Census at the census tract-level (female-headed households with children, unemployed households, households living at or below 150% of the Federal Poverty Level, and households with public assistance income)		reported shopping	cohesion remained significantly associated with a reduced risk of having some versus no difficulty shopping independently for men and women in the fully adjusted model (RR = 0.26, 95% CI (0.12, 0.59) for men; RR = 0.67, 95% CI (0.47, 0.95) for women)
Brown, A. F., et al., 2011	United States of America	Longitudinal study	≥65	4619	Neighbourhood socioeconomic	Based on the residential census tract	Ischemic stroke	It was classified as a first	White elderly living in the most deprived neighbourhoods had a

[20]					status/ Neighbourhood deprivation	defined in the 1990 US decennial Census. The neighborhood socioeconomic status index was constructed by summing the Z-scores of 6 census-derived SES indicators that represent the area's physical and social resources: (1) median household income; (2) median value of housing units; (3) percent households with interest, dividend, or		ischemic stroke adjudicated by a cerebrovascular disease end point committee that also classified stroke subtype and determined whether death was caused by stroke	higher risk of stroke compared to the ones living in more affluent neighbourhoods (Hazard ratio = 1.32; 95% CI: 1.01-1.72), when adjusting for age, sex, income and education. However, when also adjusting for behavioural and biological risk factors the association was no longer significant (Hazard ratio: 1.15; 95% CI: 0.88-1.51). Among African American elderly it was not found a significant association

						rental income; (4) percent of residents ≥25 with a high school degree; (5) percent of residents ≥25 with a college degree; and (6) percent of residents in executive, managerial, or professional specialty occupations			
Brown, S. C. et al., 2018 [21]	United States of America	Cross-sectional study	≥65	249 405	Neighbourhood socioeconomic status/ Neighbourhood deprivation	Measured using the 2011 U.S. Census Bureau data on the neighbourhood median household income at the census block group level	Mental health	Assessed using the U.S. Centers for Medicare and Medicaid Services' (CMS') Master Beneficiar	Each increase of 0.1 in Normalized Difference Vegetation Index (measures neighbourhood greenness) was associated with lower odds of Alzheimer's disease for residents of low- (OR = 0.87; 95% CI: 0.82-0.93) medium- (OR = 0.91; 95% CI: 0.86-0.95),

								y Summary File using algorithms for diagnoses of each of Alzheimer's disease and depression	and high-income neighbourhoods (OR = 0.93; 95% CI: 0.88-0.98). The same results were observed for depression, there lower odds of depression was observed for individuals living in low- (OR = 0.79; 95% CI: 0.74-0.84) and medium-income neighbourhoods (OR = 0.85; 95% CI: 0.82-0.88), than in high-income neighbourhoods (OR = 0.88, 95% CI: 0.85-0.92).
Buffel, T. & Phillipson, C., 2019 [22]	United States of America	Qualitative study	≥60	Seven focus group interviews (N=58) and 30 individual interviews	Gentrification	Three neighbourhoods in Chorlton were selected as it is an area of urban gentrification where the population grew with the influx of younger	Social exclusion	Measured by the experiences of living in the area and whether these had changed over time; views about the	Gentrification contributed to feelings of exclusion by older adults from local social relationships; however, it originated an informal network of neighbours that provided support to each other

						white-collar workers, increased property prices, and change in land uses and new "consumption spaces"		physical environment, including transportation, public space and housing; and perceptions of community life and neighbourhood relationships	
Buffel, T. et al., 2013 [23]	United Kingdom	Qualitative study	≥60	Belgium sample:102 and England sample: 124	Neighbourhood socioeconomic status/ Neighbourhood deprivation	Study areas were selected based on criteria of urban deprivation, using the Index of Local Deprivation for those in	Social exclusion	Measured by participants' experiences of neighbourhood exclusion	Elderly people felt excluded with the changing composition of their locality, felt insecure with the criminal activity of their locality, avoided certain public spaces (especially women), and coped with some of their difficulties

						England and the Atlas of Deprived Areas for those in Belgium		and inclusion	by making risk management strategies
Burns, V. F. et al., 2012 [24]	Belgium and United Kingdo m	Qualitative study	68-95	30	Gentrification	It was selected two inner-city neighbourhoo ds where local community stakeholders were concerned about current or impending gentrification (" “invasion” of previously workingclass neighbourhoo ds by middle or upper-income groups and the subsequent displacement of many of the	Social exclusion	Explore to what extent older people's social networks and activities were located inside and outside of their neighbour hood	Gentrification triggered processes of social exclusion among older adults. The elderly identified the loss of social spaces dedicated to them led to social disconnectedness, invisibility, and loss of political influence on neighbourhood planning

						original residents")			
Buys, D. R. et al., 2015 [25]	Canada	Cross-sectional study	≥65	958	Neighbourhood socioeconomic status/ Neighbourhood deprivation	Neighbourhood disadvantage index used included a composite measure of the prevalence of poverty, determined from prevalence rates of poverty and prevalence of female-headed or single-mother households at census tracts level	Hypertension	Blood pressure measurements were done near the end of the interview. Prevalent hypertension was classified when the systolic blood pressure was 140 millimetres of mercury or higher, the diastolic blood pressure was 90	Living in mid-neighbourhood disadvantage (adjusted OR= 1.6; 95% CI: 1.2-2.1) and high-neighbourhood disadvantage tertiles (adjusted OR= 1.8; 95% CI: 1.3-2.3) was associated with higher hypertension prevalence, and living in high-neighbourhood disadvantage tertiles was associated with lower odds of controlled hypertension (adjusted OR= 0.6; 95% CI: 0.4-0.6)

								millimetres of mercury or higher, or if the participant was taking antihypertensive medication	
Cadar, D., et al., 2018 [26]	United States of America	Longitudinal study	≥65	6220	Neighbourhood socioeconomic status/ Neighbourhood deprivation	Assessed based on the 2004 index of multiple deprivation for England which is a composite measure that combines multiple area-level socioeconomic status indicators into a single deprivation	Dementia	Assessed using a combination of self-reported or infor-mant-reported physician diagnosis of dementia or Alzheimer disease or a score	"Those who live in higher deprived areas have higher risk of developing dementia than those living in the lowest deprived areas, however these were no longer significant when

						score at Super Output Area Lower Layer		above the threshold of 3.38 on the16-question Informant Questionnaire on Cognitive Declinein the Elderly	
Cagney, K. A. et al., 2014 [27]	United Kingdom	individual-level SES indicators were considered. Only the association with the second-highest quintile (Q2: Hazard ratio = 1.41; 95% CI: 1.06-1.87) maintained statistical							

		significance in the fully							
Casanova, R. et al., 2020 [28]	individual-level SES indicators were considered. Only the association with the second-highest quintile (Q2: Hazard ratio = 1.41; 95% CI: 1.06-1.87) maintained statistical significance in the fully	adjusted model"							

Cerin, E. et al., 2013 [29]	adjusted model"	Longitudinal study	≥63	2 261	Neighbourhood socioeconomic status/ Neighbourhood deprivation	Changes in the percentage of poor were measured using tract data from the 2000 and 2010 censuses and data from the 2009 American Community Survey at the census tract level	Depression	A dichotomous measure derived from the Centers for Epidemiologic Studies Depression Scale	Although it was not significant, residing in an area that underwent an increase in the percentage of poor increased the odds of developing depressive symptoms (for notices of default: OR=1.04; 95% CI: 0.66-1.28; for auctions: OR=1.09; 95% CI: 0.94-1.25; for real-estate-owned: OR=1.09; 95% CI =0.94-1.26)
Chaix, B. et al., 2007 [30]	United States of America	Longitudinal study	≥65	7 142	Neighbourhood socioeconomic status/ Neighbourhood deprivation	Based on consideration of six neighbourhood characteristics at the census tract level including: the per cent of adults aged 25 years or older without a high	Cognitive decline	Measured with the abbreviated version of the modified Telephone Interview for Cognitive Status	Neighbourhood socioeconomic status was a top ranked predictor of cognitive decline (RF performance: accuracy = 78% (1.0%), sensitivity = 75% (1.0%), and specificity = 81% (1.0%)

						school education; per cent male unemployment; per cent of households with income below the poverty line; per cent of households receiving public assistance; per cent of households with a female as head-of-household; and median household income			
Chamberlain, A. M. et al., 2020	United States of America	Cross-sectional study	≥65	484	Neighbourhood socioeconomic status/	Neighbourhood socioeconomic status was	Physical exercise (walkability)	Measured by the Neighbourhood	Area socioeconomic status was unrelated to within-neighbourhood walking (OR=2.69 95%

[31]					Neighbourhood deprivation	defined using census data on median monthly household income and percentage of owner-occupiers. Area transport-related walkability was established using Census and Centamap data on household, intersection, and commercial/service densities. The neighbourhood was defined as an area approximately		Walking Questionnaire–Chinese version for Seniors	CI: 0.73-9.88) and to within-neighbourhood walking allocated to recreational purposes (OR=1.49, 95% CI: 0.57-3.90)

						15-min walk from home			
Corriere, M. D. et al., 2014 [32]	China	Longitudinal study	50-79 (Results only consulted for ≥60)	341 048	Neighbourhood socioeconomic status/ Neighbourhood deprivation	Two contextual factors were considered: population density and socioeconomic environment: Area population density was computed as the number of 50–79-year-old residents/km2. Socioeconomic environment was defined as area mean income of the 50–79-year-old residents at 1 year before baseline. The scale used was the smallest	Ischaemic heart disease mortality	Assessed with the International Classification of Diseases (ICD) as ICD-9 codes 410–414 or ICD-10 codes I20–I25	People with 65-79 years old living in a low socioeconomic environment had higher risk of dying of ischaemic heart disease (Hazard ratio=1.23, 95% CI: 1.15-1.31), compared to elderly living in a higher socioeconomic environment

						existing geographical units.			
Danielewicz, A. L. et al., 2016 [33]	Sweden	Cross-sectional study	≥20 (Results only consulted for ≥60)	198 941	Neighbourhood socioeconomic status/ Neighbourhood deprivation	Assessed with the area deprivation index that uses 17 census measures capturing education, employment, income, poverty, and housing characteristics using census block group level (n = 251 census block groups)	Multimorbidity	Assessed with the International Classification of Diseases, Ninth & Tenth Revision codes to define the 20 chronic conditions identified by the United States Department of Health and Human Services for	No difference in the risk of multimorbidity was observed across quintiles of area deprivation index, after the age of 70

								studying multimorbidity (hypertension, hyperlipidemia, diabetes, coronary artery disease, congestive heart failure, cardiac arrhythmias, stroke, asthma, chronic obstructive pulmonary disease, arthritis, osteoporosis, chronic kidney	

								disease, cancer, autism spectrum disorder, hepatitis, human immunodeficiency virus, depression, dementia, schizophrenia, and substance abuse disorders)	
Deng, G. & Mao, L., 2018 [34]	United States of America	Cross-sectional study	70–79	384	Neighbourhood socioeconomic status/ Neighbourhood deprivation	The 1990 United States Census data by block groups were used to derive the neighbourhood scores. Census was	Metabolic conditions	Metabolic conditions included: obesity (body-mass index 30 kg/m2), diabetes (self-	For obesity, the odds ratio was significantly lower for the highest neighbourhood quartile (OR=0.37, 95% CI 0.18–0.79) and second-highest neighbourhood quartile (OR=0.35, 95% CI 0.17–0.73) compared to the reference

						used to provide six measures of data that comprised the neighbourhood score, which included: median household income, median house value, per cent earning interest income, per cent completing high school, per cent completing college, and per cent with managerial or executive occupation. Neighbourhoods (block		reported history and/or use of glucose-lowering medication), hypertension (self-reported history, antihypertensive medicine use, systolic blood pressure 140 mmHg, and/or diastolic blood pressure 90 mm Hg), and hyperlipidemia (total	

						groups) had a mean population of 1000		cholesterol 200 mg/dl and/or lipid-lowering medication use)	
Diez Roux, A. V. et al., 2004 [35]	United States of America	Cross-sectional study	≥60	1 184	Neighbourhood socioeconomic status/ Neighbourhood deprivation	Assessed by categorizing into quintiles the the average monthly income of the head of each household in each of the census tracts included, according to the Brazilian Census of 2010	Cognitive decline	Assessed using the Mini-Mental State Examination. Probable cognitive decline is indicated by a score of below 19 points for those who are illiterate and below 24 points for those	The odds of cognitive decline were two times higher among elderly of lower-income census tracts (OR = 1.99; 95%CI: 1.03-3.87) compared to the elderly living in the highest quintile, regardless of age, gender, skin colour, education, individual income or length of residence in the neighbourhood

								with some education	
Domínguez-Parraga, L., 2020 [36]	Brazil	Cross-sectional study	≥65	92 560	Segregation	Spatial age segregation for metropolitan and micropolitan statistical areas was assessed through voluntary residential selection processes	Self-rated health	Self-rated health was assessed with three individual -level measures regarding the general health status and the number of healthy days that related to the quality of life	Overall, spatial age segregation was not significantly associated with older adults' general health status or with the days of physical unhealthiness but was weakly positively associated with a 0.8% increase in the number of days of mental unhealthiness.
Espino, D. V. et al., 2001 [37]	United States of America	Longitudinal study	≥65	5 074	Neighbourhoo d socioeconomic status/ Neighbourhoo d deprivation	Neighbourhoo d socioeconomic characteristics were collected from the 1990 US census.	Mortality	Reviews of obituaries, medical records, death certificates	White elderly living in the most disadvantaged neighbourhood had higher cardiovascular death rates after adjustment (hazard ratio: 1.5, 95% CI:1.2-1.9), but

						Census variables representing the dimensions of wealth/income, education and occupation were combined into the neighbourhood summary score to characterize the neighbourhood socioeconomic environment. Census block groups were used as proxies for neighbourhoods, where there is 1000		, and the US Health Care Financing Administration health care utilisation database for stays in the hospital was used to confirm deaths of participants	no association for non-cardiovascular deaths. For black elderly there was no association; however, the sample size was small

						people per block group, on average			
Espinoza, S. E. & Hazuda, H. P., 2015 [38]	United States of America	Qualitative study	≥65	32	Gentrification	Defined as a process of changes in a neighbourhoo d due to consumption or tourism. The remodelling of the area attracts more affluent people, resulting in the displacement of low-income residents. It was selected a medium-sized city - Cáceres, Spain - with 96 126 inhabitants. Two neighbourhoo	Overall well-being	Assessed the intention of elderly people to remain active taking into account social, symbolic, and spatial variables	Older adults living in gentrified neighbourhoods reported having less variety and higher prices for commerce. They also felt lonely and disconnected from their neighbourhood. In addition, older people felt dependent on family, and their activities were carried out in other parts of the city. Older adults living in non-gentrified neighbourhoods did not suffer from supply difficulties or product prices; they felt a sense of familiarity in their neighbourhood, and their activities were carried out inside the neighbourhood

						ds were selected based on being gentrified (Plaza Mayor) or non-gentrified (Peña del Cura)			
Everson-Rose, S. A. et al., 2011 [39]	Spain	Cross-sectional study	≥65	827	Neighbourhood socioeconomic status/ Neighbourhood deprivation	Neighbourhoods were classified as barrio (low-income), transitional (middle-income), or suburbs (high-income) based on census indicators: population density; cultural climate; stressors (high-income); sense of	Cognitive impairment	Assessed with the English and Spanish Mini-Mental State Examination where the cutpoint of <24 indicated the presence of cognitive	Mexican American elderly living in barrios (low-income) have three times the odds of cognitive impairment (OR= 2.99; 95% CI:1.61-5.55) compared to those living in transitional and the suburbs neighbourhoods combined

						belonging and protection; availability of consumer goods, including food, clothing, housing, and medical care; and lifestyle		impairme nt	
Fernández-Blázquez, M. A. et al., 2020 [40]	United States of America	Cross-sectional study	≥65	394	Neighbourhoo d socioeconomic status/ Neighbourhoo d deprivation	Neighbourhoo ds were classified as barrio (low-income), transitional (middle-income), or suburban based on census indicators: population density; cultural climate; stressors (high-income);	Frailty	Frailty was defined as the presence of five frailty characteris tics: walking speed standardiz ed based on median height and sex, grip strength standardiz	The odds of frailty was four times higher in elderly living in barrios compared with elderly living in the suburbs (OR=4.15; 95% CI:1.18–14.60)

						sense of belonging and protection; availability of consumer goods, including food, clothing, housing, and medical care; and lifestyle		ed based on body mass index and sex, energy expenditure standardized based on sex, exhaustion based on self-report, and weight loss. Individuals with three or more characteristics were classified as frail	

Fox, K. R. et al., 2011 [41]	United States of America	Cross-sectional study	≥65	5 770	Neighbourhood socioeconomic status/ Neighbourhood deprivation	Assessed with the census information on four indicators of the block group population: per cent on public assistance, per cent of households, earning $25,000 per year or less, per cent with a college degree or higher, and per cent of owner-occupied dwellings valued at $200,000 or higher	Psychosocial functioning	Assessed with three measures: depression symptoms (10-item version of the Center for Epidemiologic Studies Depression Scale); hostility (8 items from the Cook-Medley Hostility Scale); and perceived stress (6-item version of the Perceived	Neighbourhood socioeconomic status was inversely associated with hostility (beta=−0.133; standard error=0.062) and depressive symptoms (beta=−0.078; standard error=0.038)

								Stress Scale)	
Franse, C. B. et al., 2017 [42]	United States of America	Longitudinal study	≥70	1 180	Neighbourhood socioeconomic status/ Neighbourhood deprivation	The neighbourhood socioeconomic status index was based on the standardisation as a z-score of the: average annual net income, % of residents with no formal qualifications, % of residents with higher education, % of residents with white-collar jobs, unemployment rate, and housing price and slipt into quartiles with	Cognitive impairment	Cognitive diagnoses were agreed between neurologists and neuropsychologists at each visit, taking into account his/her age, gender, cognitive reserve, functional information, and cognitive scores	Based on the characteristics of the neighbourhood, a higher risk of developing cognitive impairment was observed for lower quartiles than upper quartiles; however, the association was not significant (Hazard ratio=1.08 95% CI: 0.62-1.89 for quartile 1)

						the first quartile representing the worst using as the unit of analysis the administrative boundaries of the city of Madrid			
Gale, C. R. et al., 2011 [43]	Spain	Cross-sectional study	≥70	240	Neighbourhood socioeconomic status/ Neighbourhood deprivation	Assessed through the Index of Multiple Deprivation, which includes factors such as: income, employment, health, education, housing, environment, and crime using the Lower Level	Physical activity	Measured by an accelerometer for seven days	There was no association between neighbourhood deprivation and physical activity when controlling for age, gender, and level of educational attainment

						Super Output Areas			
Garcia, L. et al., 2015 [44]	United Kingdom	Cross-sectional study	≥55 (Results only consulted for ≥60)	26 014	Neighbourhood socioeconomic status/ Neighbourhood deprivation	Neighbourhood socioeconomic status was defined using the education level, income and labour market position of persons living in each area code. Scores were categorized into quartiles; quartile 1 is the least deprived quartile	Frailty	Assessed with the 45 items TOPICS-Frailty Index, where higher scores represent higher frailty	Elderly who live in the most deprived quartile had worst outcomes in frailty when compared to elderly living in the least deprived neighbourhoods (For 70-79 years: beta=0.014; 95% CI:0.008-0.019)
Garcia, L. et al., 2016 [45]	Netherlands	Cross-sectional study	69-78	1 157	Neighbourhood socioeconomic status/ Neighbourhood deprivation	Assessed with the 2001 census lower super output areas: neighbourhoo	Mental health	Assessed with the Warwick-Edinburgh Mental Mental	No association between level of deprivation in the area of residence and mental wellbeing score was observed (regression

						d deprivation (Index of Multiple Deprivation 2007 - income, employment, health and disability, education, skills and training, barriers to housing and services, crime, and living environment)		wellbeing Scale, which includes: positive affect, psychological functioning, and interpersonal relationships	coefficient -0.041; 95% CI: -0.11 to 0.02)
Giehl, M. C. G. et al., 2016 [46]	United Kingdom	Cross-sectional study	≥60	1 777	Neighbourhood socioeconomic status/ Neighbourhood deprivation	Neighbourhood socioeconomic position was based on census tracts: the percentage of individuals 25 years of age or older without a high school	Diabetes	Type-2 diabetes was assessed as fasting glucose level 126 mg/dl, self-report of a physician diagnosis	Higher neighbourhood socioeconomic status seems to have a protective effect against diabetes (0.76; 95% CI: 0.62-0.94); however, this association was no longer significant if we considered only the United States born participants. No association was observed

						diploma; the percentage of the population living below the poverty line; the percentage of individuals 16 years of age who at one time had been in the workforce and who were unemployed; the percentage of households that owned their home, percentage of housing units that were vacant; and the median number of rooms in the household. Higher scores		of diabetes, and/or document ed use of prescriptio n diabetes medicatio n. Pre-diabetes as fasting blood glucose level between 100 mg/dL and 125 mg/dL	between neighbourhood socioeconomic position and pre-diabetes

						indicate higher neighbourhood socioeconomic status			
Guo, Y. et al., 2019 [47]	United States of America	Longitudinal study	≥60	1 777	Neighbourhood socioeconomic status/ Neighbourhood deprivation	Neighbourhood socioeconomic position was based on census tracts: the percentage of individuals 25 years of age or older without a high school diploma; the percentage of the population living below the poverty line; the percentage of individuals ≥16 years of age who at one time had been	Diabetes	Type-2 diabetes was assessed as: fasting glucose level ≥ 126 mg/dl, self-report of a physician diagnosis of diabetes, documented use of prescription diabetes medication or diabetes listed as a cause of	Among non-diabetics, higher neighbourhood social position was not associated with a transition to pre-diabetes but was associated with an increased risk of diabetes (HR=1.66, 95% CI: 1.14-2.42) and decreased risk of death without diabetes (HR: 0.56, 95% CI: 0.33-0.96)

						in the workforce and who were unemployed; the percentage of households that owned their home, percentage of housing units that were vacant; and the median number of rooms in the household. Higher scores indicate higher neighbourhoo d socioeconomic status		death. Pre-diabetes as fasting blood glucose level between 100 mg/dL and 125 mg/dL	
Guo, Y. et al., 2019 [48]	United States of America	Cross-sectional study	≥60	1 705	Neighbourhoo d socioeconomic status/ Neighbourhoo d deprivation	Assessed using census tracts: land-use mix, street density, street connectivity,	Physical activity	Assessed through two measures: walking for	Elderly living in areas with higher population density (OR=2.19; 95% CI:1.40–3.42), with higher street connectivity (OR=1.85; 95% CI: 1.16–

						and public open spaces and also using 2010 Census data on: area income, population density, percentage of street lighting, percentage of paved streets, and percentage of sidewalks		transportation and for leisure based on the long version of the International Physical Activity Questionnaire	2.94), a higher sidewalk proportion (OR=1.77; 95% CI: 1.11–2.83), and paved streets (OR=2.11; 95% CI:1.36–3.27) were more likely to walk for transportation. Elderly living in a medium-income area (OR=1.48; 95% CI:1.04–2.12), and medium population density (OR=1.47; 95% CI: 1.02-2.10) were more likely to walk for leisure.
Hannon, L. 3rd, et al., 2012 [49]	Brazil	Cross-sectional study	≥65	21 008	Neighbourhood socioeconomic status/ Neighbourhood deprivation	Neighbourhood economic disadvantage was measured using neighbourhood poverty rate using census tracts	Cognitive function	Measured using the Mini-Mental State Examination. Cognitive impairment was defined as	Neighbourhood economic disadvantage was significantly associated with cognitive decline

								a score <24; mild dementia scores between 19-23; moderate dementia 10–18; and severe dementia 0–9	
Hawkesworth, S. et al., 2018 [50]	China	Cross-sectional study	≥65	29 099	Neighbourhood socioeconomic status/ Neighbourhood deprivation	The neighbourhood social attributes were based on census data: neighbourhood poverty, ethnic minority, residential stability and elderly concentration; and the neighbourhoo	Depression	Measured with a 15-item Geriatric Depression Scale Short Form ranging from 0 to 15, where a value of 8 or above would suggest	Neighbourhood poverty positively associated with both depressive symptoms (coefficient=0.012; 95% CI 0.009 to 0.015) and depression (OR=1.016; 95% CI: 1.010 to1.022). Residential recreation was positively associated with depressive symptoms (coefficient: 0.009; 95% CI: 0.007 to 0.011) and negatively associated with elderly concentration

						d physical attributes were based on: neighbourhood recreational environment (population-weighted average road network distance to the nearest recreational services) and walkability [walk score - 0 (the lowest walkability) to 100 (the highest walkability)]		depression	(coefficient=-0.004; 95% CI: -0.005 to 0.003) and neighbourhood walkability (coefficient=-0.004; 95% CI: -0.006 to 0.002)
Hazzouri, A. Z. A. et al., 2011 [51]	China	Cross-sectional study	≥65	433	Neighbourhood socioeconomic status/ Neighbourhood deprivation	Measured with the per cent of owner-occupied housing within specific census tracts, the per	Physical exercise	Measured with the question: "During the last week, about how	Homeownership (coefficient=0.105; p<0.05) and occupancy (coefficient=0.108; p<0.01) were positively associated with neighbourhood walking

						cent of occupied housing in the community in contrast to vacant housing, and neighbourhood median home value using census tracts data		many blocks did you walk?" and organised into four categories: 1 - 0 blocks; 2 - 1-6 blocks per week; 3 - 7-14 blocks per week; 4 - > 15 blocks per week	
Huang, Y. et al., 2018 [52]	United States of America	Cross-sectional study	69–92	1 433	Neighbourhood socioeconomic status/ Neighbourhood deprivation	Measured through area social deprivation (2010 Index of Multiple Deprivation and the 2009 Scottish Index of Multiple Deprivation)	Physical activity	Measured with a GT3x accelerometer over the right hip for seven days during waking	Higher area income deprivation was associated with lower levels of activity and less time spent in moderate to vigorous physical activity

						using Lower Layer Super Output Areas as the geographical units		hours (100–1040 minutes for light-intensity physical activity and > 1040 for moderate to vigorous physical activity)	
Hybels, C. F. et al., 2006 [53]	United Kingdo m	Longitudinal study	60–101	1 789	Neighbourhoo d socioeconomic status/ Neighbourhoo d deprivation	Measured by a neighbourhoo d socioeconomic position score using census tracts: percentage of individuals 25 years of age or older without a high school diploma, percentage of	Cognitive decline	Measured with the Modified Mini-Mental State Examinati on	Lower neighbourhood socioeconomic position was associated with poorer baseline cognitive scores, but neighbourhood socioeconomic position did not appear to directly influence cognitive decline after adjustment for individual-level education or occupation

						the population living below the poverty line, percentage of individuals 16 years of age or older who at one time had been in the workforce and who were unemployed, percentage of households that owned their home, percentage of vacant housing units, and the median number of rooms in the household. Higher scores indicated higher/better			

						Neighbourhood socioeconomic position			
Joshi, S. et al., 2017 [54]	United States of America	Longitudinal study	≥65	13 686	Neighbourhood socioeconomic status/ Neighbourhood deprivation	Measured at the zip code level and based on: per cent elderly Hispanic, per cent elderly population with less than a high school education, per cent elderly population below poverty level, per cent elderly disability status by health insurance coverage status, per cent elderly living alone elderly	Access to healthcare	Assessed with emergency department visits from all local hospital systems serving the Coastal Bend area from September 1, 2009 to August 31, 2012	Higher rates of visits for ambulatory care sensitive conditions were associated with higher rates of elderly Hispanic (coefficient=0.101) and poverty level (coefficient=0.306) at the zip code level

						population, and per cent elderly population who speak Spanish			
Jung, D. et al., 2018 [55]	United States of America	Cross-sectional study	≥65	2 998	Neighbourhood socioeconomic status/ Neighbourhood deprivation	The neighbourhood sociodemographic characteristics were measured using census tract-level variables: socioeconomic disadvantage (per cent of individuals in census tract living in poverty), socioeconomic advantage (per cent of families in a census	Depressive symptoms	Measured using a modified version of the 20-item Center for Epidemiologic Studies–Depression scale with a range of 0 to 20 and where participants with a score of >9 are considere	There was not a significant association between neighbourhood characteristics and depressive symptoms

						tract with an annual income of >$75,000), racial/ethnic heterogeneity (per cent black households in census tract), residential stability (per cent persons in census tract living in the same house for >5 years), and age structure (per cent persons in census tract age >65)		d depressed	
Kelley-Moore, J. A. et al., 2016 [56]	United States of America	Longitudinal study	65–75	3 497	Neighbourhood socioeconomic status/ Neighbourhood deprivation	Assessed with the per cent of households in each participant's 1-km network buffer living below the	Depression	Measured with the 9-item Patient Health Questionnaire that assesses	Neighbourhood poverty was positively associated with depressive symptoms (RR=1.19; 95% CI: 1.02-1.39)

						federal poverty level using the American Community Survey 5-year estimates from 2006–2010 based geographically at the lower level Super Output Area		depressive symptoms in the past month	
Kim, G. H., et al., 2017 [57]	United States of America	Cross-sectional study	≥65	187 434	Neighbourhood socioeconomic status/ Neighbourhood deprivation	Assessed using the 2013 Area Deprivation Index, which includes: poverty, education, housing, and employment indicators at the census block group level	Functional limitation	Measured with difficulty in 1 or more of 6 self-reported activities of daily living (ADL) OR 4 instrumental ADL	Elderly with multiple chronic conditions living in less affluent neighbourhoods had higher odds of reporting a functional limitation (OR=1.29; 95% CI=1.11–1.49) when compared to elderly living in more affluent neighbourhoods

Ko, J. E. et al., 2014 [58]	United States of America	Cross-sectional study	≥65	5 625	Neighbourhood socioeconomic status/ Neighbourhood deprivation	Assessed through two dimensions: socioeconomic status (measured by income) and social integration (assessed with the question: "How many of your neighbours do you know well enough to visit with inside your home at least once-a-month?") using census block group	Mental Health	Assessed with the 10-item Center for Epidemiologic StudiesDepression scale	Neighbourhood socioeconomic status was significantly negatively associated with depressive symptoms (coefficient=-0.003; standard error=0.001) as well as neighbourhood social integration (coefficient=-0.006; standard error=0.001)
Kubzansky, L. D. et al., 2005 [59]	United States of America	Longitudinal study	≥60	136217	Neighbourhood socioeconomic status/ Neighbourhood deprivation	Assessed using district-level socioeconomic status based on the tax income of each	Cognitive decline	Diagnosed if participants scored below a 1.5	Elderly living in a lower district-level socioeconomic status have a higher risk of cognitive impairment when compared to

						district per year in Seoul		standard deviation on the mini-mental status examination	people living in higher district-level socioeconomic status (RR = 1.1; p-value: 0.026)
Kwag, K. H. et al., 2011 [60]	South Korea	Cross-sectional study	≥65	1 382	Neighbourhood socioeconomic status/ Neighbourhood deprivation	Assessed with the 2000 U.S. Bureau of the Census Summary Files and includes: the proportion of individuals age 65 and older, the proportion of individuals below poverty, and the proportion of individuals from the same racial/ethnic background	Self-rated health	Assessed by asking participats to ate their health, using a four point rating system (poor = 1, fair = 2, good = 3, excellent = 4)	Living in a neighbourhood with a higher proportion of below-poverty residents was associated with reporting poorer health regardless of race. For Cubans, the proportion of older adults in the neighbourhood was positively associated with self-rated health (β=0.01; standard error=0.01), and the proportion of residents with the same ethnic background was only positively in Whites (β=0.004; standard error=0.001)

						using census block group			
Lager, D. et al., 2013 [61]	United States of America	Cross-sectional study	≥65	1 884	Neighbourhood socioeconomic status/ Neighbourhood deprivation	Assessed using: neighbourhood socioeconomic disadvantage (percentage of people living in poverty), racial/ethnic heterogeneity (percentage of Black residents), residential stability (percentage of individuals who have been in their homes longer than 5 years), age structure (percentage of individuals aged over 64	Depressive symptoms	Assessed with the 20-item Center for Epidemiologic Studies Depression Scale where high scores represent more depressive symptoms	Elderly living in neighbourhoods with a higher rate of poverty had more depressive symptoms than did those living in census tracts with lower levels of poverty (b=6.51; 95% CI:1.02 to 12.00). Elderly living in areas with higher concentration of individuals over 64 years had lower levels of depression (b=-13.55; 95% CI:-24.76 to -2.34) compared to elderly living in areas with younger people. There was no evidence for an association between service density measures and depressive symptoms

						years), and socioeconomic advantage (affluence; percentage of individuals with income greater than $75,000 per year). In addition, it was developed a neighbourhood-level measures of service density using the Yellow Pages. Measured using census tracts as proxies for neighbourhoods			
Lang, I. A. et al., 2008 [62]	United States of America	Cross-sectional study	60-96	567	Neighbourhood socioeconomic	Measured using census block groups :	Physical and mental health	It was measured participan	Elderly living in poorer neighbourhoods have worst health perceptions

					status/ Neighbourhood deprivation	the proportion of individuals living below the poverty level, the proportion of individuals 65 years of age and older, and the proportion of racial/ethnic minorities		ts' subjective health perceptions based on the Older American Resources and Services Questionnaire with scores ranging from 0 to 7 where higher scores indicate more negative health perceptions. In addition, depressive symptoms were	(coefficient=1.39; standard error=0.69)

								measured using the 15-item short form of the Geriatric Depression Scale with scores ranging from 0 to 15 where higher scores indicate more depressive symptoms	
Lang, I. A. et al., 2008 [63]	United States of America	Qualitative study	≥65	13	Urban Renewal	The Oosterpark working-class neighbourhood (11 575 residents, 8% ≥65) was chosen as it is suffering a	Ageing in place	Defined as "growing old in one's own home and neighbourhood"	Older adults identified that the changes had limited their opportunities for informal encounters and support; it also shaped the neighbourhood space and negotiating place identity

						neighbourhood renewal process to improve the liveability of disadvantaged neighbourhoods			
Lang, I. A. et al., 2008 [64]	Netherlands	Cross-sectional study	≥52 (Results only consulted for ≥60)	7 126	Neighbourhood socioeconomic status/ Neighbourhood deprivation	Measured at the Super Output Area level by the Index of Multiple Deprivation 2004, which includes: income deprivation; employment deprivation; health deprivation and disability; education, skills, and training deprivation;	Cognitive function	Measured by using day and date questions from the Mini-Mental State Examination to check time orientation; 10-word learning task from the Health and Retirement Study to	The neighbourhood index of multiple deprivation was not significantly associated with cognitive function in people aged 70 and older

						barriers to housing and services; living environment deprivation; and crime		see verbal memory; remembering a task to perform later to assess prospective memory; thinking od words of a category to check for verbal fluency; and letter cancellation task from the National Study of Health and Development to measure attention	

								and mental speed	
Lang, I. A. et al., 2009 [65]	United Kingdom	Longitudinal study	≥60	4 148	Neighbourhood socioeconomic status/ Neighbourhood deprivation	Measured at the Super Output Area level by the Index of Multiple Deprivation 2004, which includes: income deprivation; employment deprivation; health deprivation and disability; education, skills, and training deprivation; barriers to housing and services; living environment	Mobility disability	Mobility difficulties were self-reported by questioning if participants at wave 2 (who did not report difficulties at wave 1) experienced difficulties walking 100 yards or climbing one flight of stairs without resting due to	After 2-year follow up, elderly living in the most deprived areas have a higher risk of having mobility difficulties (Risk ratio=1.75: 95% CI: 1.14-2.70) and a higher risk of presenting impaired gait speed (Risk ratio=1.63; 95% CI: 1.01-2.62) compared to individuals living in the least deprived areas

						deprivation; and crime		health problems for at least more than 3 months. Gait speed was measured with a test that assessed how long it took to walk a distance of eight feet	
Lawlor, D. A., et al., 2005 [66]	United Kingdo m	Cross-sectional study	≥65	4 240	Neighbourhoo d socioeconomic status/ Neighbourhoo d deprivation	Measured using the super output area with the Index of Multiple Deprivation 2004, which includes: income; employment; health and	Dental service use	Measured with questions related to the presence of participan ts own teeth or if they have already	Elderly living the 20% most deprived areas have 2.25 times more risk of using dental services only when symptomatic than elderly living in the 20% least deprived neighbourhoods (Relative risk ratio=2.25; 95% IC: 1.59-3.17)

						disability; education, skills and training; barriers to housing and services; living environment; and crime		lost all of them. In case participants had their own teeth it was asked: ‘in general, do you go to the dentist for a regular check-up or only when you are having trouble with your teeth?’ where possible answers were: ‘regular check-up’, ‘occasiona	

								l check-up', 'only when having trouble' and 'never go to the dentist'. Responses were coded into two categories: asymptomatic visiting (regular or occasional check-ups) and symptomatic visiting (only when having trouble or never attending)	

Letellier, N. et al., 2017 [67]	United Kingdo m	Cross-sectional study	≥65	4 818	Neighbourhoo d socioeconomic status/ Neighbourhoo d deprivation	Measured at the Super Output Area level by the Index of Multiple Deprivation 2004, which includes: income deprivation; employment deprivation; health deprivation and disability; education, skills, and training deprivation; barriers to housing and services; living environment deprivation; and crime	Frailty	The frailty index was calculated based on sensory and functional impairme nts, self-reported comorbidi ties, poor or fair self-rated health, low mood or depressio n measured according to the eight-item Center for Epidemiol ogical Studies Depressio	Elderly living in the most deprived areas are more vulnerable to be frail (coefficient=0.032: 95% CI: 0.019-0.045) compared to individuals living in the least deprived areas

								n Scale and a score in the lowest 10% of composite cognitive function testing. This index ranged from 0 to 1 where higher values indicated greater frailty	
Letellier, N. et al., 2019 [68]	United Kingdo m	Cross-sectional study	≥60	4286	Neighbourhoo d socioeconomic status/ Neighbourhoo d deprivation	Assessed using the Carstairs deprivation score which is based on 4 variables derived from census data: male unemploymen	Coronary heart disease	Assessed with participan ts' medical records or by self-report of a physician diagnosis	Elderly living in wards with a deprivation score above the median had higher odds of having a coronary heart disease when compared to elderly living in wards with a deprivation score equal or below the

						t, household overcrowding, car ownership, and the proportion of households in social classes IV (semi-skilled manual occupations) and V (unskilled manual occupations), at the electoral ward geographical level			median (OR = 1.27; 95% CI: 1.02-1.57)
Li, W. et al., 2014 [69]	United Kingdo m	Longitudinal study	≥65	7 016	Neighbourhoo d socioeconomic status/ Neighbourhoo d deprivation	Measured based on median household net taxable income per consumption unit (CU), interquartile range of the	Dementia	Participan ts were examined by a neurologis t and an independe nt committee of	Elderly women living in the most deprived neighbourhoods have higher risk of dementia (hazard ratio=1.29; 95% CI:1.00-1.67) and Alzheimer's disease (hazard ratio=1.42; 95% CI:1.09-1.84) compared to women living in the

						household net taxable income per CU, interdecile ratio of household net taxable income per CU, proportion of blue collar workers, proportion of residents without secondary education, proportion of tenant occupancy, proportion of households without a car, proportion of single parents, proportion of people aged 60 or over, Gini index (an		neurologists according to the Diagnostic and Statistical Manual of Mental Disorders IV criteria. Participants with Alzheimer's disease were classified according to the National Institute of Neurological and Communicative Disorders and Stroke	least deprived areas. There was no association observed in men.

						indicator of income inequality), adult unemployment rate, and settlement index (ratio between overcrowded and non-overcrowded housing) and categorised into tertiles		and the Alzheimer's Disease and Related Disorders Association criteria	
Lo, A. X. et al., 2016 [70]	France	Longitudinal study	≥65	7 009	Neighbourhood socioeconomic status/ Neighbourhood deprivation	Measured based on the proportion of households without a car, of tenants and single parents, Gini index (an indicator of income inequality), unemployment rate, and	Dementia	Participants were evaluated by a trained psychologist with a neuropsychological tests Mini-Mental State 130 Examinati	For deprived neighbourhoods, the risk of developing dementia is higher in neighbourhoods with high proportion of car-free households (> 29.0%; T3) than for those in neighbourhoods with a low proportion (< 21.2%; T1), but only if their activity space was limited (T3 adjusted

						settlement index; and a negative score for the tax household income		on and reviewed by an independe nt committee of neurologis ts to obtain a consensus on the diagnosis according to the DSM-IV criteria	HR=1.42; 95% CI 1.00–2.03). When Gini index was high and there was limited activity space the risk of dementia was also higher (T3 adjusted HR = 1.60; 95% CI 1.04–2.45). For advantaged neighbourhoods with limited activity space, where the median household net taxable income per consumption unit was higher than 15,500 € (T3 adjusted HR=0.68; 95% CI = 0.46–1.00) and when the neighbourhood had a higher proportion of people over 60 years (T3 adjusted HR =0.71; 95% CI = 0.51–0.99)
Lönn, S. L. et al., 2019 [71]	France	Longitudinal study	≥65	765	Neighbourhoo d socioeconomic status/ Neighbourhoo d deprivation	The neighbourhoo d socioeconomic status was assessed at	Falls	Self-reported falls were assessed monthly. A fall was	Higher rates of falls on sidewalks, streets, and curbs, were significantly associated with many block group-level lower-SES indicators (e.g. %

						census block group level through the 5-year rolling averages of American Community Surveys 2005 to 2009		defined as being an event that resulted in unintentionally coming to rest on the ground or a lower surface	with income < poverty level rate ratio=1.75; 95% CI:1.27-2.40)
Meijer, M. et al., 2012 [72]	United States of America	Longitudinal study	≥65	940	Neighbourhood socioeconomic status/ Neighbourhood deprivation	Measured using census tracts on: the prevalence of poverty - a marker of economic disadvantage - (determined from the number of households living below the poverty line in 2000) and the prevalence of	Falls	Based on: muscle weakness according to self-report, history of falls in the past year, gait deficit (gait speed <0.8 m/s), balance deficit according to in-	More disadvantaged neighbourhoods have higher odds of reporting more falls (OR=3.20; 95% CI: 1.71-5.97)

						female-headed households - a marker of social disadvantage - (defined as households with children under 18 years old without an adult male member, in the census tract)		person examination, use of an assistive device according to in-person assessment, visual deficit as determined using a standardized visual acuity examination, history of arthritis according to self-report, self-reported impairment in one or more	

								activity of daily living, depression based on the 15-item Geriatric Depression Scale, and cognitive impairment based on the Mini-Mental State Examination	
Menec, V. H. et al., 2010 [73]	United States of America	Longitudinal study	≥45 (Results only consulted for ≥60)	3 140 657	Neighbourhood socioeconomic status/ Neighbourhood deprivation	Assessed by the Small Areas for Market Statistics obtained from Statistics Sweden. It was	Coronary heart disease	Measured by having a coronary heart disease within five years after	Men 60 years and older and women 60 years and older living in a more deprived neighbourhood had higher odds for coronary heart disease than those living in the least deprived

						based on: the proportion of people residing in the neighbourhood with low income, low education, unemployment and receipt of social welfare		baseline based on WHO's International Classification of Diseases (ICD), that is, ICD-7 code 420, ICD-8 and ICD-9 codes 410, 411, 412, 413 and 414, and ICD-10 codes I20, I21, I22, I23, I24 and I25	neighbourhoods, with ORs ranging from 1.12 (95% CI: 1.05-1.20) to 1.24 (95% CI: 1.16-1.32) for men and ranging from 1.20 (95% CI: 1.12-1.28) to 1.43 (95% CI: 1.30-1.52) for women. In addition, people 60 years and older living a longer time in a deprived neighbourhood increases the odds of having coronary heart disease (For men ranging from OR=1.11; 95% CI: 1.06-1.19 to OR=1.30; 95% CI: 1.13-1.25 and for women ranging from OR=1.11; 95% CI: 1.06-1.16 to OR=1.30; 95% CI: 1.22-1.40) compared to living in the least deprived neighbourhood
Merkin, S. S. et al., 2007 [74]	Sweden	Longitudinal study	30–81 (Results only consulte	2 731 276	Neighbourhood socioeconomic status/	Assessed with parish-level socioeconomic status based	All-cause mortality	Extracted from Statistics Denmark	Higher mortality was associated with individuals who lived in areas with many

			d for ≥60)		Neighbourhood deprivation	on individual-level data of inhabitants such as: average education length, average disposable income level, proportion of unemployed, proportion of semi/ unskilled workers and proportion disability pensioners		from January 1st 2004 to December 31st 2006	unemployed inhabitants (Between 65-81: HR=1.05; 95% CI=1.02–1.07)
Meyer, O.L. et al., 2017 [75]	Denmark	Cross-sectional study	≥65	77 930	Neighbourhood socioeconomic status/ Neighbourhood deprivation	Measured by using neighbourhood income, percentage of individuals aged 65 or older, and percentage of	Health conditions	It was chosen 12 health measures including: arthritis, diabetes, hypertension,	Compared to those residing in the wealthier areas, people living in the poorest areas had higher odds for having worst health (arthritis: OR=1.15; diabetes: OR=1.47; hypertension: OR=1.19; congestive

						individuals who moved in the past 5 years and the geographic unit used was the enumeration area		congestive heart failure, ischemic heart disease, and chronic obstructive pulmonary disease (chronic physical health conditions), dementia and depression (mental health conditions), acute myocardial infarction, stroke,	heart failure: OR=1.53; ischemic heart disease: OR=1.37; chronic obstructive pulmonary disease: OR=1.56 and depression: OR=1.19)

								falls, and hip fractures (acute conditions)	
Miao, J. et al., 2019 [76]	Canada	Longitudinal study	≥65	4 735	Neighbourhood socioeconomic status/ Neighbourhood deprivation	Measured using summary scores of: median household income; median value of housing units; percent households with interest, dividend or rental income; percentage of residents over age 25 with complete high school education;	Progressive chronic kidney disease	Assessed with serum creatinine increase ≥0.4 mg/dL at any follow-up visit compared to baseline or a hospitalization discharge or death coded for conditions related to chronic	After fully adjusted, elderly living in the lowest socioeconomic areas have a higher risk of having progressive chronic kidney disease than elderly living in the highest socioeconomic areas(Hazard ratio=1.4; 95% CI: 1.0-1.7)

						percentage of residents over age 25 with complete college education; percentage of residents in executive, managerial, or professional specialty occupations at the census block groups level		kidney disease	
Michael, Y. L. et al., 2014 [77]	United States of America	Longitudinal study	65–94	2 438	Neighbourhood socioeconomic status/ Neighbourhood deprivation	Measured through the 2000 U.S. Census tracts and 2002 Economic Census using: income, education, and occupation,	Cognitive function	Assessed with: memory, reasoning, processing speed, and everyday cognition	No significant neighbourhood socioeconomic position was associated with cognitive decline

						median household income, percentage of people with income ≥US$150,000, percentage of people with ≥bachelor's degree, and percentage of the workforce in management positions or higher			
Mooney, S. J. et al., 2017 [78]	United States of America	Cross-sectional study	≥60	2 167	Neighbourhood socioeconomic status/ Neighbourhood deprivation	Measured using neighbourhood socioeconomic status index which included: the proportion of the population with at least	Depression	Measured using the 10-item Hopkins Symptom Checklist considering two dimensions of mental	Neighbourhood socioeconomic status is directly and indirectly linked with depression. While living in poorer neighbourhoods increase the vulnerability to depression (direct path coefficient= -0.27), those who report a higher level of interaction with their

						senior high school education, the proportion of the population in high status occupations (i.e., managers, administrators, and professionals), and the proportion of the population holding a urban hukou (the place of hukou registration defines whether a person is counted as a migrant and whether he/she can access welfare and services in		disorder: anxiety (4 questions) and depressio n (6 questions)	neighbours benefit the elderly's psychological well-being (indirect path= -0.05)

						a specific locality)			
Moser, A. et al., 2014 [79]	China	Longitudinal study	≥66	2 003	Neighbourhood socioeconomic status/ Neighbourhood deprivation	Measured using 1990 block group census measures of unemployment, occupation in managerial or professional roles, poverty, education, median home price, and median household income	Obesity	Measured by using participants' body mass index, where a BMI of ≥25 was classified as overweight and a BMI of ≥30 was classified as obesity	Elderly living in a higher socioeconomic status neighbourhood was associated with having a healthier body mass index at baseline and protected against age-related weight loss over time
Mõttus, R. et al., 2012 [80]	United States of America	Cross-sectional study	65-75	3 497	Neighbourhood socioeconomic status/ Neighbourhood deprivation	Assessed using the 2006–2010 American Community Survey to check demographic and economic characteristics.	Physical activity	Measured by using 16 items derived from the Physical Activity Scale for the	The proportion of people living in extremely poor neighbourhoods is strongly associated with less total physical activity -0.85 (95% CI:-1.14 to -0.56)

						The residential neighbourhood was defined as the land area reachable by city streets within a 0.25 km network buffer from the geocoded home location of the participant		Elderly to assess past-week physical activity	
Nguyen, H. V., 2016 [81]	United States of America	Longitudinal study	≥30 (Results only consulted for ≥60)	4 526 759	Neighbourhood socioeconomic status/ Neighbourhood deprivation	Neighbourhood index of SEP was based on the median rent per square metre, the proportion of households headed by a person with primary education or less, the proportion headed by a	Life expectancy	Life expectancy at age 30 was estimated using a skew-normal regression approach accounting for left-truncated and right-censored	Comparing the 1st and 99th percentile of neighbourhood SEP there are differences in the number of additional life expectancy. Although differences across percentiles of the Swiss-SEP index were reduced in higher ages, there were still substantial differences.

						person in a manual or unskilled occupation and the mean number of persons per room. The index was standardised to range from 0 (lowest SEP) to 100 (highest SEP). Neighbourhoods were defined as about 50 households.		observations	
Nicklett, E. J. et al., 2011 [82]	Switzerland	Longitudinal study	68-71	1 091	Neighbourhood socioeconomic status/ Neighbourhood deprivation	Measured using Scottish data zones with the Scottish Index of Multiple Deprivation using measures such	Quality of life	Measured by the 26-item WHOQOL-BREF that groups into 4 major domains	Neighbourhood deprivation was significantly associated with self-perceived quality of life in physical ($\beta = 0.07$, $p < 0.05$) and environmental domains ($\beta = 0.16$, $p < 0.001$)

						as: average income, employment, health, education, skills and training, housing, geographic access, and crime		of life quality: physical (7 items), psychological (6 items), social (3 items), and environmental (8 items)	
Nordstrom, C. K. et al., 2004 [83]	Scotland	Cross-sectional study	16–85 (Results only consulted for ≥60)	98 468	Neighbourhood socioeconomic status/ Neighbourhood deprivation	Measured at commune level based on household expenditures of participants (income, consumption and several other items belonging to households such as houses, land, durable goods)	Hypertension	Based on self-report and objective measurement of hypertension (recorded three times by a medical doctor). Hypertension was	There was no statistically significant relationship between neighbourhood wealth and hypertension for people above 65 years old

						and calculating then the neighbourhood wealth as the average of the household expenditures of all of his neighbours in his commune of residence		categorised into 4 categories: no hypertension, prehypertension (at least 2 readings of ≥120), hypertension (3 readings of ≥140, and very high hypertension (at least 2 readings of ≥160)	
Nordstrom, C. K. et al., 2007 [84]	Vietnam	Longitudinal study	70–79	408	Neighbourhood socioeconomic status/ Neighbourhood deprivation	Measured at the census block level by using a summary neighbourhood score based	Diet	Serum carotene, b-carotene, b-cryptoxanthin,	High neighbourhood socioeconomic status is positively associated with serum a-carotene (P= 0.0006), b-carotene (P= 0.07), b-cryptoxanthin (P= 0.03),

						on: log median household income; log median value of housing units; percentage of households receiving interest, dividend, or net rental income; the percentage of adults 25 years of age or older who had completed high school; the percentage of adults 25 years or older who had completed college; and occupation (the percentage of		lutein+zeaxanthin, and lycopene were analysed in nonfasting participants from blood samples	and lutein+zeaxanthin (P= 0.004)

						employed persons 16 years of age or older in executive, managerial, or professional specialty occupations)			
Omariba, W. R., 2010 [85]	United States of America	Cross-sectional study	≥65	3 545	Neighbourhood socioeconomic status/ Neighbourhood deprivation	Assessed at the census block level with a neighbourhood score by combining variables derived from1990 US Census data: Neighbourhood income and wealth (log of the median household income, log of the median value of	Cardiovascular disease	Measured with the composite index of subclinical atherosclerosis and cardiovascular disease developed in the Cardiovascular Health Study	No significant neighbourhood socioeconomic position was associated with subclinical disease

						housing units, and the percentage of households receiving interest income); Neighbourhood education (the percentage of adults 25 years of age or older who had completed high school and the percentage of adults 25 years of age or older who had completed college); and Neighbourhood occupation (the percentage of employed			

						persons 16 years of age or older in executive, managerial, or professional specialty occupations)			
Ostir, G. V. et al., 2003 [86]	United States of America	Longitudinal study	≥65	3 684	Neighbourhood socioeconomic status/ Neighbourhood deprivation	Based on census defined block-groups with a neighbourhood score by combining variables derived from1990 US Census data: Neighbourhood income and wealth (log of the median household income, log of the median value of housing units,	Physical function	Mobility impairment was self-reported and considered if the participant reported difficulty either: walking a half-mile or 2) walking up ten steps	Living in poorer neighbourhoods was associated with an increased risk of incident mobility disability during the 10-year follow-up period

						and the percentage of households receiving interest income); Neighbourhood education (the percentage of adults 25 years of age or older who had completed high school and the percentage of adults 25 years of age or older who had completed college); and Neighbourhood occupation (the percentage of employed persons 16			

						years of age or older in executive, managerial, or professional specialty occupations)			
Patel, K. V. et al., 2003 [87]	United States of America	Cross-sectional study	≥65	80 865	Neighbourhood socioeconomic status/ Neighbourhood deprivation	Measured using: median of the total income of all households in a neighbourhood, education, non-family persons, visible minorities, recent immigrants	Self-rated health	Assessed with the question: ''In general, would you say your health is: Excellent, Very good, Good, Fair, or Poor?'' and grouped into three categories: ''Fair or poor'',	Regarding negative self-rated health, elderly with the lowest household income (OR=1.25; 95% CI: 1.09-1.42) and with the lowest education level are more likely to report fair or poor health than good health (OR=1.13; 95% CI: 1.04-1.22), while positive self-rated health has the mirror effect

								‘’Good’’ (reference group), and ‘’Very good or excellent’	
Pearce, J. et al., 2018 [88]	Canada	Cross-sectional study	≥65	3 050	Neighbourhood socioeconomic status/ Neighbourhood deprivation	Measured using data at the census tracts-level from the 1990 census and included: residential stability, percentage Mexican American (higher scores indicate greater homogeneity), and socioeconomic status the percentage of persons living in households	Depressive symptoms	Measured using the 20-items CES-D scale where respondents classify certain feelings or symptoms in the past week. A score of ≥16 was classified as having high depressive symptomatology	Each 10% increase in percentage Mexican American was associated with a 0.548 point decrease in CES-D score (β=-5.16; 95% CI: -9.47 to -0.85), while each 10% increase in percentage poverty was associated with a 0.763 point increase in CES-D score (β=7.64; 95% CI: 0.40 to 14.88)

						with income below poverty by the census definition			
Powell, W. R. et al., 2020 [89]	United States of America	Cross-sectional study	≥65	2 731	Neighbourhood socioeconomic status/ Neighbourhood deprivation	A neighbourhood economic disadvantage index was calculated based on: per cent of Mexican Americans in census tracts of residence and residence in the border region	Self-rated health	Measured based on the question: "Overall, how would you rate your health—excellent, good, fair, or poor?"	Living in a poorer neighbourhood is associated with having higher odds for rating their health as worst (OR=1.31; 95% CI: 1.11-1.55) than subjects living in more integrated neighbourhoods
Purser, J. L. et al., 2008 [90]	United States of America	Longitudinal study	≥70	1 091	Neighbourhood socioeconomic status/ Neighbourhood deprivation	Based on diverse sources such as: censuses (1931, 1951, 1961, 1971), the Edinburgh Civic Survey 1949, Medical	Mental health	Measured by the Hospital Anxiety and Depression Scale	When analysing the cumulative effect of area-level social deprivation, both men (IRR=1.30; CI=1.05–1.61) and women (IRR=1.22; CI=1.01–1.48) had positive associations with mental health

						Officer of Health reports (1930, 1940, 1947, 1950, 1960) and the National Records of Scotland from 1974 to 1976. Including indicators such as: population density (persons per acre), overcrowding (percentage of population living more than 2 (or 1.5) per room), infant mortality (infant deaths (<1 year) per 1,000 births), tenure (percentage of			

						households renting their accommodation) and amenities (percentage of households without exclusive access to all amenities)			
Ramsay, S. E. et al., 2015 [91]	Scotland	Cross-sectional study	Mean age: 80.3	447	Neighbourhood socioeconomic status/ Neighbourhood deprivation	Assessed with the decade's Decennial Census data through the Area Deprivation Index wich includes measures such as: education level, income, housing, and employment characteristics at the census	Alzheimer Disease	Assessed by being a decedent who donated their brains to Alzheimer Disease Research Center brain bank repository between January 1, 1990, and	The most disadvantaged neighbourhoods had the highest odds for Alzheimer disease neuropathology (OR=2.18; 95% CI: 1.99-2.39)

						block groups level		December 31, 2016	
Reimers, A. & Laflamme, L., 2007 [92]	United States of America	Longitudinal study	≥71	1 726	Neighbourhood socioeconomic status/ Neighbourhood deprivation	Based on: population density; race, age and gender segregation; poverty-related indicators (median family income, per cent of residents of all ages living in poverty, and per cent of residents aged 65 years and over and in poverty); occupational class; and housing quality and/or desirability in census tracts	Chronic inflammation	Assessed with blood samples to ascertain levels of coagulation and inflammatory factors, including IL-6	Communities with greater densities of poor older adults (OR= 1.25; 95% CI: 1.05-1.48) and racially segregated communities (OR= 1.14; 95% CI: 1.04-1.25) had higher odds of elevated IL-6

Ribeiro, A. I. et al., 2015 [93]	United States of America	Case-control study	60–79	3 924	Neighbourhood socioeconomic status/ Neighbourhood deprivation	Measured using the indices of multiple deprivation for England (indicators of: income, employment, health and disability, education, skills and training, barriers to housing and services, living environment and crime), Scotland (similar indicators but also includes geographic access and telecommunications instead of barriers to	Cardiovascular disease mortality	Cardiovascular deaths included in the study were those classified in the International Classification of Diseases, ninth revision codes of 401–459 through the National Health Service Central Register. Non-fatal myocardial	The risk of cardiovascular disease mortality showed a graded increase in more deprived quintiles. For example, those living in poorer neighbourhoods have 43% more odds of dying from cardiovascular disease (OR=1.43; 95% CI: 1.07 to 1.92) than those living in more affluent regions

						housing', 'living environment' and 'crime'), and Wales (indicators of: income, employment, education, health, access to services, housing and environment). For Scotland, the geographical unit used was the 'Data Zones', and the others used super output areas as their geographical unit		infarction and non-fatal stroke were identified from biennial reviews of the general practitioner records.	
Robert, S. A. & Ruel, E., 2006	United Kingdom	Cross-sectional study	≥65	7 748	Neighbourhood socioeconomic	Using parishes as the unit of analysis,	Hip fracture	Measured based on the ninth	Living in an average (women OR=0.73; 95% CI: 0.68-0.78; men

[94]					status/ Neighbourhood deprivation	neighbourhood attributes were categorised into two main factors: "social status" (proportion of people with no more than 9 years of compulsory school or elementary school in the age group 25–64 years, inhabitants per hectare, proportion of families without car, proportion of people living in rented accommodation) and "economic		version of the International Classification of Diseases from the Hospital Discharge Register of the Health Care Board of Stockholm County Council	OR=0.77; 95% CI:0.69-0.85) or low social status parish (women OR=0.67; 95% CI:0.59-0.77; men OR=0.61; 95% CI:0.48-0.76) lower the odds of suffering a hip fracture

						deprivation" (proportion of people who received social welfare sometime during one year in the age group 18–64 years, proportion unemployed in the age group 18–64 years, proportion men on a low income in the age group 20–64 years, proportion single parents with children below the age of 21)			
Rosso, A. L. et al., 2016 [95]	Sweden	Cross-sectional study	≥65	532	Neighbourhood socioeconomic	Measured using the census tract of	Physical activity	Measured with the EPIPorto	Neighbourhood socioeconomic status was not significantly

					status/ Neighbourhood deprivation	residence: socioeconomic status of the census tract of residence (age and education/occupation distribution of its residents, and housing characteristics), population density of the census tract of residence, distance from the residence to the nearest park, distance to the nearest sports space, distance to the nearest non-residential destination, distance to the sea/riverside,		Physical Activity Questionnaire to measure time and intensity of leisure-time physical activity either light (e.g. brisk walking, golfing, snooker), moderate (e.g. walk at moderate pace, dancing, stretching) or vigorous (e.g. running,	associated with leisure-time physical activity

						density of street intersections within 200 m of the residence, density of bus/metropolitan stops within 200 m, average land gradient within 200 m		soccer, basketball)	
Salvatore, M. A. & Grundy, E., 2021 [96]	Portugal	Cross-sectional study	≥60	1095 participants from the Americans' Changing Lives survey and 1615 participants from the National Survey	Segregation	The racial segregation index was measured with the Dissimilarity Index (to measure residential unevenness between two groups) - higher values mean higher levels of	Self-rated health	Assessed with the question: ''How would you rate your health at the present time?'' Response categories were: excellent,	There was no strong evidence that racial segregation was associated with self-reported health among older adults

				of Families and Households survey		segregation using census tracts as their neighbourhoods		very good, good, fair, or poor in the Americans’ Changing Lives survey and with the question ‘‘Compared with other people your age, how would you describe your health?’’ Response categories were: excellent, good, fair,	

								poor, or very poor in the National Survey of Families and Households survey. To compare data, the information was coded as: ACL self-rated health reports as 1=excellent, 2=very good or good, 3=fair, and 4=poor and National Survey of	

								Families and Households survey self-rated health reports as 1=excellent, 2=good, 3=fair, and 4=poor or very poor	
Sarkar, C. et al., 2013 [97]	United States of America	Longitudinal study	≥65	3 241	Neighbourhood socioeconomic status/ Neighbourhood deprivation	Based on the 1990 US Census block groups and includes: median household income, median value of housing units, and percentage of households receiving interest, dividend, or	Cognitive function	Assessed annually using the Digit Symbol Substitution Test (higher scores indicate faster speed of processing) and the Modified Mini-	For the Modified Mini-Mental State Examination Scores, white participants living in the poorest neighbourhoods (mean difference= -0.22; 95% CI: -0.43 to -0.01) faster rates of decline compared to the highest tertile. For the Digit Symbol Substitution Test Scores there was no evidence that neighbourhood socioeconomic status

						net rental income; percentage of adults who completed high school, percentage of adults who completed college; and percentage of persons in managerial or professional specialty occupations		Mental State Examination (higher scores indicate better cognition)	was associated with cognitive function
Schieman, S. et al., 2006 [98]	United States of America	Longitudinal study	≥65	6 643	Neighbourhood socioeconomic status/ Neighbourhood deprivation	Assessed with the index of multiple deprivation, which includes: income, employment, education, health, crime, access to services,	Mental health	Assessed using SF-12 Mental Component Summary score, which includes: physical functioning; role	Living in a more deprived neighbourhood is associated with worse perceived social cohesion (β=-0.05, for both men and women) and worse mental health (men β=-0.04 and women β= -0.03)

						housing, and the physical environment at the level of 2001 Lower-Layer Super Output Areas		limitations due to physical health problems; bodily pain; general health; vitality (energy/fatigue); social functioning; role limitations due to emotional problems; and mental health	
Sheffield, K. M. & Peek, M. K., 2009 [99]	United Kingdom	Cross-sectional study	65–84	687	Neighbourhood socioeconomic status/ Neighbourhood deprivation	Area-level deprivation was assessed with the Welsh index of multiple	Psychological distress	Assessed using the self-completion 30-item General	Reduced odds of psychological distress was associated with greater employment deprivation (OR = 0.96,

						deprivation scores, which includes: income, employment, health, education, housing, access to services and physical environment measured at the level of Lower Super Output Area		Health Questionnaire	95% CI: -0.08 to 0.00; p-value=0.02)
Shih, R. A., et al., 2011 [100]	United Kingdom	Cross-sectional study	≥65	1 136	Neighbourhood socioeconomic status/ Neighbourhood deprivation	Neighbourhood disadvantage was assessed with 2000 census and based on: households below poverty level, overcrowded households,	Anger	It was measured based on an index created with the question: "In the past week, on how many days did	Living in a disadvantaged neighbourhood is positively associated with anger among lower-income elders who feel financially advantaged relative to their neighbours (coefficient=0.300; standard error=0.097), while living in a

						individuals receiving public assistance, and female-headed single-parent households		you": "feel very critical of others," "become easily annoyed or irritated," "argue with someone," "feel angry," and "yell at someone." The response categories were "no days", "1 or 2 days", "3 or 4 days", and "5 or more days". Higher	disadvantaged neighbourhood is positively associated with anger among higher-income elders who feel financially disadvantaged relative to their neighbours (coefficient=0.373; standard error=0.153)

								scores indicate a greater frequency of angry feelings and anger-related behaviours	
Sisco, S. M. & Marsiske, M., 2012 [101]	United States of America	Longitudinal study	≥65	1 876	Neighbourhood socioeconomic status/ Neighbourhood deprivation	Measured based on: education, occupational class, poverty, housing, and income of the US census tracts	Cognitive function	Assessed using the English and Spanish versions of the Mini-Mental State Examination	Being a Mexican-American decreases the odds of incident cognitive decline (OR=0.33; 95% CI: 0.12-0.88), and living in more disadvantaged neighbourhoods increases the odds of incident cognitive decline (OR=1.75; 95% CI: 1.07-2.88) compared to more affluent neighbourhoods
Smith, R. J. et al., 2018 [102]	United States of America	Cross-sectional study	≥65	6137	Neighbourhood socioeconomic status/	Assessed using an index of 6 census tract variables:	Cognitive function	Assessed with the Modified Mini-	Elderly living in higher deprived neighbourhoods have significantly lower

					Neighbourhood deprivation	percentage of adults older than 25 years with less than a high school education, percentage of male unemployment, percentage of households with income levels below the poverty line, percentage of households receiving public assistance, percentage of female-headed households with children, and median household income, at the		Mental State Examination	cognitive functioning (B =0.0028; standard error: 0.0017)

						census tract level			
Stroope, S. et al., 2015 [103]	United States of America	Cross-sectional study	65-94	2 521	Neighbourhood socioeconomic status/ Neighbourhood deprivation	Assessed calculating an index that includes measures such as: income, education, and occupation at the census tracts level	Cognitive function	Measured based on: memory, inductive reasoning, processing speed, everyday cognition, and vocabulary	There was no strong evidence that neighbourhoods socioeconomic status was associated with cognitive ability
Stroope, S. et al., 2017 [104]	United States of America	Quasi-experimental study	≥65	6 810	Gentrification	It was classified as gentrifying if in the year 2000 a neighbourhood had an average household income less than the 40th percentile of the metropolitan	Self-rated health	Self-rated health was assessed using the question: "Would you say that in general your health is…" where 0 = poor, 1 =	Economically vulnerable individuals living in gentrifying neighbourhoods had higher scores on their self-rated health (coefficient=1.81; 95% CI: 1.10-2.51) compared to economically vulnerable individuals living in low-income neighbourhoods. Higher-income individuals living in a

						area and resided in a primary city or inner-ring suburb, and there was an increase in: (a) median household income, (b) per cent of college-educated residents, (c) median owner-occupied housing values, and (d) median rent; the neighbourhood would be classified as low-income neighbourhood if average household income less		fair, 2 = good, 3 = very good, 4 = excellent. Mental health was assessed by examining depression and anxiety symptoms using the Patient Health Questionnaire-4 that sees if respondents had little interest, felt down, felt nervous, or were	gentrifying neighbourhood had higher scores on their mental health, which indicates greater levels of depression and anxiety (coefficient=3.62; 95% CI: 2.84-4.41) compared to higher-income individuals living in low-income neighbourhoods. Economically vulnerable and higher-income individuals living in gentrifying neighbourhoods also had higher scores on their mental health (coefficient=4.79; 95% CI: 2.79-6.78 and coefficient=3.64; 95% CI: 2.87-4.41, respectively) compared to their peers living in moderate-to-high income neighbourhoods

						than the 40th percentile of the metropolitan area in 2000 and did not gentrify by 2010; other situations that did not fit the other two conditions resulted in the classification of a moderate-to-high income neighbourhood.		unable to stop worrying over the past month (higher score indicates greater levels of depression and anxiety)	
Subramanian, S. V. et al., 2006 [105]	United States of America	Cross-sectional study	≥75	350 men	Neighbourhood socioeconomic status/ Neighbourhood deprivation	Assessed at the census tracts level with an index of neighbourhood economic disadvantage based on: per cent in	Drinking problem	Measured by using the 4-item Cut, Annoyed, Guilty, Eye Opener instrument for	No statistically significant association was observed between neighbourhood economic disadvantage index and having a drinking problem

						poverty, per cent below 50 % of the poverty line, Hispanic per cent in poverty, male unemployment rate, female unemployment rate, per cent with public assistance income, and per cent with investment income		detecting alcohol abuse amongst Latinos	
Timmermans, E. et al., 2020 [106]	United States of America	Cross-sectional study	≥75	1 780	Neighbourhood socioeconomic status/ Neighbourhood deprivation	Measured with an index of neighbourhood economic disadvantage which includes: percentage in poverty, percentage below 50% of	Self-rated health	"Overall, how would you rate your health: excellent, good, fair or poor?"	Being economically deprived is associated with reporting worst self-rated health (coefficient=0.25; standard error=1.12; OR=1.28)

						the poverty line, Hispanic percentage in poverty, unemploymen t rate, percentage on public assistance and percentage with investment income at census tract-level			
Van Dyck, D. et al., 2020 [107]	United States of America	Cross-sectional study	≥65	1 926	Neighbourhoo d socioeconomic status/ Neighbourhoo d deprivation	Assessed with the 1980 Census Summary Tape Files and based on the meaures of: proportion of households within a census tract who lived below the	Self-rated Health	Based on the question: ''How would you rate your health at the present time? Excellent, good, fair,	Living in poorer neighbourhoods is associated with reporting poorer self-rated health (OR=1.09; 95% CI: 1.0-1.17), whereas having residential stability (OR=0.90; 95% CI: 0.84-0.96) and living in a neighbourhood with more elderly people (OR=0.82; 95% CI: 0.72-

						official poverty threshold, median income of the census tract, and proportion of households within a census tract who had incomes of more than $75,000 per year, proportion of people who had lived in the same house for the past 5 years, proportion of people aged 65 and older and proportion African Americans. Measures of		poor, or bad.''	0.94) is associated with better self-rated health

						service density were assessed with the yellow pages			
Vogt, S. et al., 2015 [108]	United States of America	Cross-sectional study	63–98	1 959	Neighbourhood socioeconomic status/ Neighbourhood deprivation	Assessed using Statistics Netherlands to collect information on social neighbourhood characteristics: average income (retrieved at the neighbourhood-level), percentage of social security beneficiaries (retrieved at the neighbourhood-level), educational level (retrieved	Loneliness	Assessed using the De Jong Gierveld Loneliness Scale	There was no strong evidence that neighbourhood characteristics were associated with loneliness

						at the four-digit postal code area-level), crime (retrieved at the neighbourhood-level), and percentage of unoccupied dwellings (neighbourhood-level). The four-digit postal code areas correspond to an average area size of 8.3 km2 and include, on average, 1870 households. The neighbourhoods correspond to an average area size of 3.1			

						km2 and include, on average, 630 households			
Wagner, K. J. et al., 2016 [109]	Netherlands	Cross-sectional study	≥65	Belgium sample: 427 and China sample: 402	Neighbourhood socioeconomic status/ Neighbourhood deprivation	Assessed using census-based median annual household income data	Body Mass Index	Measured based on a calculation using objectively-assessed weight and height where body mass index was categorised as: ≤ 24.9 kg/m2 for normal/underweight and ≥ 25 kg/m2 for overweight/obese	Living in a more affluent neighbourhood was associated with having a lower BMI (coefficient= -0.567; 95% CI: -1.114 to -0.0024)
Walters, K. et al., 2004 [110]	Belgium and China	Cross-sectional study	≥65	1 711	Neighbourhood socioeconomic status/	Regions were used as a proxy for the neighbourhoo	Healthy ageing	Conceptualise based on the constructs	There was no strong evidence that neighbourhood deprivation was

					Neighbourhood deprivation	d. Regional deprivation was classified into two categories: low and high according to their proportion of social welfare recipients		physical functioning (self-assessment of physical constitution and disability) and health-related quality of life (using the European Quality of Life questionnaire)	associated with healthy ageing
Wee, L. E. et al., 2012 [111]	Germany	Cross-sectional study	≥60	1 705	Neighbourhood socioeconomic status/ Neighbourhood deprivation	Assessed by using the mean years of schooling of the head of the household in each census tract	Hypertension	Hypertension was defined as having a systolic blood pressure ≥ 140	Living in low-education neighbourhoods increases the odds of having hypertension (OR=1.76; 95% CI: 1.23-2.51)

								mmHg, diastolic blood pressure ≥ 90 mmHg or both. It was also considered hypertension if the individual reported using antihypertensive medication	
Wee, L. E. et al., 2014 [112]	Brazil	Cross-sectional study	≥75	13 349	Neighbourhood socioeconomic status/ Neighbourhood deprivation	Neighbourhood deprivation was assessed with: levels of unemployment, overcrowding, non–car ownership, and social	Anxiety and depression	Anxiety was assessed with anxiety subscale of the General Health Questionn	There was no strong evidence that deprived neighbourhoods were associated with anxiety or depression

						class at the enumeration district level		aire. Depression was assessed with the Geriatric Depression Scale	
Weil, J., 2019 [113]	United Kingdom	Cross-sectional study	≥60	558	Neighbourhood socioeconomic status/ Neighbourhood deprivation	Assessed using: percentage of families that are composed of husband and wife; percentage of households dependent on public assistance; percentage of households which have an annual income above the median; percentage of adults without	Cognitive impairment	Cognitive impairment was defined when the Mini-Mental State Examination score was < 24	Living in poorer neighbourhoods was associated with higher odds of cognitive impairment (OR public rental flat neighbourhood =5.13; 95% CI = 1.98–13.34 and OR higher neighbourhood deprivation = 3.83; 95% CI: 1.65-8.85)

						secondary education; percentage of housing units that are rentals, and adult unemployment rate at the block level			
Wight, R. G. et al., 2006 [114]	Singapore	Cross-sectional study	≥60	559	Neighbourhood socioeconomic status/ Neighbourhood deprivation	Assessed using: whether the resident was staying in a block predominantly composed of public rental housing and also the percentage of husband-wife families; the percentage of households on public assistance; the percentage of	Depression	Assessed using 15-item Geriatric Depression Scale	Living in predominantly public rental housing neighbourhoods (less residential stability) was positively associated with depression (OR= 1.68, 95%CI = 1.02–2.84)

						households that have annual income above the median; the percentage of adults without secondary education; the percentage of housing units that are rentals; and adult unemployment rate at the block level			
Wight, R. G. et al., 2008 [115]	Singapore	Qualitative study	≥65	29	Gentrification	Defined by Ruth Glass's and earlier groups' conceptualization of the process as when wealthier classes move into a	Ageing in place	Narratives about the meaning of place and gentrification	Older adults pointed out struggles of identity and loss of place, highlighted the insider-outsider dichotomy, feelings of displacement, the adaptations made to the neighbourhood, and also the benefits of neighbourhood change

						working-class community, purchase homes and businesses, and change neighbourhood infrastructure and verified in a neighbourhood in Queens, New York City			
Wight, R. G. et al., 2009 [116]	United States of America	Cross-sectional study	≥70	3 442	Neighbourhood socioeconomic status/ Neighbourhood deprivation	Assessed with the neighbourhood education as the proportion of residents aged 25 years or older without a high school degree at the census tract level	Cognitive function	Assessed using the Telephone Interview for Cognitive Status	Elderly living in low-education areas is associated with having low cognitive function than those that had attained at least a high school education (coefficient=-6.29; standard error=1.80)

Wight, R. G. et al., 2010 [117]	United States of America	Cross-sectional study	≥70	3 442	Neighbourhood socioeconomic status/ Neighbourhood deprivation	Assessed using 1990 U.S. Census tract data on: residents aged 25 or older without a high school degrees; households receiving public assistance income; residents living below the poverty level; and residents aged 16 or older who are unemployed	Self-reported physician-diagnosed cardiovascular disease, functional status, and self-rated health	Self-reported physician-diagnosed cardiovascular disease was assessed through three questions: if a doctor ever told them, they had a heart problem, a stroke or diabetes. Functional status was assessed with reported difficulties with both basic	Neighbourhood socioeconomic disadvantage was significantly associated with self-reporting poor health (OR=1.12; 95% CI: 1.01-1.26)

								activities of daily living and instrumental activities of daily living. And self-rated health was assessed with the question: "Would you say your health is excellent, very good, good, fair, or poor?”	
Wörn, J. et al., 2017 [118]	United States of America	Longitudinal study	≥70	3 442	Neighbourhood socioeconomic status/ Neighbourhood deprivation	Assessed with 1990 U.S. Census data on: the proportion of residents aged	Depressive symptoms	Assessed with the eight-item Center for Epidemiologic	There was no strong evidence that deprived neighbourhoods were associated with depressive symptoms

						25 years or older without a high school degree, households receiving public assistance income, residents living below the poverty level, and residents aged 16 years or older who are unemployed, the proportion of households with incomes of $50,000 or more, proportion of residents who are African American and proportion of Hispanic		Studies Depressio n scale	

						residents, residential stability is the proportion of people aged 5 years or older who lived in the same house for the past 5 years, and proportion of residents who are over the age of 65 years			
Wu, Y. T. et al., 2015 [119]	United States of America	Longitudinal study	≥70	3 442	Neighbourhood socioeconomic status/ Neighbourhood deprivation	Assessed with 1990 U.S. Census data on: a socioeconomic disadvantage principal component (proportion of the following: residents aged 25 or older without a high	Mortality	Assessed with Assets and Health Dynamics Among the Oldest Old/Health and Retirement Study Tracker File where	Living in more affluent neighbourhoods lower the odds of dying (OR= 0.37; 95% CI: 0.14-0.97)

						school degree; households receiving public assistance income; residents living below the poverty level; and residents aged 16 or older who are unemployed), which is consistent with other studies that seek to globally capture the concept of neighbourhood SES, proportion of households with incomes of US$50,000 or more,		the deaths are verified through the National Death Index	

						proportion of residents who are African American, proportion of residents who are Hispanic, proportion of people aged 5 or older who lived in the same house for the past 5 years, proportion of persons who are above the age of 65 years at the census tract level			
Yan, T. et al., 2013 [120]	United States of America	Longitudinal study	65-88	985	Neighbourhood socioeconomic status/ Neighbourhood deprivation	Assessed using Statistics Netherlands and neighbourhood socioeconomic status was	Cognitive function	Assessed using the Mini-Mental State Examination as a measure	There was no strong evidence that neighbourhoods with lower socioeconomic status or lower degrees of urbanity were associated with faster cognitive decline

						operationalized as the average net income per inhabitant in the neighbourhood in the previous year (1994). Neighbourhoods were defined by their historical or built characteristics		of cognition, including orientation in time and space, registration, attention, recall, language and visuospatial abilities, with higher values indicating better cognitive functioning	
Yao, L. & Robert, S. A., 2008 [121]	Netherlands	Cross-sectional study	≥65	2 424	Neighbourhood socioeconomic status/ Neighbourhood deprivation	Assessed through the UK Government Neighbourhood Statistics and	Cognitive function	Assessed using the Mini-Mental State Examinati	Area deprivation was not significantly associated with cognitive impairment and dementia after

						was based on the English Index of Multiple Deprivation 2004, which includes: income, employment, education and training, health and disability, barriers to housing and services, the living environment and crime at the Lower-layer Super Output Areas level		on, where cognitive impairme nt was defined as a score of ≤25	accounting for individual-level factors
Yao, L. & Robert, S. A., 2011 [122]	United Kingdo m	Longitudinal study	≥65	3 834	Neighbourhoo d socioeconomic status/	Assessed with 1990 US Census data based on measures such	Stroke	Assessed with reviews at annual visits,	Living in the poorest neighbourhood was associated with a higher risk of suffering a stroke

					Neighbourhood deprivation	as: wealth/income, education, and occupation at the census tract level		interim telephone contacts, notification of events by participants and review of Medicare hospitalisation data	(HR=1.31; 95% CI: 1.00-1.71), among whites

References

1. Alaazi, D.A., et al., *Quality of life of older adults in two contrasting neighbourhoods in Accra, Ghana.* Social Science and Medicine, 2021. **270**.
2. Almeida, O.P., et al., *Socioeconomic disadvantage increases risk of prevalent and persistent depression in later life.* J Affect Disord, 2012. **138**(3): p. 322-31.
3. Amuzu, A., et al., *Influence of area and individual lifecourse deprivation on health behaviours: findings from the British Women's Heart and Health Study.* European journal of cardiovascular prevention and rehabilitation, 2009. **16**(2): p. 169-173.
4. Aneshensel, C.S., et al., *Urban neighborhoods and depressive symptoms among older adults.* J Gerontol B Psychol Sci Soc Sci, 2007. **62**(1): p. S52-9.
5. Annear, M.J., G. Cushman, and B. Gidlow, *Leisure time physical activity differences among older adults from diverse socioeconomic neighborhoods.* Health Place, 2009. **15**(2): p. 482-490.
6. Annear, M.J., B. Gidlow, and G. Cushman, *Neighbourhood deprivation and older adults' preferences for and perceptions of active leisure participation.* Annals of Leisure Research, 2009. **12**(2): p. 96-128.
7. Araújo, C.A.H., et al., *Built environment, contextual income, and obesity in older adults: evidence from a population-based study.* Cad Saude Publica, 2018. **34**(5): p. e00060217.

8. Auchincloss, A.H., J.F. Van Nostrand, and D. Ronsaville, *Access to health care for older persons in the United States: personal, structural, and neighborhood characteristics.* J Aging Health, 2001. **13**(3): p. 329-54.
9. Balamurugan, A., et al., *The neighborhood where you live is a risk factor for stroke.* Circulation: Cardiovascular Quality and Outcomes, 2013. **6**(6): p. 668-673.
10. Basta, N.E., et al., *Community-level socio-economic status and cognitive and functional impairment in the older population.* European Journal of Public Health, 2007. **18**(1): p. 48-54.
11. Beard, J.R., et al., *Neighborhood characteristics and disability in older adults.* J Gerontol B Psychol Sci Soc Sci, 2009. **64**(2): p. 252-7.
12. Beere, P., S. Keeling, and H. Jamieson, *Ageing, loneliness, and the geographic distribution of New Zealand's interRAI-HC cohort.* Soc Sci Med, 2019. **227**: p. 84-92.
13. Behanova, M., et al., *The effect of neighbourhood unemployment on health-risk behaviours in elderly differs between Slovak and Dutch cities.* Eur J Public Health, 2015. **25**(1): p. 108-14.
14. Behanova, M., et al., *Elderly from lower socioeconomic groups are more vulnerable to mental health problems, but area deprivation does not contribute: a comparison between Slovak and Dutch cities.* Eur J Public Health, 2017. **27**(suppl_2): p. 80-85.
15. Bhardwaj, R., et al., *Environmental Correlates of Reaching a Centenarian Age: Analysis of 144,665 Deaths in Washington State for 2011-2015.* Int J Environ Res Public Health, 2020. **17**(8).
16. Bolstad, C.J., et al., *Neighborhood Disadvantage Is Associated with Depressive Symptoms but Not Depression Diagnosis in Older Adults.* Int J Environ Res Public Health, 2020. **17**(16).
17. Bowling, A. and M. Stafford, *How do objective and subjective assessments of neighbourhood influence social and physical functioning in older age? Findings from a British survey of ageing.* Soc Sci Med, 2007. **64**(12): p. 2533-49.
18. Breeze, E., et al., *Area deprivation, social class, and quality of life among people aged 75 years and over in Britain.* Int J Epidemiol, 2005. **34**(2): p. 276-83.
19. Brenner, A.B. and P.J. Clarke, *Difficulty and independence in shopping among older Americans: more than just leaving the house.* Disabil Rehabil, 2019. **41**(2): p. 191-200.
20. Brown, A.F., et al., *Neighborhood disadvantage and ischemic stroke: the Cardiovascular Health Study (CHS).* Stroke, 2011. **42**(12): p. 3363-8.
21. Brown, S.C., et al., *Health Disparities in the Relationship of Neighborhood Greenness to Mental Health Outcomes in 249,405 US Medicare Beneficiaries.* International Journal of Environmental Research and Public Health, 2018. **15**(3).
22. Buffel, T. and C. Phillipson, *Ageing in a Gentrifying Neighbourhood: Experiences of Community Change in Later Life.* Sociology-the Journal of the British Sociological Association, 2019. **53**(6): p. 987-1004.

23. Buffel, T., C. Phillipson, and T. Scharf, *Experiences of neighbourhood exclusion and inclusion among older people living in deprived inner-city areas in Belgium and England.* Ageing & Society, 2013. **33**: p. 89-109.
24. Burns, V.F., J.P. Lavoie, and D. Rose, *Revisiting the role of neighbourhood change in social exclusion and inclusion of older people.* J Aging Res, 2012. **2012**: p. 148287.
25. Buys, D.R., et al., *Association between neighborhood disadvantage and hypertension prevalence, awareness, treatment, and control in older adults: results from the University of Alabama at Birmingham Study of Aging.* Am J Public Health, 2015. **105**(6): p. 1181-8.
26. Cadar, D., et al., *Individual and Area-Based Socioeconomic Factors Associated With Dementia Incidence in England: Evidence From a 12-Year Follow-up in the English Longitudinal Study of Ageing.* JAMA Psychiatry, 2018. **75**(7): p. 723-732.
27. Cagney, K.A., et al., *The onset of depression during the great recession: foreclosure and older adult mental health.* Am J Public Health, 2014. **104**(3): p. 498-505.
28. Casanova, R., et al., *Investigating predictors of cognitive decline using machine learning.* Journals of Gerontology - Series B Psychological Sciences and Social Sciences, 2020. **75**(4): p. 733-742.
29. Cerin, E., et al., *Socioeconomic status, neighborhood characteristics, and walking within the neighborhood among older Hong Kong Chinese.* J Aging Health, 2013. **25**(8): p. 1425-44.
30. Chaix, B., M. Rosvall, and J. Merlo, *Assessment of the magnitude of geographical variations and socioeconomic contextual effects on ischaemic heart disease mortality: a multilevel survival analysis of a large Swedish cohort.* J Epidemiol Community Health, 2007. **61**(4): p. 349-55.
31. Chamberlain, A.M., et al., *Neighborhood socioeconomic disadvantage is associated with multimorbidity in a geographically-defined community.* BMC Public Health, 2020. **20**(1).
32. Corriere, M.D., et al., *The association of neighborhood characteristics with obesity and metabolic conditions in older women.* J Nutr Health Aging, 2014. **18**(9): p. 792-8.
33. Danielewicz, A.L., et al., *Is cognitive decline in the elderly associated with contextual income? Results of a population-based study in southern Brazil.* Cad Saude Publica, 2016. **32**(5): p. e00112715.
34. Deng, G. and L. Mao, *Spatially Explicit Age Segregation Index and Self-Rated Health of Older Adults in US Cities.* Isprs International Journal of Geo-Information, 2018. **7**(9).
35. Diez Roux, A.V., et al., *Neighbourhood environments and mortality in an elderly cohort: results from the cardiovascular health study.* J Epidemiol Community Health, 2004. **58**(11): p. 917-23.
36. Domínguez-Parraga, L., *The effects of gentrification on the elderly: A case study in the city of Cáceres.* Social Sciences, 2020. **9**(9).

37. Espino, D.V., et al., *Ethnic differences in Mini-Mental State Examination (MMSE) scores: Where you live makes a difference.* Journal of the American Geriatrics Society, 2001. **49**(5): p. 538-548.
38. Espinoza, S.E. and H.P. Hazuda, *Frailty prevalence and neighborhood residence in older Mexican Americans: the San Antonio longitudinal study of aging.* J Am Geriatr Soc, 2015. **63**(1): p. 106-11.
39. Everson-Rose, S.A., et al., *Neighborhood socioeconomic conditions are associated with psychosocial functioning in older black and white adults.* Health Place, 2011. **17**(3): p. 793-800.
40. Fernández-Blázquez, M.A., et al., *Impact of individual and neighborhood dimensions of socioeconomic status on the prevalence of mild cognitive impairment over seven-year follow-up.* Aging Ment Health, 2020: p. 1-10.
41. Fox, K.R., et al., *Neighbourhood deprivation and physical activity in UK older adults.* Health Place, 2011. **17**(2): p. 633-40.
42. Franse, C.B., et al., *Socioeconomic inequalities in frailty and frailty components among community-dwelling older citizens.* PLoS One, 2017. **12**(11): p. e0187946.
43. Gale, C.R., et al., *Neighbourhood environment and positive mental health in older people: the Hertfordshire Cohort Study.* Health Place, 2011. **17**(4): p. 867-74.
44. Garcia, L., et al., *The Impact of Neighborhood Socioeconomic Position on Prevalence of Diabetes and Prediabetes in Older Latinos: The Sacramento Area Latino Study on Aging.* Hisp Health Care Int, 2015. **13**(2): p. 77-85.
45. Garcia, L., et al., *Influence of neighbourhood socioeconomic position on the transition to type II diabetes in older Mexican Americans: the Sacramento Area Longitudinal Study on Aging.* BMJ Open, 2016. **6**(8): p. e010905.
46. Giehl, M.C.G., et al., *Built Environment and Walking Behavior Among Brazilian Older Adults: A Population-Based Study.* J Phys Act Health, 2016. **13**(6): p. 617-24.
47. Guo, Y., et al., *Neighborhood environment and cognitive function in older adults: A multilevel analysis in Hong Kong.* Health Place, 2019. **58**: p. 102146.
48. Guo, Y., et al., *Association of neighbourhood social and physical attributes with depression in older adults in Hong Kong: a multilevel analysis.* J Epidemiol Community Health, 2019. **74**(2): p. 120-129.
49. Hannon, L., 3rd, P. Sawyer, and R.M. Allman, *Housing, the Neighborhood Environment, and Physical Activity among Older African Americans.* J Health Dispar Res Pract, 2012. **5**(3): p. 27-41.
50. Hawkesworth, S., et al., *Investigating associations between the built environment and physical activity among older people in 20 UK towns.* J Epidemiol Community Health, 2018. **72**(2): p. 121-131.

51. Hazzouri, A.Z.A., et al., *Neighborhood socioeconomic context and cognitive decline among older Mexican Americans: results from the Sacramento Area Latino Study on Aging.* Am J Epidemiol, 2011. **174**(4): p. 423-31.
52. Huang, Y., P. Meyer, and L. Jin, *Neighborhood socioeconomic characteristics, healthcare spatial access, and emergency department visits for ambulatory care sensitive conditions for elderly.* Prev Med Rep, 2018. **12**: p. 101-105.
53. Hybels, C.F., et al., *Sociodemographic characteristics of the neighborhood and depressive symptoms in older adults: using multilevel modeling in geriatric psychiatry.* Am J Geriatr Psychiatry, 2006. **14**(6): p. 498-506.
54. Joshi, S., et al., *Pathways from neighborhood poverty to depression among older adults.* Health Place, 2017. **43**: p. 138-143.
55. Jung, D., et al., *Linking Neighborhood Context and Health in Community-Dwelling Older Adults in the Medicare Advantage Program.* J Am Geriatr Soc, 2018. **66**(6): p. 1158-1164.
56. Kelley-Moore, J.A., et al., *Do Local Social Hierarchies Matter for Mental Health? A Study of Neighborhood Social Status and Depressive Symptoms in Older Adults.* J Gerontol B Psychol Sci Soc Sci, 2016. **71**(2): p. 369-77.
57. Kim, G.H., et al., *Effect of Individual and District-level Socioeconomic Disparities on Cognitive Decline in Community-dwelling Elderly in Seoul.* J Korean Med Sci, 2017. **32**(9): p. 1508-1515.
58. Ko, J.E., et al., *Neighborhood effects on the self-rated health of older adults from four racial/ethnic groups.* Soc Work Public Health, 2014. **29**(2): p. 89-99.
59. Kubzansky, L.D., et al., *Neighborhood contextual influences on depressive symptoms in the elderly.* American Journal of Epidemiology, 2005. **162**(3): p. 253-260.
60. Kwag, K.H., et al., *Neighborhood Effects on Physical and Mental Health: A Study of Korean American Older Adults.* Asian Am J Psychol, 2011. **2**(2): p. 91-100.
61. Lager, D., B. Van Hoven, and P.P.P. Huigen, *Dealing with change in old age: Negotiating working-class belonging in a neighbourhood in the process of urban renewal in the Netherlands.* Geoforum, 2013. **50**: p. 54-61.
62. Lang, I.A., et al., *Neighborhood deprivation, individual socioeconomic status, and cognitive function in older people: analyses from the English Longitudinal Study of Ageing.* J Am Geriatr Soc, 2008. **56**(2): p. 191-8.
63. Lang, I.A., et al., *Neighbourhood deprivation and incident mobility disability in older adults.* Age Ageing, 2008. **37**(4): p. 403-10.
64. Lang, I.A., et al., *Neighbourhood deprivation and dental service use: a cross-sectional analysis of older people in England.* J Public Health (Oxf), 2008. **30**(4): p. 472-8.
65. Lang, I.A., et al., *Neighborhood deprivation, individual socioeconomic status, and frailty in older adults.* J Am Geriatr Soc, 2009. **57**(10): p. 1776-80.

66. Lawlor, D.A., et al., *Life-course socioeconomic position, area deprivation, and coronary heart disease: findings from the British Women's Heart and Health Study.* American journal of public health, 2005. **95**(1): p. 91-97.
67. Letellier, N., et al., *Sex-specific association between neighborhood characteristics and dementia: The Three-City cohort.* Alzheimers Dement, 2017. **14**(4): p. 473-482.
68. Letellier, N., et al., *Influence of activity space on the association between neighborhood characteristics and dementia risk: results from the 3-City study cohort.* BMC Geriatr, 2019. **19**(1): p. 4.
69. Li, W., et al., *Utilitarian walking, neighborhood environment, and risk of outdoor falls among older adults.* Am J Public Health, 2014. **104**(9): p. e30-7.
70. Lo, A.X., et al., *Neighborhood Disadvantage and Life-Space Mobility Are Associated with Incident Falls in Community-Dwelling Older Adults.* J Am Geriatr Soc, 2016. **64**(11): p. 2218-2225.
71. Lönn, S.L., et al., *Accumulated neighbourhood deprivation and coronary heart disease: a nationwide cohort study from Sweden.* BMJ Open, 2019. **9**(9): p. e029248.
72. Meijer, M., et al., *Population density, socioeconomic environment and all-cause mortality: a multilevel survival analysis of 2.7 million individuals in Denmark.* Health Place, 2012. **18**(2): p. 391-9.
73. Menec, V.H., et al., *Does the relationship between neighborhood socioeconomic status and health outcomes persist into very old age? A population-based study.* J Aging Health, 2010. **22**(1): p. 27-47.
74. Merkin, S.S., et al., *Individual and neighborhood socioeconomic status and progressive chronic kidney disease in an elderly population: The Cardiovascular Health Study.* Soc Sci Med, 2007. **65**(4): p. 809-21.
75. Meyer, O.L., et al., *Neighborhood Predictors of Cognitive Training Outcomes and Trajectories in ACTIVE.* Res Aging, 2017. **39**(3): p. 443-467.
76. Miao, J., X. Wu, and X. Sun, *Neighborhood, social cohesion, and the Elderly's depression in Shanghai.* Soc Sci Med, 2019. **229**: p. 134-143.
77. Michael, Y.L., et al., *Does change in the neighborhood environment prevent obesity in older women?* Soc Sci Med, 2014. **102**: p. 129-37.
78. Mooney, S.J., et al., *Contextual Correlates of Physical Activity among Older Adults: A Neighborhood Environment-Wide Association Study (NE-WAS).* Cancer Epidemiol Biomarkers Prev, 2017. **26**(4): p. 495-504.
79. Moser, A., et al., *What does your neighbourhood say about you? A study of life expectancy in 1.3 million Swiss neighbourhoods.* Journal of Epidemiology and Community Health, 2014. **68**(12): p. 1125-1132.
80. Mõttus, R., et al., *'On the street where you live': Neighbourhood deprivation and quality of life among community-dwelling older people in Edinburgh, Scotland.* Soc Sci Med, 2012. **74**(9): p. 1368-74.
81. Nguyen, H.V., *Keeping Up with the Joneses: Neighbourhood Wealth and Hypertension.* Journal of Happiness Studies, 2016. **17**(3): p. 1255-1271.

82. Nicklett, E.J., et al., *Neighborhood socioeconomic status is associated with serum carotenoid concentrations in older, community-dwelling women.* J Nutr, 2011. **141**(2): p. 284-9.
83. Nordstrom, C.K., et al., *The association of personal and neighborhood socioeconomic indicators with subclinical cardiovascular disease in an elderly cohort. The cardiovascular health study.* Soc Sci Med, 2004. **59**(10): p. 2139-47.
84. Nordstrom, C.K., et al., *Socioeconomic position and incident mobility impairment in the Cardiovascular Health Study.* BMC Geriatr, 2007. **7**: p. 11.
85. Omariba, W.R., *Neighbourhood characteristics, individual attributes and self-rated health among older Canadians.* Health and Place, 2010. **16**(5): p. 986-995.
86. Ostir, G.V., et al., *Neighbourhood composition and depressive symptoms among older Mexican Americans.* J Epidemiol Community Health, 2003. **57**(12): p. 987-92.
87. Patel, K.V., et al., *Neighborhood context and self-rated health in older Mexican Americans.* Annals of Epidemiology, 2003. **13**(9): p. 620-628.
88. Pearce, J., et al., *Life course of place: A longitudinal study of mental health and place.* Transactions of the Institute of British Geographers, 2018. **43**(4): p. 555-572.
89. Powell, W.R., et al., *Association of Neighborhood-Level Disadvantage With Alzheimer Disease Neuropathology.* JAMA network open, 2020. **3**(6): p. e207559.
90. Purser, J.L., et al., *Geographical segregation and IL-6: a marker of chronic inflammation in older adults.* Biomark Med, 2008. **2**(4): p. 335-348.
91. Ramsay, S.E., et al., *The influence of neighbourhood-level socioeconomic deprivation on cardiovascular disease mortality in older age: longitudinal multilevel analyses from a cohort of older British men.* J Epidemiol Community Health, 2015. **69**(12): p. 1224-31.
92. Reimers, A. and L. Laflamme, *Hip fractures among the elderly: personal and contextual social factors that matter.* J Trauma, 2007. **62**(2): p. 365-9.
93. Ribeiro, A.I., et al., *Distance to parks and non-residential destinations influences physical activity of older people, but crime doesn't: a cross-sectional study in a southern European city.* BMC Public Health, 2015. **15**: p. 593.
94. Robert, S.A. and E. Ruel, *Racial segregation and health disparities between Black and White older adults.* J Gerontol B Psychol Sci Soc Sci, 2006. **61**(4): p. S203-11.
95. Rosso, A.L., et al., *Neighborhood Socioeconomic Status and Cognitive Function in Late Life.* Am J Epidemiol, 2016. **183**(12): p. 1088-97.
96. Salvatore, M.A. and E. Grundy, *Area deprivation, perceived neighbourhood cohesion and mental health at older ages: A cross lagged analysis of UK longitudinal data.* Health Place, 2021. **67**: p. 102470.

97. Sarkar, C., J. Gallacher, and C. Webster, *Urban built environment configuration and psychological distress in older men: Results from the Caerphilly study.* BMC Public Health, 2013. **13**(1).
98. Schieman, S., L.I. Pearlin, and S.C. Meersman, *Neighborhood disadvantage and anger among older adults: social comparisons as effect modifiers.* J Health Soc Behav, 2006. **47**(2): p. 156-72.
99. Sheffield, K.M. and M.K. Peek, *Neighborhood context and cognitive decline in older Mexican Americans: results from the Hispanic Established Populations for Epidemiologic Studies of the Elderly.* Am J Epidemiol, 2009. **169**(9): p. 1092-101.
100. Shih, R.A., et al., *Neighborhood socioeconomic status and cognitive function in women.* American journal of public health, 2011. **101**(9): p. 1721-1728.
101. Sisco, S.M. and M. Marsiske, *Neighborhood Influences on Late Life Cognition in the ACTIVE Study.* J Aging Res, 2012. **2012**: p. 435826.
102. Smith, R.J., A.J. Lehning, and K. Kim, *Aging in Place in Gentrifying Neighborhoods: Implications for Physical and Mental Health.* Gerontologist, 2018. **58**(1): p. 26-35.
103. Stroope, S., et al., *Neighborhood Ethnic Composition and Problem Drinking Among Older Mexican American Men: Results from the Hispanic Established Populations for the Epidemiologic Study of the Elderly.* Journal of Immigrant and Minority Health, 2015. **17**(4): p. 1055-1060.
104. Stroope, S., et al., *Neighborhood perception and self-rated health among Mexican American older adults.* Geriatr Gerontol Int, 2017. **17**(12): p. 2559-2564.
105. Subramanian, S.V., et al., *Neighborhood effects on the self-rated health of elders: uncovering the relative importance of structural and service-related neighborhood environments.* J Gerontol B Psychol Sci Soc Sci, 2006. **61**(3): p. S153-60.
106. Timmermans, E., et al., *Social and physical neighbourhood characteristics and loneliness among older adults: results from the MINDMAP project.* J Epidemiol Community Health, 2020.
107. Van Dyck, D., et al., *Main and interacting effects of physical activity and sedentary time on older adults' BMI: The moderating roles of socio-demographic and environmental attributes.* PLoS One, 2020. **15**(7): p. e0235833.
108. Vogt, S., et al., *Neighborhood and healthy aging in a German city: distances to green space and senior service centers and their associations with physical constitution, disability, and health-related quality of life.* Eur J Ageing, 2015. **12**(4): p. 273-283.
109. Wagner, K.J., et al., *Effects of neighborhood socioeconomic status on blood pressure in older adults.* Rev Saude Publica, 2016. **50**: p. 78.
110. Walters, K., et al., *Local area deprivation and urban-rural differences in anxiety and depression among people older than 75 years in Britain.* Am J Public Health, 2004. **94**(10): p. 1768-74.
111. Wee, L.E., et al., *Individual and Area Level Socioeconomic Status and Its Association with Cognitive Function and Cognitive Impairment (Low MMSE) among Community-Dwelling Elderly in Singapore.* Dement Geriatr Cogn Dis Extra, 2012. **2**(1): p. 529-42.

112. Wee, L.E., et al., *Individual and area-level socioeconomic status and their association with depression amongst community-dwelling elderly in Singapore.* Aging Ment Health, 2014. **18**(5): p. 628-41.
113. Weil, J., *Relationship to Place for Older Adults in a New York City Neighborhood Undergoing Gentrification: A Discourse Analysis.* City & Community, 2019. **18**(4): p. 1267-1286.
114. Wight, R.G., et al., *Urban neighborhood context, educational attainment, and cognitive function among older adults.* Am J Epidemiol, 2006. **163**(12): p. 1071-8.
115. Wight, R.G., et al., *A multilevel analysis of urban neighborhood socioeconomic disadvantage and health in late life.* Soc Sci Med, 2008. **66**(4): p. 862-72.
116. Wight, R.G., et al., *Urban neighborhood context and change in depressive symptoms in late life.* J Gerontol B Psychol Sci Soc Sci, 2009. **64**(2): p. 247-51.
117. Wight, R.G., et al., *Urban neighborhood context and mortality in late life.* Journal of Aging and Health, 2010. **22**(2): p. 197-218.
118. Wörn, J., et al., *Cognitive functioning among Dutch older adults: Do neighborhood socioeconomic status and urbanity matter?* Soc Sci Med, 2017. **187**: p. 29-38.
119. Wu, Y.T., et al., *Community environment, cognitive impairment and dementia in later life: results from the Cognitive Function and Ageing Study.* Age Ageing, 2015. **44**(6): p. 1005-11.
120. Yan, T., et al., *Exploring psychosocial pathways between neighbourhood characteristics and stroke in older adults: the cardiovascular health study.* Age Ageing, 2013. **42**(3): p. 391-7.
121. Yao, L. and S.A. Robert, *The contributions of race, individual socioeconomic status, and Neighborhood socioeconomic context on the self-rated health trajectories and mortality of older adults.* Research on Aging, 2008. **30**(2): p. 251-273.
122. Yao, L. and S.A. Robert, *Examining the Racial Crossover in Mortality between African American and White Older Adults: A Multilevel Survival Analysis of Race, Individual Socioeconomic Status, and Neighborhood Socioeconomic Context.* J Aging Res, 2011. **2011**: p. 132073.
