# Supplementary material for: Neighbourhood Socioeconomic Processes and Dynamics and Healthy Ageing: A Scoping Review
Source: Int J Environ Res Public Health. 2022 May 31;19(11):6745. doi: 10.3390/ijerph19116745 (PMC9180257; doi:10.3390/ijerph19116745)
Supplement: Supplementary file 1 [file ijerph-19-06745-s001.zip › Supplementary material - IV - Outcomes.pdf]

Table S4: Categorisation of the outcomes studied into the domain(s) of healthy ageing

<table>
<tr><th>Domain(s) of healthy ageing</th><th>Outcomes included</th><th>Definition(s) used</th></tr>
<tr><td rowspan="12">Neurological health</td><td>Alzheimer's disease</td><td>Defined as the presence of either diffuse plaques or neuritic plaques, following the National Institute of Aging and the Alzheimer Association criteria for neuropathology plaque presence (Powell et al., 2020)</td></tr>
<tr><td>Anxiety and Depression</td><td>Patient Health Questionnaire-4 that sees if respondents had little interest, felt down, felt nervous, or was unable to stop worrying over the past month (Smith, Lehning, & Kim, 2018)</td></tr>
<tr><td>Anxiety</td><td>Anxiety was assessed with anxiety subscale of the General Health Questionnaire (Walters et al., 2004)</td></tr>
<tr><td rowspan="4">Depression</td><td>Assessed by the Primary Health Questionnaire (PHQ-9) (Almeida et al., 2012)</td></tr>
<tr><td>Depression was assessed with the Geriatric Depression Scale (Bolstad, Moak, Brown, Kennedy, & Buys, 2020; Guo, Chang, et al., 2019; Kwag, Jang, Rhew, & Chiriboga, 2011; Walters et al., 2004; Wee et al., 2014)</td></tr>
<tr><td>A dichotomous measure derived from the Centers for Epidemiologic Studies Depression Scale (Cagney, Browning, Iveniuk, & English, 2014)</td></tr>
<tr><td>Measured using the 10-item Hopkins Symptom Checklist considering two dimensions of mental disorder: anxiety (4 questions) and depression (6 questions) (Miao, Wu, & Sun, 2019)</td></tr>
<tr><td rowspan="3">Depression symptoms</td><td>Measured using a version of the 20-item Center for Epidemiologic Studies–Depression scale (Hybels et al., 2006; Kubzansky et al., 2005; Ostir, Eschbach, Markides, & Goodwin, 2003)</td></tr>
<tr><td>Measured with the 9-item Patient Health Questionnaire that assesses depressive symptoms in the past month (Joshi et al., 2017)</td></tr>
<tr><td>Measured with eight items from the longer Center for Epidemiologic Studies–Depression scale (Aneshensel et al., 2007; Wight, Cummings, Karlamangla, & Aneshensel, 2009)</td></tr>
<tr><td>Psychosocial functioning</td><td>Assessed with three measures: depression symptoms (10-item version of the Center for Epidemiologic Studies Depression Scale); hostility (8 items from the Cook-Medley Hostility Scale); and perceived stress (6-item version of the Perceived Stress Scale) (Everson-Rose et al., 2011)</td></tr>
</table>

	Cognitive decline	Measured with the abbreviated version of the modified Telephone Interview for Cognitive Status (Casanova et al., 2020)
		Measured with the Modified Mini Mental State Examination (Hazzouri et al., 2011)
		Assessed using the Mini-Mental State Examination. Probable cognitive decline is indicated by a score of below 19 points for those who are illiterate and below 24 points for those with some education (Danielewicz, Wagner, d'Orsi, & Boing, 2016)
		Diagnosed if participants scored below a 1.5 standard deviation on the mini-mental status examination (Kim et al., 2017)
	Cognitive impairment	Cognitive impairment was defined when the Mini-Mental State Examination score was < 24 (Basta, Matthews, Chatfield, Brayne, & MRC-CFAS, 2007; Espino, Lichtenstein, Palmer, & Hazuda, 2001; Wee et al., 2012)
		Cognitive diagnoses were agreed between neurologists and neuropsychologists at each visit, taking into account his/her age, gender, cognitive reserve, functional information, and cognitive scores (Fernández-Blázquez et al., 2020)
	Cognitive function	Measured using the Mini-Mental State Examination (Guo, Chan, Chang, Liu, & Yip, 2019; Sheffield & Peek, 2009; Wörn, Ellwardt, Aartsen, & Huisman, 2017; Wu et al., 2015) or with the Modified Mini-Mental State Examination (Shih et al., 2011)
		Measured by using day and date questions from the Mini-Mental State Examination to check time orientation; 10-word learning task from the Health and Retirement Study to see verbal memory; remembering a task to perform later to assess prospective memory; thinking of words of a category to check for verbal fluency; and letter cancellation task from the National Study of Health and Development to measure attention and mental speed (Lang, Llewellyn, Langa, Wallace, Huppert, et al., 2008)
		Assessed with different tests for: memory, reasoning, processing speed, and everyday cognition (Meyer et al., 2017)

		Assessed annually using the Digit Symbol Substitution Test and the Modified Mini-Mental State Examination (Rosso et al., 2016)
		Measured based on: memory, inductive reasoning, processing speed, everyday cognition, and vocabulary (Sisco & Marsiske, 2012)
		Assessed using the Telephone Interview for Cognitive Status (Wight et al., 2006)
	Dementia	Assessed using a combination of self-reported or informant-reported physician diagnosis of dementia or Alzheimer disease or a score above the threshold of 3.38 on the 16-question Informant Questionnaire on Cognitive Decline in the Elderly (Cadar et al., 2018)
		Participants were examined by a neurologist and an independent committee of neurologists according to the Diagnostic and Statistical Manual of Mental Disorders IV criteria (Letellier et al., 2017)
		Participants were evaluated by a trained psychologist with neuropsychological tests (Mini-Mental State 130 Examination) and reviewed by an independent committee of neurologists to obtain a consensus on the diagnosis according to the DSM-IV criteria (Letellier et al., 2019)
	Mental health	Measured by the General Health Questionnaire (GHQ12) tool to detect mental disorders in the general population (Behanova et al., 2017)
		Assessed using the U.S. Centers for Medicare and Medicaid Services' (CMS') Master Beneficiary Summary File using algorithms for diagnoses of each of Alzheimer's disease and depression (S. C. Brown et al., 2018)
		Assessed with the Warwick-Edinburgh Mental Mental wellbeing Scale, which includes: positive affect, psychological functioning, and interpersonal relationships (Gale, Dennison, Cooper, & Sayer, 2011)
		Assessed with the 10-item Center for Epidemiologic Studies Depression scale (Kelley-Moore, Cagney, Skarupski, Everson-Rose, & Mendes de Leon, 2016)
		Measured by the Hospital Anxiety and Depression Scale (Pearce, Cherrie, Shortt, Deary, & Thompson, 2018)
		Assessed using SF-12 Mental Component Summary score, which includes: physical functioning; role

		limitations due to physical health problems; bodily pain; general health; vitality (energy/fatigue); social functioning; role limitations due to emotional problems; and mental health (Salvatore & Grundy, 2021)
Social/physical functioning	Functional limitation	Measured with difficulty in 1 or more of 6 self-reported activities of daily living (ADL) OR 4 instrumental ADL (Jung, Kind, Robert, Buckingham, & DuGoff, 2018)
	Functional impairment	Determined by the activities of daily living self-reported scale (Basta et al., 2007)
	Functional status	Assessed with reported difficulties with both basic activities of daily living and instrumental activities of daily living (Wight et al., 2008)
	Physical health	It was measured participants' subjective health perceptions based on the Older American Resources and Services Questionnaire (Kwag et al., 2011)
	Social and physical functioning	Social functioning: social activity and social contacts in the past month and physical functioning was measured by a scale of level of physical difficulty with everyday activities and instrumental Activities of Daily Living (Bowling & Stafford, 2007)
	Falls	Defined as being an event that resulted in unintentionally coming to rest on the ground or a lower surface (Li et al., 2014)
		Based on: muscle weakness according to self-report, history of falls in the past year, gait deficit (gait speed <0.8 m/s), balance deficit according to in-person examination, use of an assistive device according to in-person assessment, visual deficit as determined using a standardized visual acuity examination, history of arthritis according to self-report, self-reported impairment in one or more activity of daily living, depression based on the 15-item Geriatric Depression Scale, and cognitive impairment based on the Mini-Mental State Examination (Lo et al., 2016)
	Mobility impairment	Mobility impairment was self-reported and considered if the participant reported difficulty either: walking a half-mile or 2) walking up ten steps (Nordstrom et al., 2007)

	Mobility disability	Self-reported by questioning if participants experienced difficulties walking 100 yards or climbing one flight of stairs without resting due to health problems for at least more than 3 months. Gait speed was measured with a test that assessed how long it took to walk a distance of eight feet (Lang, Llewellyn, Langa, Wallace, & Melzer, 2008)
	Disability	Disability data from the long-form (SF3) (Beard et al., 2009)
	Frailty	Defined as the presence of five frailty characteristics: walking speed standardized based on median height and sex, grip strength standardized based on body mass index and sex, energy expenditure standardized based on sex, exhaustion based on self-report, and weight loss (Espinoza & Hazuda, 2015)
		Assessed with the 45 items TOPICS-Frailty Index (Franse et al., 2017)
		The frailty index was calculated based on sensory and functional impairments, self-reported comorbidities, poor or fair self-rated health, low mood or depression measured according to the eight-item Center for Epidemiological Studies Depression Scale and a score in the lowest 10% of composite cognitive function testing (Lang et al., 2009)
Health behaviours	Diet	Serum carotene, b-carotene, b-cryptoxanthin, lutein+zeaxanthin, and lycopene were analysed in nonfasting participants from blood samples (Nicklett et al., 2011)
	Physical exercise	Measured by the Neighbourhood Walking Questionnaire – Chinese version for Seniors (Cerin et al., 2013)
		Measured with the question: "During the last week, about how many blocks did you walk?" (Hannon, Sawyer, & Allman, 2012)
	Physical activity	Leisure-time physical activity (LTPA) was assessed by a recall survey that indicated the number of times during the previous two weeks that the participant engaged in LTPAs (Annear, Gidlow, & Cushman, 2009)
		Semi-structured interview identified how older adults perceived their local environment and identified factors that influence their active leisure participation (Annear, Cushman, & Gidlow, 2009)

		Measured by an accelerometer for seven days (Fox et al., 2011)
		Assessed through two measures: walking for transportation and for leisure based on the long version of the International Physical Activity Questionnaire (Giehl, Hallal, Weber Corseuil, Schneider, & d'Orsi, 2016)
		Measured with a GT3x accelerometer over the right hip for seven days during waking hours (100–1040 minutes for light-intensity physical activity and > 1040 for moderate to vigorous physical activity) (Hawkesworth et al., 2018)
		Measured by using 16 items derived from the Physical Activity Scale for the Elderly to assess past-week physical activity (Mooney et al., 2017)
		Measured with the EPIPorto Physical Activity Questionnaire to measure time and intensity of leisure-time physical activity either light (e.g. brisk walking, golfing, snooker), moderate (e.g. walk at moderate pace, dancing, stretching) or vigorous (e.g. running, soccer, basketball) (Ribeiro, Pires, Carvalho, & Pina, 2015)
	Drinking problems	Measured by using the 4-item Cut, Annoyed, Guilty, Eye Opener instrument for detecting alcohol abuse amongst Latinos (Stroope, Martinez, Eschbach, Peek, & Markides, 2015)
	Health-risk behaviours	Five indicators: daily smoking ("Do you currently smoke?"), binge drinking (How often do you drink six portions or more of alcohol at once"?), physical activity (In your leisure time, how often do you do physical exercise for at least 30 min which makes you at least mildly short of breath or perspire?"), consumption of fruits and vegetables ("On average, how many portions of fruit/vegetables do you eat a day?" and BMI (calculated from the self-reported height and weight) (Behanova et al., 2015)
	Health behaviours	Diet was assessed using a Food Frequency Questionnaire summarized by frequency of consumption. Smoking habits (current and past) were asked. Physical activity was assessed by asking participants their habitual physical activity in hours per week and to rate their own walking speeds (Amuzu, Carson, Watt, Lawlor, & Ebrahim, 2009)

Well-being/quality of life	Anger	It was measured based on an index created with the question: “In the past week, on how many days did you”: “feel very critical of others,” “become easily annoyed or irritated,” “argue with someone,” “feel angry,” and “yell at someone.” The response categories were “no days”, “1 or 2 days”, “3 or 4 days”, and “5 or more days” (Schieman, Pearlin, & Meersman, 2006)
	Health conditions	It was chosen 12 health measures including: arthritis, diabetes, hypertension, congestive heart failure, ischemic heart disease, and chronic obstructive pulmonary disease (chronic physical health conditions), dementia and depression (mental health conditions), acute myocardial infarction, stroke, falls, and hip fractures (acute conditions) (Menec, Shooshtari, Nowicki, & Fournier, 2010)
	Loneliness	Self-reported loneliness was assessed through the question: “Says or indicates that he/she feels lonely.” (Beere, Keeling, & Jamieson, 2019)
		Assessed using the De Jong Gierveld Loneliness Scale (Timmermans et al., 2020)
	Overall well-being	Assessed the intention of elderly people to remain active taking into account social, symbolic, and spatial variables (Domínguez-Parraga, 2020)
	Psychological distress	Assessed using the self-completion 30-item General Health Questionnaire (Sarkar, Gallacher, & Webster, 2013)
	Quality of life	WHO quality of life assessment tool (WHOQoL-BREF) across four domains: physical, psychological, social, and environment (Alaazi, Menon, Stafinski, Hodgins, & Jhangri, 2021)
		Measured in interviews based on a 17-item measure of the UK version of the Sickness Impact Profile and the Philadelphia Geriatric Morale Scale (Breeze et al., 2005)
		Measured by the 26-item WHOQOL-BREF that groups into 4 major domains of life quality: physical (7 items), psychological (6 items), social (3 items), and environmental (8 items) (Mõttus, Gale, Starr, & Deary, 2012)
	Shopping difficulty	Assessed as a 5-level measure of shopping difficulty based on participants' past month reported shopping (Brenner & Clarke, 2019)

	Social exclusion	Measured by the experiences of living in the area and whether these had changed over time; views about the physical environment, including transportation, public space and housing; and perceptions of community life and neighbourhood relationships (Buffel & Phillipson, 2019)
		Measured by participants' experiences of neighbourhood exclusion and inclusion (Buffel, Phillipson, & Scharf, 2013)
		Explore to what extent older people's social networks and activities were located inside and outside of their neighbourhood (Burns, Lavoie, & Rose, 2012)
Endocrine, nutritional, and metabolic health group	Body mass index	Measured based on a calculation using objectively-assessed weight and height where body mass index was categorised as: ≤ 24.9 kg/m2 for normal/underweight and ≥ 25 kg/m2 for overweight/obese (Van Dyck et al., 2020) (Burns et al., 2012)
	Diabetes	Type-2 diabetes was assessed as fasting glucose level 126 mg/dl, self-report of a physician diagnosis of diabetes, and/or documented use of prescription diabetes medication. Pre-diabetes as fasting blood glucose level between 100 mg/dL and 125 mg/dL (Garcia et al., 2015; Garcia et al., 2016)
	Metabolic conditions	Metabolic conditions included: obesity (body-mass index 30 kg/m2), diabetes (self-reported history and/or use of glucose-lowering medication), hypertension (self-reported history, antihypertensive medicine use, systolic blood pressure 140 mmHg, and/or diastolic blood pressure 90 mm Hg), and hyperlipidemia (total cholesterol 200 mg/dl and/or lipid-lowering medication use) (Corriere et al., 2014)
	Obesity	Abdominal obesity was defined as waist circumference (WC) according to World Health Organization (WHO) guidelines (obesity: men=102cm and women=88cm) (Araújo et al., 2018), and overall obesity was defined according to the WHO cutoff points (obesity: BMI > 30kg/m^2) (Araújo et al., 2018; Michael, Nagel, Gold, & Hillier, 2014)
	Chronic inflammation	Assessed with blood samples to ascertain levels of coagulation and inflammatory factors, including IL-6 (Purser et al., 2008)

	Progressive chronic kidney disease	Assessed with serum creatinine increase ≥0.4 mg/dL (Merkin et al., 2007)
Mortality	All-cause mortality	Extracted from Statistics Denmark (Meijer et al., 2012)
	Cardiovascular disease mortality	Classified in the International Classification of Diseases, ninth revision codes of 401–459 through the National Health Service Central Register (Ramsay et al., 2015)
	Ischaemic heart disease mortality	Assessed with the International Classification of Diseases (ICD) as ICD-9 codes 410–414 or ICD-10 codes I20–I25 (Chaix, Rosvall, & Merlo, 2007)
	Mortality	Reviews of obituaries, medical records, death certificates, and the US Health Care Financing Administration health care utilisation database for stays in the hospital was used to confirm deaths of participants (Diez Roux, Borrell, Haan, Jackson, & Schultz, 2004)
		Assessed with the National Death Index (L. Yao & Robert, 2011)
		Assessed with Assets and Health Dynamics Among the Oldest Old/Health and Retirement Study Tracker File where the deaths are verified through the National Death Index (Wight, Cummings, Karlamangla, & Aneshensel, 2010)
	Stroke mortality	Death certificates from 2005 to 2009 for Arkansas that coded stroke as an underlying cause of death with International Classification of Diseases, Tenth Revision codes I60 to I69 (Balamurugan, Delongchamp, Bates, & Mehta, 2013)
Cardiovascular health	Cardiovascular disease	Composite index of subclinical atherosclerosis and cardiovascular disease developed in the Cardiovascular Health Study (Nordstrom, Diez Roux, Jackson, & Gardin, 2004)
		Self-reported physician-diagnosed cardiovascular disease was assessed through three questions: if a doctor ever told them, they had a heart problem, a stroke or diabetes (Wight et al., 2008)
	Coronary heart disease	Assessed with participants' medical records or by self-report of a physician diagnosis (Lawlor, Davey Smith, Patel, & Ebrahim, 2005)
		Based on WHO's International Classification of Diseases (ICD), that is, ICD-7 code 420, ICD-8 and ICD-9 codes 410, 411, 412, 413 and 414, and ICD-10 codes I20, I21, I22, I23, I24 and I25 (Lönn, Melander, Crump, & Sundquist, 2019)

	Hypertension	Prevalent hypertension was classified when the systolic blood pressure was 140 millimetres of mercury or higher, the diastolic blood pressure was 90 millimetres of mercury or higher, or if the participant was taking antihypertensive medication (Buys et al., 2015; Wagner, Boing, Subramanian, Höfelmann, & D'Orsi, 2016)
		Hypertension was categorised into 4 categories: no hypertension, prehypertension (at least 2 readings of ≥120), hypertension (3 readings of ≥140, and very high hypertension (at least 2 readings of ≥160) (Nguyen, 2016)
	Ischemic stroke	It was classified as a first ischemic stroke adjudicated by a cerebrovascular disease endpoint committee that also classified stroke subtype and determined whether death was caused by stroke (A. F. Brown et al., 2011)
	Stroke	Assessed with reviews at annual visits, interim telephone contacts, notification of events by participants and review of Medicare hospitalisation data (Yan et al., 2013)
Access to healthcare	Access to healthcare	Having problems accessing health care was assessed based on the following parameters: the respondent had no particular person or place that they usually go when they are sick or needs health advice; during the past 12 months, the respondent delayed seeking medical care because of worry about the cost; and during the past 12 months, the respondent needed care but did not get at least one of the following: (a) medical or surgical care, (b) dental care, (c) mental health care, (d) prescription medication, or (e) eyeglasses (Auchincloss, Van Nostrand, & Ronsaville, 2001)
		Assessed with emergency department visits from all local hospital systems (Huang, Meyer, & Jin, 2018)
Ageing in place	Ageing in place	Defined there as "growing old in one's own home and neighbourhood" (Lager, Van Hoven, & Huigen, 2013)
		Narratives about the meaning of place and gentrification (Weil, 2019)
Life expectancy/Years of potential life lost	Life expectancy	Life expectancy at age 30 was estimated using a skew-normal regression approach accounting for left-truncated and right-censored observations (Moser et al., 2014)
	Years of potential life lost	Calculated by subtracting the age at the time of death from a predetermined endpoint age (100 years old) (Bhardwaj, Amiri, Buchwald, & Amram, 2020)

Morbidity	Multimorbidity	Assessed with the International Classification of Diseases, Ninth & Tenth Revision codes to define the 20 chronic conditions identified by the United States Department of Health and Human Services for studying multimorbidity (hypertension, hyperlipidemia, diabetes, coronary artery disease, congestive heart failure, cardiac arrhythmias, stroke, asthma, chronic obstructive pulmonary disease, arthritis, osteoporosis, chronic kidney disease, cancer, autism spectrum disorder, hepatitis, human immunodeficiency virus, depression, dementia, schizophrenia, and substance abuse disorders) (Chamberlain et al., 2020)
Musculoskeletal system	Hip fracture	Measured based on the ninth version of the International Classification of Diseases from the Hospital Discharge Register of the Health Care Board of Stockholm County Council (Reimers & Laflamme, 2007)
Oral health	Dental service use	Measured with questions related to the presence of participants own teeth or if they have already lost all of them. In case participants had their own teeth it was asked: 'in general, do you go to the dentist for a regular check-up or only when you are having trouble with your teeth?' where possible answers were: 'regular check-up', 'occasional check-up', 'only when having trouble' and 'never go to the dentist'. (Lang, Gibbs, Steel, & Melzer, 2008)
Self-rated health	Self-rated health	Self-rated health was assessed with three individual-level measures regarding the general health status and the number of healthy days that related to the quality of life (Deng & Mao, 2018)
		Participants were asked to rate their health, using a four-point rating system: poor, fair, good, or excellent (Ko, Jang, Park, Rhew, & Chiriboga, 2014; Patel, Eschbach, Rudkin, Peek, & Markides, 2003; Smith et al., 2018; Stroope et al., 2017; Subramanian, Kubzansky, Berkman, Fay, & Kawachi, 2006)
		Participants were asked to assess their health in excellent, very good, good, fair, and poor (Omariba, 2010; Robert & Ruel, 2006; Smith et al., 2018; Wight et al., 2008; Li Yao & Robert, 2008)

Alaazi, D. A., Menon, D., Stafinski, T., Hodgins, S., & Jhangri, G. (2021). Quality of life of older adults in two contrasting neighbourhoods in Accra, Ghana. *Social Science and Medicine, 270*. doi:10.1016/j.socscimed.2020.113659

Almeida, O. P., Pirkis, J., Kerse, N., Sim, M., Flicker, L., Snowdon, J., . . . Pfaff, J. J. (2012). Socioeconomic disadvantage increases risk of prevalent and persistent depression in later life. *J Affect Disord, 138*(3), 322-331. doi:10.1016/j.jad.2012.01.021

Amuzu, A., Carson, C., Watt, H. C., Lawlor, D. A., & Ebrahim, S. (2009). Influence of area and individual lifecourse deprivation on health behaviours: findings from the British Women's Heart and Health Study. *European journal of cardiovascular prevention and rehabilitation, 16*(2), 169-173. doi:10.1097/HJR.0b013e328325d64d

Aneshensel, C. S., Wight, R. G., Miller-Martinez, D., Botticello, A. L., Karlamangla, A. S., & Seeman, T. E. (2007). Urban neighborhoods and depressive symptoms among older adults. *J Gerontol B Psychol Sci Soc Sci, 62*(1), S52-59. doi:10.1093/geronb/62.1.s52

Annear, M. J., Cushman, G., & Gidlow, B. (2009). Leisure time physical activity differences among older adults from diverse socioeconomic neighborhoods. *Health Place, 15*(2), 482-490. doi:10.1016/j.healthplace.2008.09.005

Annear, M. J., Gidlow, B., & Cushman, G. (2009). Neighbourhood deprivation and older adults' preferences for and perceptions of active leisure participation. *Annals of Leisure Research, 12*(2), 96-128. doi:10.1080/11745398.2009.9686814

Araújo, C. A. H., Giehl, M. W. C., Danielewicz, A. L., Araujo, P. G., d'Orsi, E., & Boing, A. F. (2018). Built environment, contextual income, and obesity in older adults: evidence from a population-based study. *Cad Saude Publica, 34*(5), e00060217. doi:10.1590/0102-311x00060217

Auchincloss, A. H., Van Nostrand, J. F., & Ronsaville, D. (2001). Access to health care for older persons in the United States: personal, structural, and neighborhood characteristics. *J Aging Health, 13*(3), 329-354. doi:10.1177/089826430101300302

Balamurugan, A., Delongchamp, R., Bates, J. H., & Mehta, J. L. (2013). The neighborhood where you live is a risk factor for stroke. *Circulation: Cardiovascular Quality and Outcomes, 6*(6), 668-673. doi:10.1161/CIRCOUTCOMES.113.000265

Basta, N. E., Matthews, F. E., Chatfield, M. D., Brayne, C., & MRC-CFAS. (2007). Community-level socio-economic status and cognitive and functional impairment in the older population. *Eur J Public Health, 18*(1), 48-54. doi:10.1093/eurpub/ckm076

Beard, J. R., Blaney, S., Cerda, M., Frye, V., Lovasi, G. S., Ompad, D., . . . Vlahov, D. (2009). Neighborhood characteristics and disability in older adults. *J Gerontol B Psychol Sci Soc Sci, 64*(2), 252-257. doi:10.1093/geronb/gbn018

Beere, P., Keeling, S., & Jamieson, H. (2019). Ageing, loneliness, and the geographic distribution of New Zealand's interRAI-HC cohort. *Soc Sci Med, 227*, 84-92. doi:10.1016/j.socscimed.2018.08.002

Behanova, M., Katreniakova, Z., Nagyova, I., van Ameijden, E. J., Dijkshoorn, H., van Dijk, J. P., & Reijneveld, S. A. (2015). The effect of neighbourhood unemployment on health-risk behaviours in elderly differs between Slovak and Dutch cities. *Eur J Public Health, 25*(1), 108-114. doi:10.1093/eurpub/cku116

Behanova, M., Katreniakova, Z., Nagyova, I., van Ameijden, E. J. C., van Dijk, J. P., & Reijneveld, S. A. (2017). Elderly from lower socioeconomic groups are more vulnerable to mental health problems, but area deprivation does not contribute: a comparison between Slovak and Dutch cities. *Eur J Public Health, 27*(suppl_2), 80-85. doi:10.1093/eurpub/ckv096

Bhardwaj, R., Amiri, S., Buchwald, D., & Amram, O. (2020). Environmental Correlates of Reaching a Centenarian Age: Analysis of 144,665 Deaths in Washington State for 2011-2015. *Int J Environ Res Public Health, 17*(8). doi:10.3390/ijerph17082828

Bolstad, C. J., Moak, R., Brown, C. J., Kennedy, R. E., & Buys, D. R. (2020). Neighborhood Disadvantage Is Associated with Depressive Symptoms but Not Depression Diagnosis in Older Adults. *Int J Environ Res Public Health, 17*(16). doi:10.3390/ijerph17165745

Bowling, A., & Stafford, M. (2007). How do objective and subjective assessments of neighbourhood influence social and physical functioning in older age? Findings from a British survey of ageing. *Soc Sci Med, 64*(12), 2533-2549. doi:10.1016/j.socscimed.2007.03.009

Breeze, E., Jones, D. A., Wilkinson, P., Bulpitt, C. J., Grundy, C., Latif, A. M., & Fletcher, A. E. (2005). Area deprivation, social class, and quality of life among people aged 75 years and over in Britain. *Int J Epidemiol, 34*(2), 276-283. doi:10.1093/ije/dyh328

Brenner, A. B., & Clarke, P. J. (2019). Difficulty and independence in shopping among older Americans: more than just leaving the house. *Disabil Rehabil, 41*(2), 191-200. doi:10.1080/09638288.2017.1398785

Brown, A. F., Liang, L. J., Vassar, S. D., Stein-Merkin, S., Longstreth, W. T., Jr., Ovbiagele, B., . . . Escarce, J. J. (2011). Neighborhood disadvantage and ischemic stroke: the Cardiovascular Health Study (CHS). *Stroke, 42*(12), 3363-3368. doi:10.1161/strokeaha.111.622134

Brown, S. C., Perrino, T., Lombard, J., Wang, K., Toro, M., Rundek, T., . . . Szapocznik, J. (2018). Health Disparities in the Relationship of Neighborhood Greenness to Mental Health Outcomes in 249,405 US Medicare Beneficiaries. *Int J Environ Res Public Health, 15*(3). doi:10.3390/ijerph15030430

Buffel, T., & Phillipson, C. (2019). Ageing in a Gentrifying Neighbourhood: Experiences of Community Change in Later Life. *Sociology-the Journal of the British Sociological Association, 53*(6), 987-1004. doi:10.1177/0038038519836848

Buffel, T., Phillipson, C., & Scharf, T. (2013). Experiences of neighbourhood exclusion and inclusion among older people living in deprived inner-city areas in Belgium and England. *Ageing & Society, 33*, 89-109. doi:10.1017/s0144686x12000542

Burns, V. F., Lavoie, J. P., & Rose, D. (2012). Revisiting the role of neighbourhood change in social exclusion and inclusion of older people. *J Aging Res, 2012*, 148287. doi:10.1155/2012/148287

Buys, D. R., Howard, V. J., McClure, L. A., Buys, K. C., Sawyer, P., Allman, R. M., & Levitan, E. B. (2015). Association between neighborhood disadvantage and hypertension prevalence, awareness, treatment, and control in older adults: results from the University of Alabama at Birmingham Study of Aging. *Am J Public Health, 105*(6), 1181-1188. doi:10.2105/ajph.2014.302048

Cadar, D., Lassale, C., Davies, H., Llewellyn, D. J., Batty, G. D., & Steptoe, A. (2018). Individual and Area-Based Socioeconomic Factors Associated With Dementia Incidence in England: Evidence From a 12-Year Follow-up in the English Longitudinal Study of Ageing. *JAMA Psychiatry, 75*(7), 723-732. doi:10.1001/jamapsychiatry.2018.1012

Cagney, K. A., Browning, C. R., Iveniuk, J., & English, N. (2014). The onset of depression during the great recession: foreclosure and older adult mental health. *Am J Public Health, 104*(3), 498-505. doi:10.2105/ajph.2013.301566

Casanova, R., Saldana, S., Lutz, M. W., Plassman, B. L., Kuchibhatla, M., & Hayden, K. M. (2020). Investigating predictors of cognitive decline using machine learning. *Journals of Gerontology - Series B Psychological Sciences and Social Sciences, 75*(4), 733-742. doi:10.1093/geronb/gby054

Cerin, E., Mellecker, R., Macfarlane, D. J., Barnett, A., Cheung, M. C., Sit, C. H., & Chan, W. M. (2013). Socioeconomic status, neighborhood characteristics, and walking within the neighborhood among older Hong Kong Chinese. *J Aging Health, 25*(8), 1425-1444. doi:10.1177/0898264313510034

Chaix, B., Rosvall, M., & Merlo, J. (2007). Assessment of the magnitude of geographical variations and socioeconomic contextual effects on ischaemic heart disease mortality: a multilevel survival analysis of a large Swedish cohort. *J Epidemiol Community Health, 61*(4), 349-355. doi:10.1136/jech.2006.047597

Chamberlain, A. M., Finney Rutten, L. J., Wilson, P. M., Fan, C., Boyd, C. M., Jacobson, D. J., . . . St Sauver, J. L. (2020). Neighborhood socioeconomic disadvantage is associated with multimorbidity in a geographically-defined community. *BMC Public Health, 20*(1). doi:10.1186/s12889-019-8123-0

Corriere, M. D., Yao, W., Xue, Q. L., Cappola, A. R., Fried, L. P., Thorpe, R. J., Jr., . . . Kalyani, R. R. (2014). The association of neighborhood characteristics with obesity and metabolic conditions in older women. *J Nutr Health Aging, 18*(9), 792-798. doi:10.1007/s12603-014-0477-5

Danielewicz, A. L., Wagner, K. J., d'Orsi, E., & Boing, A. F. (2016). Is cognitive decline in the elderly associated with contextual income? Results of a population-based study in southern Brazil. *Cad Saude Publica, 32*(5), e00112715. doi:10.1590/0102-311x00112715

Deng, G., & Mao, L. (2018). Spatially Explicit Age Segregation Index and Self-Rated Health of Older Adults in US Cities. *Isprs International Journal of Geo-Information, 7*(9). doi:10.3390/ijgi7090351

Diez Roux, A. V., Borrell, L. N., Haan, M., Jackson, S. A., & Schultz, R. (2004). Neighbourhood environments and mortality in an elderly cohort: results from the cardiovascular health study. *J Epidemiol Community Health, 58*(11), 917-923. doi:10.1136/jech.2003.019596

Domínguez-Parraga, L. (2020). The effects of gentrification on the elderly: A case study in the city of Cáceres. *Social Sciences, 9*(9). doi:10.3390/SOCSCI9090154

Espino, D. V., Lichtenstein, M. J., Palmer, R. F., & Hazuda, H. P. (2001). Ethnic differences in Mini-Mental State Examination (MMSE) scores: Where you live makes a difference. *J Am Geriatr Soc, 49*(5), 538-548. doi:10.1046/j.1532-5415.2001.49111.x

Espinoza, S. E., & Hazuda, H. P. (2015). Frailty prevalence and neighborhood residence in older Mexican Americans: the San Antonio longitudinal study of aging. *J Am Geriatr Soc, 63*(1), 106-111. doi:10.1111/jgs.13202

Everson-Rose, S. A., Skarupski, K. A., Barnes, L. L., Beck, T., Evans, D. A., & Mendes de Leon, C. F. (2011). Neighborhood socioeconomic conditions are associated with psychosocial functioning in older black and white adults. *Health Place, 17*(3), 793-800. doi:10.1016/j.healthplace.2011.02.007

Fernández-Blázquez, M. A., Noriega-Ruiz, B., Ávila-Villanueva, M., Valentí-Soler, M., Frades-Payo, B., Del Ser, T., & Gómez-Ramírez, J. (2020). Impact of individual and neighborhood dimensions of socioeconomic status on the prevalence of mild cognitive impairment over seven-year follow-up. *Aging Ment Health*, 1-10. doi:10.1080/13607863.2020.1725803

Fox, K. R., Hillsdon, M., Sharp, D., Cooper, A. R., Coulson, J. C., Davis, M., . . . Thompson, J. L. (2011). Neighbourhood deprivation and physical activity in UK older adults. *Health Place, 17*(2), 633-640. doi:10.1016/j.healthplace.2011.01.002

Franse, C. B., van Grieken, A., Qin, L., Melis, R. J. F., Rietjens, J. A. C., & Raat, H. (2017). Socioeconomic inequalities in frailty and frailty components among community-dwelling older citizens. *PLoS One, 12*(11), e0187946. doi:10.1371/journal.pone.0187946

Gale, C. R., Dennison, E. M., Cooper, C., & Sayer, A. A. (2011). Neighbourhood environment and positive mental health in older people: the Hertfordshire Cohort Study. *Health Place, 17*(4), 867-874. doi:10.1016/j.healthplace.2011.05.003

Garcia, L., Lee, A., Zeki Al Hazzouri, A., Neuhaus, J., Epstein, M., & Haan, M. (2015). The Impact of Neighborhood Socioeconomic Position on Prevalence of Diabetes and Prediabetes in Older Latinos: The Sacramento Area Latino Study on Aging. *Hisp Health Care Int, 13*(2), 77-85. doi:10.1891/1540-4153.13.2.77

Garcia, L., Lee, A., Zeki Al Hazzouri, A., Neuhaus, J. M., Moyce, S., Aiello, A., . . . Haan, M. N. (2016). Influence of neighbourhood socioeconomic position on the transition to type II diabetes in older Mexican Americans: the Sacramento Area Longitudinal Study on Aging. *BMJ Open, 6*(8), e010905. doi:10.1136/bmjopen-2015-010905

Giehl, M. C. G., Hallal, P. C., Weber Corseuil, C., Schneider, I. J., & d'Orsi, E. (2016). Built Environment and Walking Behavior Among Brazilian Older Adults: A Population-Based Study. *J Phys Act Health, 13*(6), 617-624. doi:10.1123/jpah.2015-0355

Guo, Y., Chan, C. H., Chang, Q., Liu, T., & Yip, P. S. F. (2019). Neighborhood environment and cognitive function in older adults: A multilevel analysis in Hong Kong. *Health Place, 58*, 102146. doi:10.1016/j.healthplace.2019.102146

Guo, Y., Chang, S. S., Chan, C. H., Chang, Q., Hsu, C. Y., & Yip, P. S. F. (2019). Association of neighbourhood social and physical attributes with depression in older adults in Hong Kong: a multilevel analysis. *J Epidemiol Community Health, 74*(2), 120-129. doi:10.1136/jech-2019-212977

Hannon, L., 3rd, Sawyer, P., & Allman, R. M. (2012). Housing, the Neighborhood Environment, and Physical Activity among Older African Americans. *J Health Dispar Res Pract, 5*(3), 27-41.

Hawkesworth, S., Silverwood, R. J., Armstrong, B., Pliakas, T., Nanchalal, K., Jefferis, B. J., . . . Lock, K. (2018). Investigating associations between the built environment

and physical activity among older people in 20 UK towns. *J Epidemiol Community Health, 72*(2), 121-131. doi:10.1136/jech-2017-209440

Hazzouri, A. Z. A., Haan, M. N., Osypuk, T., Abdou, C., Hinton, L., & Aiello, A. E. (2011). Neighborhood socioeconomic context and cognitive decline among older Mexican Americans: results from the Sacramento Area Latino Study on Aging. *Am J Epidemiol, 174*(4), 423-431. doi:10.1093/aje/kwr095

Huang, Y., Meyer, P., & Jin, L. (2018). Neighborhood socioeconomic characteristics, healthcare spatial access, and emergency department visits for ambulatory care sensitive conditions for elderly. *Prev Med Rep, 12*, 101-105. doi:10.1016/j.pmedr.2018.08.015

Hybels, C. F., Blazer, D. G., Pieper, C. F., Burchett, B. M., Hays, J. C., Fillenbaum, G. G., . . . Berkman, L. F. (2006). Sociodemographic characteristics of the neighborhood and depressive symptoms in older adults: using multilevel modeling in geriatric psychiatry. *Am J Geriatr Psychiatry, 14*(6), 498-506. doi:10.1097/01.Jgp.0000194649.49784.29

Joshi, S., Mooney, S. J., Rundle, A. G., Quinn, J. W., Beard, J. R., & Cerdá, M. (2017). Pathways from neighborhood poverty to depression among older adults. *Health Place, 43*, 138-143. doi:10.1016/j.healthplace.2016.12.003

Jung, D., Kind, A., Robert, S., Buckingham, W., & DuGoff, E. (2018). Linking Neighborhood Context and Health in Community-Dwelling Older Adults in the Medicare Advantage Program. *J Am Geriatr Soc, 66*(6), 1158-1164. doi:10.1111/jgs.15366

Kelley-Moore, J. A., Cagney, K. A., Skarupski, K. A., Everson-Rose, S. A., & Mendes de Leon, C. F. (2016). Do Local Social Hierarchies Matter for Mental Health? A Study of Neighborhood Social Status and Depressive Symptoms in Older Adults. *J Gerontol B Psychol Sci Soc Sci, 71*(2), 369-377. doi:10.1093/geronb/gbv047

Kim, G. H., Lee, H. A., Park, H., Lee, D. Y., Jo, I., Choi, S. H., . . . Jeong, J. H. (2017). Effect of Individual and District-level Socioeconomic Disparities on Cognitive Decline in Community-dwelling Elderly in Seoul. *J Korean Med Sci, 32*(9), 1508-1515. doi:10.3346/jkms.2017.32.9.1508

Ko, J. E., Jang, Y., Park, N. S., Rhew, S. H., & Chiriboga, D. A. (2014). Neighborhood effects on the self-rated health of older adults from four racial/ethnic groups. *Soc Work Public Health, 29*(2), 89-99. doi:10.1080/19371918.2013.829760

Kubzansky, L. D., Subramanian, S. V., Kawachi, I., Fay, M. E., Soobader, M. J., & Berkman, L. F. (2005). Neighborhood contextual influences on depressive symptoms in the elderly. *Am J Epidemiol, 162*(3), 253-260. doi:10.1093/aje/kwi185

Kwag, K. H., Jang, Y., Rhew, S. H., & Chiriboga, D. A. (2011). Neighborhood Effects on Physical and Mental Health: A Study of Korean American Older Adults. *Asian Am J Psychol, 2*(2), 91-100. doi:10.1037/a0023656

Lager, D., Van Hoven, B., & Huigen, P. P. P. (2013). Dealing with change in old age: Negotiating working-class belonging in a neighbourhood in the process of urban renewal in the Netherlands. *Geoforum, 50*, 54-61. doi:10.1016/j.geoforum.2013.07.012

Lang, I. A., Gibbs, S. J., Steel, N., & Melzer, D. (2008). Neighbourhood deprivation and dental service use: a cross-sectional analysis of older people in England. *J Public Health (Oxf), 30*(4), 472-478. doi:10.1093/pubmed/fdn047

Lang, I. A., Hubbard, R. E., Andrew, M. K., Llewellyn, D. J., Melzer, D., & Rockwood, K. (2009). Neighborhood deprivation, individual socioeconomic status, and frailty in older adults. *J Am Geriatr Soc, 57*(10), 1776-1780. doi:10.1111/j.1532-5415.2009.02480.x

Lang, I. A., Llewellyn, D. J., Langa, K. M., Wallace, R. B., Huppert, F. A., & Melzer, D. (2008). Neighborhood deprivation, individual socioeconomic status, and cognitive function in older people: analyses from the English Longitudinal Study of Ageing. *J Am Geriatr Soc, 56*(2), 191-198. doi:10.1111/j.1532-5415.2007.01557.x

Lang, I. A., Llewellyn, D. J., Langa, K. M., Wallace, R. B., & Melzer, D. (2008). Neighbourhood deprivation and incident mobility disability in older adults. *Age Ageing, 37*(4), 403-410. doi:10.1093/ageing/afn092

Lawlor, D. A., Davey Smith, G., Patel, R., & Ebrahim, S. (2005). Life-course socioeconomic position, area deprivation, and coronary heart disease: findings from the British Women's Heart and Health Study. *Am J Public Health, 95*(1), 91-97. doi:10.2105/AJPH.2003.035592

Letellier, N., Carrière, I., Gutierrez, L. A., Gabelle, A., Dartigues, J. F., Dufouil, C., . . . Berr, C. (2019). Influence of activity space on the association between neighborhood characteristics and dementia risk: results from the 3-City study cohort. *BMC Geriatr, 19*(1), 4. doi:10.1186/s12877-018-1017-7

Letellier, N., Gutierrez, L. A., Carrière, I., Gabelle, A., Dartigues, J. F., Dufouil, C., . . . Berr, C. (2017). Sex-specific association between neighborhood characteristics and dementia: The Three-City cohort. *Alzheimers Dement, 14*(4), 473-482. doi:10.1016/j.jalz.2017.09.015

Li, W., Procter-Gray, E., Lipsitz, L. A., Leveille, S. G., Hackman, H., Biondolillo, M., & Hannan, M. T. (2014). Utilitarian walking, neighborhood environment, and risk of outdoor falls among older adults. *Am J Public Health, 104*(9), e30-37. doi:10.2105/ajph.2014.302104

Lo, A. X., Rundle, A. G., Buys, D., Kennedy, R. E., Sawyer, P., Allman, R. M., & Brown, C. J. (2016). Neighborhood Disadvantage and Life-Space Mobility Are Associated with Incident Falls in Community-Dwelling Older Adults. *J Am Geriatr Soc, 64*(11), 2218-2225. doi:10.1111/jgs.14353

Lönn, S. L., Melander, O., Crump, C., & Sundquist, K. (2019). Accumulated neighbourhood deprivation and coronary heart disease: a nationwide cohort study from Sweden. *BMJ Open, 9*(9), e029248. doi:10.1136/bmjopen-2019-029248

Meijer, M., Kejs, A. M., Stock, C., Bloomfield, K., Ejstrud, B., & Schlattmann, P. (2012). Population density, socioeconomic environment and all-cause mortality: a multilevel survival analysis of 2.7 million individuals in Denmark. *Health Place, 18*(2), 391-399. doi:10.1016/j.healthplace.2011.12.001

Menec, V. H., Shooshtari, S., Nowicki, S., & Fournier, S. (2010). Does the relationship between neighborhood socioeconomic status and health outcomes persist into very old age? A population-based study. *J Aging Health, 22*(1), 27-47. doi:10.1177/0898264309349029

Merkin, S. S., Diez Roux, A. V., Coresh, J., Fried, L. F., Jackson, S. A., & Powe, N. R. (2007). Individual and neighborhood socioeconomic status and progressive chronic kidney disease in an elderly population: The Cardiovascular Health Study. *Soc Sci Med, 65*(4), 809-821. doi:10.1016/j.socscimed.2007.04.011

Meyer, O. L., Sisco, S. M., Harvey, D., Zahodne, L. B., Glymour, M. M., Manly, J. J., & Marsiske, M. (2017). Neighborhood Predictors of Cognitive Training Outcomes and Trajectories in ACTIVE. *Res Aging, 39*(3), 443-467. doi:10.1177/0164027515618242

Miao, J., Wu, X., & Sun, X. (2019). Neighborhood, social cohesion, and the Elderly's depression in Shanghai. *Soc Sci Med, 229*, 134-143. doi:10.1016/j.socscimed.2018.08.022

Michael, Y. L., Nagel, C. L., Gold, R., & Hillier, T. A. (2014). Does change in the neighborhood environment prevent obesity in older women? *Soc Sci Med, 102*, 129-137. doi:10.1016/j.socscimed.2013.11.047

Mooney, S. J., Joshi, S., Cerdá, M., Kennedy, G. J., Beard, J. R., & Rundle, A. G. (2017). Contextual Correlates of Physical Activity among Older Adults: A Neighborhood Environment-Wide Association Study (NE-WAS). *Cancer Epidemiol Biomarkers Prev, 26*(4), 495-504. doi:10.1158/1055-9965.Epi-16-0827

Moser, A., Panczak, R., Zwahlen, M., Clough-Gorr, K. M., Spoerri, A., Stuck, A. E., & Egger, M. (2014). What does your neighbourhood say about you? A study of life expectancy in 1.3 million Swiss neighbourhoods. *J Epidemiol Community Health, 68*(12), 1125-1132. doi:10.1136/jech-2014-204352

Mõttus, R., Gale, C. R., Starr, J. M., & Deary, I. J. (2012). 'On the street where you live': Neighbourhood deprivation and quality of life among community-dwelling older people in Edinburgh, Scotland. *Soc Sci Med, 74*(9), 1368-1374. doi:10.1016/j.socscimed.2011.12.050

Nguyen, H. V. (2016). Keeping Up with the Joneses: Neighbourhood Wealth and Hypertension. *Journal of Happiness Studies, 17*(3), 1255-1271. doi:10.1007/s10902-015-9641-9

Nicklett, E. J., Szanton, S., Sun, K., Ferrucci, L., Fried, L. P., Guralnik, J. M., & Semba, R. D. (2011). Neighborhood socioeconomic status is associated with serum carotenoid concentrations in older, community-dwelling women. *J Nutr, 141*(2), 284-289. doi:10.3945/jn.110.129684

Nordstrom, C. K., Diez Roux, A. V., Jackson, S. A., & Gardin, J. M. (2004). The association of personal and neighborhood socioeconomic indicators with subclinical cardiovascular disease in an elderly cohort. The cardiovascular health study. *Soc Sci Med, 59*(10), 2139-2147. doi:10.1016/j.socscimed.2004.03.017

Nordstrom, C. K., Diez Roux, A. V., Schulz, R., Haan, M. N., Jackson, S. A., & Balfour, J. L. (2007). Socioeconomic position and incident mobility impairment in the Cardiovascular Health Study. *BMC Geriatr, 7*, 11. doi:10.1186/1471-2318-7-11

Omariba, W. R. (2010). Neighbourhood characteristics, individual attributes and self-rated health among older Canadians. *Health and Place, 16*(5), 986-995. doi:10.1016/j.healthplace.2010.06.003

Ostir, G. V., Eschbach, K., Markides, K. S., & Goodwin, J. S. (2003). Neighbourhood composition and depressive symptoms among older Mexican Americans. *J Epidemiol Community Health, 57*(12), 987-992. doi:10.1136/jech.57.12.987

Patel, K. V., Eschbach, K., Rudkin, L. L., Peek, M. K., & Markides, K. S. (2003). Neighborhood context and self-rated health in older Mexican Americans. *Ann Epidemiol, 13*(9), 620-628. doi:10.1016/S1047-2797(03)00060-7

Pearce, J., Cherrie, M., Shortt, N., Deary, I., & Thompson, C. W. (2018). Life course of place: A longitudinal study of mental health and place. *Transactions of the Institute of British Geographers, 43*(4), 555-572. doi:10.1111/tran.12246

Powell, W. R., Buckingham, W. R., Larson, J. L., Vilen, L., Yu, M., Salamat, M. S., . . . Kind, A. J. H. (2020). Association of Neighborhood-Level Disadvantage With Alzheimer Disease Neuropathology. *JAMA Netw Open, 3*(6), e207559. doi:10.1001/jamanetworkopen.2020.7559

Purser, J. L., Kuchibhatla, M. N., Miranda, M. L., Blazer, D. G., Cohen, H. J., & Fillenbaum, G. G. (2008). Geographical segregation and IL-6: a marker of chronic inflammation in older adults. *Biomark Med, 2*(4), 335-348. doi:10.2217/17520363.2.4.335

Ramsay, S. E., Morris, R. W., Whincup, P. H., Subramanian, S. V., Papacosta, A. O., Lennon, L. T., & Wannamethee, S. G. (2015). The influence of neighbourhood-level socioeconomic deprivation on cardiovascular disease mortality in older age: longitudinal multilevel analyses from a cohort of older British men. *J Epidemiol Community Health, 69*(12), 1224-1231. doi:10.1136/jech-2015-205542

Reimers, A., & Laflamme, L. (2007). Hip fractures among the elderly: personal and contextual social factors that matter. *J Trauma, 62*(2), 365-369. doi:10.1097/01.ta.0000221669.26191.59

Ribeiro, A. I., Pires, A., Carvalho, M. S., & Pina, M. F. (2015). Distance to parks and non-residential destinations influences physical activity of older people, but crime doesn't: a cross-sectional study in a southern European city. *BMC Public Health, 15*, 593. doi:10.1186/s12889-015-1879-y

Robert, S. A., & Ruel, E. (2006). Racial segregation and health disparities between Black and White older adults. *J Gerontol B Psychol Sci Soc Sci, 61*(4), S203-211. doi:10.1093/geronb/61.4.s203

Rosso, A. L., Flatt, J. D., Carlson, M. C., Lovasi, G. S., Rosano, C., Brown, A. F., . . . Gianaros, P. J. (2016). Neighborhood Socioeconomic Status and Cognitive Function in Late Life. *Am J Epidemiol, 183*(12), 1088-1097. doi:10.1093/aje/kwv337

Salvatore, M. A., & Grundy, E. (2021). Area deprivation, perceived neighbourhood cohesion and mental health at older ages: A cross lagged analysis of UK longitudinal data. *Health Place, 67*, 102470. doi:10.1016/j.healthplace.2020.102470

Sarkar, C., Gallacher, J., & Webster, C. (2013). Urban built environment configuration and psychological distress in older men: Results from the Caerphilly study. *BMC Public Health, 13*(1). doi:10.1186/1471-2458-13-695

Schieman, S., Pearlin, L. I., & Meersman, S. C. (2006). Neighborhood disadvantage and anger among older adults: social comparisons as effect modifiers. *J Health Soc Behav, 47*(2), 156-172. doi:10.1177/002214650604700205

Sheffield, K. M., & Peek, M. K. (2009). Neighborhood context and cognitive decline in older Mexican Americans: results from the Hispanic Established Populations for Epidemiologic Studies of the Elderly. *Am J Epidemiol, 169*(9), 1092-1101. doi:10.1093/aje/kwp005

Shih, R. A., Ghosh-Dastidar, B., Margolis, K. L., Slaughter, M. E., Jewell, A., Bird, C. E., . . . Espeland, M. A. (2011). Neighborhood socioeconomic status and cognitive function in women. *Am J Public Health, 101*(9), 1721-1728. doi:10.2105/AJPH.2011.300169

Sisco, S. M., & Marsiske, M. (2012). Neighborhood Influences on Late Life Cognition in the ACTIVE Study. *J Aging Res, 2012*, 435826. doi:10.1155/2012/435826

Smith, R. J., Lehning, A. J., & Kim, K. (2018). Aging in Place in Gentrifying Neighborhoods: Implications for Physical and Mental Health. *Gerontologist, 58*(1), 26-35. doi:10.1093/geront/gnx105

Stroope, S., Cohen, I. F. A., Tom, J. C., Franzen, A. B., Valasik, M. A., & Markides, K. S. (2017). Neighborhood perception and self-rated health among Mexican American older adults. *Geriatr Gerontol Int, 17*(12), 2559-2564. doi:10.1111/ggi.13089

Stroope, S., Martinez, B. C., Eschbach, K., Peek, M. K., & Markides, K. S. (2015). Neighborhood Ethnic Composition and Problem Drinking Among Older Mexican American Men: Results from the Hispanic Established Populations for the Epidemiologic Study of the Elderly. *J Immigr Minor Health, 17*(4), 1055-1060. doi:10.1007/s10903-014-0033-8

Subramanian, S. V., Kubzansky, L., Berkman, L., Fay, M., & Kawachi, I. (2006). Neighborhood effects on the self-rated health of elders: uncovering the relative importance of structural and service-related neighborhood environments. *J Gerontol B Psychol Sci Soc Sci, 61*(3), S153-160. doi:10.1093/geronb/61.3.s153

Timmermans, E., Motoc, I., Noordzij, J. M., Beenackers, M. A., Wissa, R., Sarr, A., . . . Huisman, M. (2020). Social and physical neighbourhood characteristics and loneliness among older adults: results from the MINDMAP project. *J Epidemiol Community Health*. doi:10.1136/jech-2020-214217

Van Dyck, D., Barnett, A., Van Cauwenberg, J., Zhang, C. J. P., Sit, C. H. P., & Cerin, E. (2020). Main and interacting effects of physical activity and sedentary time on older adults' BMI: The moderating roles of socio-demographic and environmental attributes. *PLoS One, 15*(7), e0235833. doi:10.1371/journal.pone.0235833

Wagner, K. J., Boing, A. F., Subramanian, S. V., Höfelmann, D. A., & D'Orsi, E. (2016). Effects of neighborhood socioeconomic status on blood pressure in older adults. *Rev Saude Publica, 50*, 78. doi:10.1590/s1518-8787.2016050006595

Walters, K., Breeze, E., Wilkinson, P., Price, G. M., Bulpitt, C. J., & Fletcher, A. (2004). Local area deprivation and urban-rural differences in anxiety and depression among people older than 75 years in Britain. *Am J Public Health, 94*(10), 1768-1774. doi:10.2105/ajph.94.10.1768

Wee, L. E., Yeo, W. X., Yang, G. R., Hannan, N., Lim, K., Chua, C., . . . Shen, H. M. (2012). Individual and Area Level Socioeconomic Status and Its Association with Cognitive Function and Cognitive Impairment (Low MMSE) among Community-Dwelling Elderly in Singapore. *Dement Geriatr Cogn Dis Extra, 2*(1), 529-542. doi:10.1159/000345036

Wee, L. E., Yong, Y. Z., Chng, M. W., Chew, S. H., Cheng, L., Chua, Q. H., . . . Koh, G. C. (2014). Individual and area-level socioeconomic status and their association with depression amongst community-dwelling elderly in Singapore. *Aging Ment Health, 18*(5), 628-641. doi:10.1080/13607863.2013.866632

Weil, J. (2019). Relationship to Place for Older Adults in a New York City Neighborhood Undergoing Gentrification: A Discourse Analysis. *City & Community, 18*(4), 1267-1286. doi:10.1111/cico.12469

Wight, R. G., Aneshensel, C. S., Miller-Martinez, D., Botticello, A. L., Cummings, J. R., Karlamangla, A. S., & Seeman, T. E. (2006). Urban neighborhood context, educational attainment, and cognitive function among older adults. *Am J Epidemiol, 163*(12), 1071-1078. doi:10.1093/aje/kwj176

Wight, R. G., Cummings, J. R., Karlamangla, A. S., & Aneshensel, C. S. (2009). Urban neighborhood context and change in depressive symptoms in late life. *J Gerontol B Psychol Sci Soc Sci, 64*(2), 247-251. doi:10.1093/geronb/gbn016

Wight, R. G., Cummings, J. R., Karlamangla, A. S., & Aneshensel, C. S. (2010). Urban neighborhood context and mortality in late life. *J Aging Health, 22*(2), 197-218. doi:10.1177/0898264309355980

Wight, R. G., Cummings, J. R., Miller-Martinez, D., Karlamangla, A. S., Seeman, T. E., & Aneshensel, C. S. (2008). A multilevel analysis of urban neighborhood socioeconomic disadvantage and health in late life. *Soc Sci Med, 66*(4), 862-872. doi:10.1016/j.socscimed.2007.11.002

Wörn, J., Ellwardt, L., Aartsen, M., & Huisman, M. (2017). Cognitive functioning among Dutch older adults: Do neighborhood socioeconomic status and urbanity matter? *Soc Sci Med, 187*, 29-38. doi:10.1016/j.socscimed.2017.05.052

Wu, Y. T., Prina, A. M., Jones, A. P., Barnes, L. E., Matthews, F. E., & Brayne, C. (2015). Community environment, cognitive impairment and dementia in later life: results from the Cognitive Function and Ageing Study. *Age Ageing, 44*(6), 1005-1011. doi:10.1093/ageing/afv137

Yan, T., Escarce, J. J., Liang, L. J., Longstreth, W. T., Jr., Merkin, S. S., Ovbiagele, B., . . . Brown, A. F. (2013). Exploring psychosocial pathways between neighbourhood characteristics and stroke in older adults: the cardiovascular health study. *Age Ageing, 42*(3), 391-397. doi:10.1093/ageing/afs179

Yao, L., & Robert, S. A. (2008). The contributions of race, individual socioeconomic status, and Neighborhood socioeconomic context on the self-rated health trajectories and mortality of older adults. *Res Aging, 30*(2), 251-273. doi:10.1177/0164027507311155

Yao, L., & Robert, S. A. (2011). Examining the Racial Crossover in Mortality between African American and White Older Adults: A Multilevel Survival Analysis of Race, Individual Socioeconomic Status, and Neighborhood Socioeconomic Context. *J Aging Res, 2011*, 132073. doi:10.4061/2011/132073
