# Supplementary material for: Neighbourhood Socioeconomic Processes and Dynamics and Healthy Ageing: A Scoping Review
Source: Int J Environ Res Public Health. 2022 May 31;19(11):6745. doi: 10.3390/ijerph19116745 (PMC9180257; doi:10.3390/ijerph19116745)
Supplement: Supplementary file 1 [file ijerph-19-06745-s001.zip › Supplementary material - V - Neighbourhood measures.pdf]

Table S5: Indicators used to measure neighbourhood socioeconomic deprivation

Neighbourhood socioeconomic status/deprivation			
			Example(s)
One measure	Registered unemployment	(Behanova et al., 2015; Behanova et al., 2017)	• Neighbourhood-level unemployment and municipality registered unemployment
	Income	(S. C. Brown et al., 2018; Danielewicz, Wagner, d'Orsi, & Boing, 2016; Van Dyck et al., 2020; Wörn, Ellwardt, Aartsen, & Huisman, 2017)	• Household income<br>• Average net income per inhabitant
	The proportion of social welfare recipients	(Vogt et al., 2015)	• The number of people receiving social welfare divided by the number of all residents below 65 years of age
	Education	(Wagner, Boing, Subramanian, Höfelmann, & D'Orsi, 2016; Wight et al., 2006)	• Years of schooling of the head of the household<br>• The proportion of residents aged 25 years or older without a high school degree
	Poverty	(Cagney, Browning, Iveniuk, & English, 2014; Guo, Chan, Chang, Liu, & Yip, 2019; Joshi et al., 2017)	• The proportion of people living in the respondent's tract who were below the poverty line
	Tax income	(Kim et al., 2017)	• The average tax income of a district was equal to, below, or above the 50th percentile
	Percentage of adults aged 25 or older	(L. Yao & Robert, 2011)	• The percentage of adults aged 25 years or older
Multiple indicators	Education	(Almeida et al., 2012; Amuzu, Carson, Watt, Lawlor, & Ebrahim, 2009; Aneshensel et al., 2007; Annear, Cushman, & Gidlow, 2009; Annear,	• The percentage of adults 25 years of age or older who had completed high school<br>• The percentage of adults 25 years or older who had completed college

		Gidlow, & Cushman, 2009; Araújo et al., 2018; Balamurugan, Delongchamp, Bates, & Mehta, 2013; Beere, Keeling, & Jamieson, 2019; Bhardwaj, Amiri, Buchwald, & Amram, 2020; Braga, Macinko, Proietti, Cesar, & Lima-Costa, 2010; A. F. Brown et al., 2011; Buffel, Phillipson, & Scharf, 2013; Cadar et al., 2018; Casanova et al., 2020; Chamberlain et al., 2020; Corriere et al., 2014; Diez Roux, Borrell, Haan, Jackson, & Schultz, 2004; Everson-Rose et al., 2011; Fernández-Blázquez et al., 2020; Fox et al., 2011; Franse et al., 2017; Gale, Dennison, Cooper, & Sayer, 2011; Garcia et al., 2015; Garcia et al., 2016; Hawkesworth et al., 2018; Hazzouri et al., 2011; Huang, Meyer, & Jin, 2018; Jung, Kind, Robert, Buckingham, & DuGoff, 2018; Lang, Gibbs, Steel, & Melzer, 2008; Lang et al., 2009; Lang, Llewellyn, Langa, Wallace, Huppert, et al., 2008; Lang, Llewellyn, Langa, Wallace, & Melzer, 2008; Letellier et al., 2017; Lönn, Melander, Crump, & Sundquist, 2019; Meijer et al., 2012; Merkin et al., 2007; Meyer et al., 2017;	• Percentage of adults without secondary education

		Miao, Wu, & Sun, 2019; Michael, Nagel, Gold, & Hillier, 2014; Mooney et al., 2017; Moser et al., 2014; Mõttus, Gale, Starr, & Deary, 2012; Nordstrom, Diez Roux, Jackson, & Gardin, 2004; Nordstrom et al., 2007; Omariba, 2010; Powell et al., 2020; Ramsay et al., 2015; Reimers & Laflamme, 2007; Ribeiro, Pires, Carvalho, & Pina, 2015; Rosso et al., 2016; Salvatore & Grundy, 2021; Sarkar, Gallacher, & Webster, 2013; Sheffield & Peek, 2009; Shih et al., 2011; Sisco & Marsiske, 2012; Timmermans et al., 2020; Wee et al., 2012; Wee et al., 2014; Wight, Cummings, Karlamangla, & Aneshensel, 2009, 2010; Wight et al., 2008; Wu et al., 2015; Yan et al., 2013)	
	(Un)employment	(Almeida et al., 2012; Aneshensel et al., 2007; Annear, Cushman, et al., 2009; Annear, Gidlow, et al., 2009; Basta, Matthews, Chatfield, Brayne, & MRC-CFAS, 2007; Beere et al., 2019; Bhardwaj et al., 2020; Bowling & Stafford, 2007; Breeze et al., 2005; Brenner & Clarke, 2019; Buffel et al., 2013; Cadar et al., 2018; Casanova et al., 2020; Chamberlain et al., 2020;	• Percentage of unemployment<br>• Residents aged 16 or older who are unemployed

		Fernández-Blázquez et al., 2020; Fox et al., 2011; Gale et al., 2011; Garcia et al., 2015; Garcia et al., 2016; Hawkesworth et al., 2018; Hazzouri et al., 2011; Jung et al., 2018; Lang, Gibbs, et al., 2008; Lang et al., 2009; Lang, Llewellyn, Langa, Wallace, Huppert, et al., 2008; Lang, Llewellyn, Langa, Wallace, & Melzer, 2008; Lawlor, Davey Smith, Patel, & Ebrahim, 2005; Letellier et al., 2019; Letellier et al., 2017; Lönn et al., 2019; Meijer et al., 2012; Mooney et al., 2017; Mõttus et al., 2012; Powell et al., 2020; Ramsay et al., 2015; Reimers & Laflamme, 2007; Salvatore & Grundy, 2021; Sarkar et al., 2013; Shih et al., 2011; Stroope et al., 2017; Stroope, Martinez, Eschbach, Peek, & Markides, 2015; Walters et al., 2004; Wee et al., 2014; Wight et al., 2009, 2010; Wight et al., 2008; Wu et al., 2015; Li Yao & Robert, 2008)	
	Income/Wealth	(Almeida et al., 2012; Amuzu et al., 2009; Aneshensel et al., 2007; Annear, Cushman, et al., 2009; Annear, Gidlow, et al., 2009; Beere et al., 2019; Bhardwaj et al., 2020; A. F. Brown et al., 2011; Buffel et al., 2013; Cadar et al., 2018; Casanova et al.,	• Average of the household expenditures<br>• Median household income<br>• The percentage of households that have annual income above the median<br>• The proportion of households with incomes of $50,000 or more

		2020; Cerin et al., 2013; Chaix, Rosvall, & Merlo, 2007; Chamberlain et al., 2020; Corriere et al., 2014; Diez Roux et al., 2004; Espino, Lichtenstein, Palmer, & Hazuda, 2001; Espinoza & Hazuda, 2015; Everson-Rose et al., 2011; Fernández-Blázquez et al., 2020; Fox et al., 2011; Franse et al., 2017; Gale et al., 2011; Giehl, Hallal, Weber Corseuil, Schneider, & d'Orsi, 2016; Hawkesworth et al., 2018; Hybels et al., 2006; Kelley-Moore, Cagney, Skarupski, Everson-Rose, & Mendes de Leon, 2016; Letellier et al., 2019; Letellier et al., 2017; Lönn et al., 2019; Meijer et al., 2012; Menec, Shooshtari, Nowicki, & Fournier, 2010; Merkin et al., 2007; Meyer et al., 2017; Michael et al., 2014; Mooney et al., 2017; Mõttus et al., 2012; Nguyen, 2016; Nicklett et al., 2011; Nordstrom et al., 2004; Nordstrom et al., 2007; Powell et al., 2020; Ramsay et al., 2015; Reimers & Laflamme, 2007; Rosso et al., 2016; Salvatore & Grundy, 2021; Sarkar et al., 2013; Sheffield & Peek, 2009; Shih et al., 2011; Sisco & Marsiske, 2012; Stroope et al., 2017; Stroope et al., 2015;	

		Subramanian, Kubzansky, Berkman, Fay, & Kawachi, 2006; Timmermans et al., 2020; Wee et al., 2012; Wee et al., 2014; Wight et al., 2009, 2010; Wu et al., 2015; Yan et al., 2013; Li Yao & Robert, 2008)	
	Poverty	(Alaazi, Menon, Stafinski, Hodgins, & Jhangri, 2021; Aneshensel et al., 2007; Auchincloss, Van Nostrand, & Ronsaville, 2001; Balamurugan et al., 2013; Bhardwaj et al., 2020; Bolstad, Moak, Brown, Kennedy, & Buys, 2020; Buys et al., 2015; Chamberlain et al., 2020; Garcia et al., 2015; Garcia et al., 2016; Guo, Chang, et al., 2019; Hazzouri et al., 2011; Huang et al., 2018; Hybels et al., 2006; Jung et al., 2018; Ko, Jang, Park, Rhew, & Chiriboga, 2014; Kubzansky et al., 2005; Kwag, Jang, Rhew, & Chiriboga, 2011; Lo et al., 2016; Michael et al., 2014; Ostir, Eschbach, Markides, & Goodwin, 2003; Purser et al., 2008; Schieman, Pearlin, & Meersman, 2006; Sheffield & Peek, 2009; Shih et al., 2011; Stroope et al., 2017; Stroope et al., 2015; Subramanian et al., 2006; Wight et al., 2009; Wight et al., 2008; Li Yao & Robert, 2008)	• Median family income<br>• Per cent of residents of all ages living in poverty<br>• Per cent of residents aged 65 years and over and in poverty<br>• Percentage of households with income levels below the poverty line

	Household tenure/composition/density	(Almeida et al., 2012; Araújo et al., 2018; Auchincloss et al., 2001; Balamurugan et al., 2013; Basta et al., 2007; Beard et al., 2009; Bolstad et al., 2020; Bowling & Stafford, 2007; Breeze et al., 2005; Brenner & Clarke, 2019; Buffel et al., 2013; Buys et al., 2015; Casanova et al., 2020; Cerin et al., 2013; Everson-Rose et al., 2011; Garcia et al., 2015; Garcia et al., 2016; Hannon, Sawyer, & Allman, 2012; Hazzouri et al., 2011; Lawlor et al., 2005; Letellier et al., 2019; Letellier et al., 2017; Lo et al., 2016; Moser et al., 2014; Omariba, 2010; Pearce, Cherrie, Shortt, Deary, & Thompson, 2018; Reimers & Laflamme, 2007; Schieman et al., 2006; Shih et al., 2011; Walters et al., 2004; Wee et al., 2012; Wee et al., 2014; Li Yao & Robert, 2008)	• Percentage of households renting their accommodation<br>• Percentage of female-headed households<br>• Percentage of families that are composed of husband and wife
	Residential stability	(Guo, Chang, et al., 2019; Hybels et al., 2006; Kubzansky et al., 2005; Menec et al., 2010; Ostir et al., 2003; Subramanian et al., 2006; Wight et al., 2009, 2010)	• The proportion of people who had lived in the same house for the past 5 years
	Racial/ethnic heterogeneity/group	(Almeida et al., 2012; Balamurugan et al., 2013; Guo, Chang, et al., 2019; Huang et al., 2018; Hybels et al., 2006; Kwag et al.,	• The proportion of residents who are African American and/or the proportion of Hispanic residents

		2011; Omariba, 2010; Ostir et al., 2003; Patel, Eschbach, Rudkin, Peek, & Markides, 2003; Subramanian et al., 2006; Wight et al., 2009, 2010)	
	Health, health deprivation and disability	(Almeida et al., 2012; Bowling & Stafford, 2007; Cadar et al., 2018; Fox et al., 2011; Hawkesworth et al., 2018; Huang et al., 2018; Lang, Gibbs, et al., 2008; Lang et al., 2009; Lang, Llewellyn, Langa, Wallace, Huppert, et al., 2008; Lang, Llewellyn, Langa, Wallace, & Melzer, 2008; Meijer et al., 2012; Mõttus et al., 2012; Ramsay et al., 2015; Sarkar et al., 2013; Wu et al., 2015)	• The proportion of people under 70 years who have a long-term health condition or disability and need assistance<br>• Reported long-term illness
	Skills and training	(Almeida et al., 2012; Amuzu et al., 2009; Cadar et al., 2018; Gale et al., 2011; Lang, Gibbs, et al., 2008; Lang et al., 2009; Lang, Llewellyn, Langa, Wallace, Huppert, et al., 2008; Lang, Llewellyn, Langa, Wallace, & Melzer, 2008; Meijer et al., 2012; Mõttus et al., 2012; Ramsay et al., 2015)	• The proportion of people classified as low skill community and personal service workers
	Barriers to housing and services	(Alaazi et al., 2021; Auchincloss et al., 2001; Cadar et al., 2018; Espino et al., 2001; Espinoza & Hazuda, 2015; Fox et al., 2011; Gale et al., 2011; Lang, Gibbs, et al., 2008; Lang et al., 2009; Lang, Llewellyn, Langa,	• Availability of consumer goods, including food, clothing, housing, and medical care<br>• The difficulty of access to owner-occupation

		Wallace, Huppert, et al., 2008; Lang, Llewellyn, Langa, Wallace, & Melzer, 2008; Ramsay et al., 2015; Salvatore & Grundy, 2021; Sarkar et al., 2013; Wu et al., 2015)	
	Living environment	(Alaazi et al., 2021; Annear, Cushman, et al., 2009; Annear, Gidlow, et al., 2009; Araújo et al., 2018; Beard et al., 2009; Cadar et al., 2018; Espino et al., 2001; Espinoza & Hazuda, 2015; Fox et al., 2011; Gale et al., 2011; Lang, Gibbs, et al., 2008; Lang et al., 2009; Lang, Llewellyn, Langa, Wallace, Huppert, et al., 2008; Lang, Llewellyn, Langa, Wallace, & Melzer, 2008; Pearce et al., 2018; Ramsay et al., 2015; Salvatore & Grundy, 2021; Sarkar et al., 2013; Wu et al., 2015)	• Social and private housing in poor condition<br>• Houses without central heating<br>• Road traffic accidents involving injury to pedestrians and cyclists
	Crime or fear of crime	(Braga et al., 2010; Buffel et al., 2013; Cadar et al., 2018; Fox et al., 2011; Gale et al., 2011; Hawkesworth et al., 2018; Lang, Gibbs, et al., 2008; Lang et al., 2009; Lang, Llewellyn, Langa, Wallace, Huppert, et al., 2008; Lang, Llewellyn, Langa, Wallace, & Melzer, 2008; Mõttus et al., 2012;	• Burglary<br>• Theft<br>• Criminal damage<br>• Violence

		Ramsay et al., 2015; Timmermans et al., 2020)	
	Sense of belonging and trust in people	(Braga et al., 2010; Espino et al., 2001; Espinoza & Hazuda, 2015; Kelley-Moore et al., 2016)	
	Housing characteristics	(Alaazi et al., 2021; Amuzu et al., 2009; Bhardwaj et al., 2020; Bowling & Stafford, 2007; A. F. Brown et al., 2011; Chamberlain et al., 2020; Fernández-Blázquez et al., 2020; Hannon et al., 2012; Jung et al., 2018; Merkin et al., 2007; Michael et al., 2014; Mooney et al., 2017; Mõttus et al., 2012; Nguyen, 2016; Nicklett et al., 2011; Powell et al., 2020; Purser et al., 2008; Ribeiro et al., 2015; Rosso et al., 2016; Salvatore & Grundy, 2021; Sarkar et al., 2013; Sheffield & Peek, 2009; Timmermans et al., 2020; Wee et al., 2012; Wee et al., 2014)	• Percentage of households receiving interest, dividend, or net rental income<br>• The median value of housing units<br>• Percentage of unoccupied dwellings<br>• Percentage of housing units that are rentals<br>• No bathroom in house, no hot water in house, shared bedroom
	Demographic information	(Auchincloss et al., 2001; Bowling & Stafford, 2007; Braga et al., 2010; Ko et al., 2014; Kubzansky et al., 2005; Kwag et al., 2011; Letellier et al., 2017; Menec et al., 2010; Purser et al., 2008; Ribeiro et al.,	• Race, age and gender segregation<br>• Proportion of people aged 65 and older

		2015; Subramanian et al., 2006; Wight et al., 2009, 2010)	
	Occupation	(Bowling & Stafford, 2007; A. F. Brown et al., 2011; Corriere et al., 2014; Diez Roux et al., 2004; Franse et al., 2017; Lawlor et al., 2005; Merkin et al., 2007; Meyer et al., 2017; Miao et al., 2019; Michael et al., 2014; Moser et al., 2014; Nicklett et al., 2011; Nordstrom et al., 2004; Nordstrom et al., 2007; Purser et al., 2008; Ribeiro et al., 2015; Sheffield & Peek, 2009; Sisco & Marsiske, 2012; Yan et al., 2013; Li Yao & Robert, 2008)	• The percentage of employed persons in executive, managerial, or professional specialty occupations
	Households receiving public assistance	(Casanova et al., 2020; Everson-Rose et al., 2011; Lönn et al., 2019; Reimers & Laflamme, 2007; Schieman et al., 2006; Shih et al., 2011; Stroope et al., 2017; Stroope et al., 2015; Timmermans et al., 2020; Wee et al., 2012; Wee et al., 2014; Wight et al., 2009, 2010; Wight et al., 2008)	• Percentage of households receiving public assistance
	Not available	(Li et al., 2014)	• Indicators not specified

Alaazi, D. A., Menon, D., Stafinski, T., Hodgins, S., & Jhangri, G. (2021). Quality of life of older adults in two contrasting neighbourhoods in Accra, Ghana. *Social Science and Medicine, 270.* doi:10.1016/j.socscimed.2020.113659

Almeida, O. P., Pirkis, J., Kerse, N., Sim, M., Flicker, L., Snowdon, J., . . . Pfaff, J. J. (2012). Socioeconomic disadvantage increases risk of prevalent and persistent depression in later life. *J Affect Disord, 138*(3), 322-331. doi:10.1016/j.jad.2012.01.021

Amuzu, A., Carson, C., Watt, H. C., Lawlor, D. A., & Ebrahim, S. (2009). Influence of area and individual lifecourse deprivation on health behaviours: findings from the British Women's Heart and Health Study. *European journal of cardiovascular prevention and rehabilitation, 16*(2), 169-173. doi:10.1097/HJR.0b013e328325d64d

Aneshensel, C. S., Wight, R. G., Miller-Martinez, D., Botticello, A. L., Karlamangla, A. S., & Seeman, T. E. (2007). Urban neighborhoods and depressive symptoms among older adults. *J Gerontol B Psychol Sci Soc Sci, 62*(1), S52-59. doi:10.1093/geronb/62.1.s52

Annear, M. J., Cushman, G., & Gidlow, B. (2009). Leisure time physical activity differences among older adults from diverse socioeconomic neighborhoods. *Health Place, 15*(2), 482-490. doi:10.1016/j.healthplace.2008.09.005

Annear, M. J., Gidlow, B., & Cushman, G. (2009). Neighbourhood deprivation and older adults' preferences for and perceptions of active leisure participation. *Annals of Leisure Research, 12*(2), 96-128. doi:10.1080/11745398.2009.9686814

Araújo, C. A. H., Giehl, M. W. C., Danielewicz, A. L., Araujo, P. G., d'Orsi, E., & Boing, A. F. (2018). Built environment, contextual income, and obesity in older adults: evidence from a population-based study. *Cad Saude Publica, 34*(5), e00060217. doi:10.1590/0102-311x00060217

Auchincloss, A. H., Van Nostrand, J. F., & Ronsaville, D. (2001). Access to health care for older persons in the United States: personal, structural, and neighborhood characteristics. *J Aging Health, 13*(3), 329-354. doi:10.1177/089826430101300302

Balamurugan, A., Delongchamp, R., Bates, J. H., & Mehta, J. L. (2013). The neighborhood where you live is a risk factor for stroke. *Circulation: Cardiovascular Quality and Outcomes, 6*(6), 668-673. doi:10.1161/CIRCOUTCOMES.113.000265

Basta, N. E., Matthews, F. E., Chatfield, M. D., Brayne, C., & MRC-CFAS. (2007). Community-level socio-economic status and cognitive and functional impairment in the older population. *Eur J Public Health, 18*(1), 48-54. doi:10.1093/eurpub/ckm076

Beard, J. R., Blaney, S., Cerda, M., Frye, V., Lovasi, G. S., Ompad, D., . . . Vlahov, D. (2009). Neighborhood characteristics and disability in older adults. *J Gerontol B Psychol Sci Soc Sci, 64*(2), 252-257. doi:10.1093/geronb/gbn018

Beere, P., Keeling, S., & Jamieson, H. (2019). Ageing, loneliness, and the geographic distribution of New Zealand's interRAI-HC cohort. *Soc Sci Med, 227*, 84-92. doi:10.1016/j.socscimed.2018.08.002

Behanova, M., Katreniakova, Z., Nagyova, I., van Ameijden, E. J., Dijkshoorn, H., van Dijk, J. P., & Reijneveld, S. A. (2015). The effect of neighbourhood unemployment on health-risk behaviours in elderly differs between Slovak and Dutch cities. *Eur J Public Health, 25*(1), 108-114. doi:10.1093/eurpub/cku116

Behanova, M., Katreniakova, Z., Nagyova, I., van Ameijden, E. J. C., van Dijk, J. P., & Reijneveld, S. A. (2017). Elderly from lower socioeconomic groups are more vulnerable to mental health problems, but area deprivation does not contribute: a comparison between Slovak and Dutch cities. *Eur J Public Health, 27*(suppl_2), 80-85. doi:10.1093/eurpub/ckv096

Bhardwaj, R., Amiri, S., Buchwald, D., & Amram, O. (2020). Environmental Correlates of Reaching a Centenarian Age: Analysis of 144,665 Deaths in Washington State for 2011-2015. *Int J Environ Res Public Health, 17*(8). doi:10.3390/ijerph17082828

Bolstad, C. J., Moak, R., Brown, C. J., Kennedy, R. E., & Buys, D. R. (2020). Neighborhood Disadvantage Is Associated with Depressive Symptoms but Not Depression Diagnosis in Older Adults. *Int J Environ Res Public Health, 17*(16). doi:10.3390/ijerph17165745

Bowling, A., & Stafford, M. (2007). How do objective and subjective assessments of neighbourhood influence social and physical functioning in older age? Findings from a British survey of ageing. *Soc Sci Med, 64*(12), 2533-2549. doi:10.1016/j.socscimed.2007.03.009

Braga, L. d. S., Macinko, J., Proietti, F. A., Cesar, C. C., & Lima-Costa, M. F. (2010). Intra-urban differences in vulnerability among the elderly population. *Cad Saude Publica, 26*(12), 2306-2314.

Breeze, E., Jones, D. A., Wilkinson, P., Bulpitt, C. J., Grundy, C., Latif, A. M., & Fletcher, A. E. (2005). Area deprivation, social class, and quality of life among people aged 75 years and over in Britain. *Int J Epidemiol, 34*(2), 276-283. doi:10.1093/ije/dyh328

Brenner, A. B., & Clarke, P. J. (2019). Difficulty and independence in shopping among older Americans: more than just leaving the house. *Disabil Rehabil, 41*(2), 191-200. doi:10.1080/09638288.2017.1398785

Brown, A. F., Liang, L. J., Vassar, S. D., Stein-Merkin, S., Longstreth, W. T., Jr., Ovbiagele, B., . . . Escarce, J. J. (2011). Neighborhood disadvantage and ischemic stroke: the Cardiovascular Health Study (CHS). *Stroke, 42*(12), 3363-3368. doi:10.1161/strokeaha.111.622134

Brown, S. C., Perrino, T., Lombard, J., Wang, K., Toro, M., Rundek, T., . . . Szapocznik, J. (2018). Health Disparities in the Relationship of Neighborhood Greenness to Mental Health Outcomes in 249,405 US Medicare Beneficiaries. *Int J Environ Res Public Health, 15*(3). doi:10.3390/ijerph15030430

Buffel, T., Phillipson, C., & Scharf, T. (2013). Experiences of neighbourhood exclusion and inclusion among older people living in deprived inner-city areas in Belgium and England. *Ageing & Society, 33*, 89-109. doi:10.1017/s0144686x12000542

Buys, D. R., Howard, V. J., McClure, L. A., Buys, K. C., Sawyer, P., Allman, R. M., & Levitan, E. B. (2015). Association between neighborhood disadvantage and hypertension prevalence, awareness, treatment, and control in older adults: results from the University of Alabama at Birmingham Study of Aging. *Am J Public Health, 105*(6), 1181-1188. doi:10.2105/ajph.2014.302048

Cadar, D., Lassale, C., Davies, H., Llewellyn, D. J., Batty, G. D., & Steptoe, A. (2018). Individual and Area-Based Socioeconomic Factors Associated With Dementia Incidence in England: Evidence From a 12-Year Follow-up in the English Longitudinal Study of Ageing. *JAMA Psychiatry, 75*(7), 723-732. doi:10.1001/jamapsychiatry.2018.1012

Cagney, K. A., Browning, C. R., Iveniuk, J., & English, N. (2014). The onset of depression during the great recession: foreclosure and older adult mental health. *Am J Public Health, 104*(3), 498-505. doi:10.2105/ajph.2013.301566

Casanova, R., Saldana, S., Lutz, M. W., Plassman, B. L., Kuchibhatla, M., & Hayden, K. M. (2020). Investigating predictors of cognitive decline using machine learning. *Journals of Gerontology - Series B Psychological Sciences and Social Sciences, 75*(4), 733-742. doi:10.1093/geronb/gby054

Cerin, E., Mellecker, R., Macfarlane, D. J., Barnett, A., Cheung, M. C., Sit, C. H., & Chan, W. M. (2013). Socioeconomic status, neighborhood characteristics, and walking within the neighborhood among older Hong Kong Chinese. *J Aging Health, 25*(8), 1425-1444. doi:10.1177/0898264313510034

Chaix, B., Rosvall, M., & Merlo, J. (2007). Assessment of the magnitude of geographical variations and socioeconomic contextual effects on ischaemic heart disease mortality: a multilevel survival analysis of a large Swedish cohort. *J Epidemiol Community Health, 61*(4), 349-355. doi:10.1136/jech.2006.047597

Chamberlain, A. M., Finney Rutten, L. J., Wilson, P. M., Fan, C., Boyd, C. M., Jacobson, D. J., . . . St Sauver, J. L. (2020). Neighborhood socioeconomic disadvantage is associated with multimorbidity in a geographically-defined community. *BMC Public Health, 20*(1). doi:10.1186/s12889-019-8123-0

Corriere, M. D., Yao, W., Xue, Q. L., Cappola, A. R., Fried, L. P., Thorpe, R. J., Jr., . . . Kalyani, R. R. (2014). The association of neighborhood characteristics with obesity and metabolic conditions in older women. *J Nutr Health Aging, 18*(9), 792-798. doi:10.1007/s12603-014-0477-5

Danielewicz, A. L., Wagner, K. J., d'Orsi, E., & Boing, A. F. (2016). Is cognitive decline in the elderly associated with contextual income? Results of a population-based study in southern Brazil. *Cad Saude Publica, 32*(5), e00112715. doi:10.1590/0102-311x00112715

Diez Roux, A. V., Borrell, L. N., Haan, M., Jackson, S. A., & Schultz, R. (2004). Neighbourhood environments and mortality in an elderly cohort: results from the cardiovascular health study. *J Epidemiol Community Health, 58*(11), 917-923. doi:10.1136/jech.2003.019596

Espino, D. V., Lichtenstein, M. J., Palmer, R. F., & Hazuda, H. P. (2001). Ethnic differences in Mini-Mental State Examination (MMSE) scores: Where you live makes a difference. *J Am Geriatr Soc, 49*(5), 538-548. doi:10.1046/j.1532-5415.2001.49111.x

Espinoza, S. E., & Hazuda, H. P. (2015). Frailty prevalence and neighborhood residence in older Mexican Americans: the San Antonio longitudinal study of aging. *J Am Geriatr Soc, 63*(1), 106-111. doi:10.1111/jgs.13202

Everson-Rose, S. A., Skarupski, K. A., Barnes, L. L., Beck, T., Evans, D. A., & Mendes de Leon, C. F. (2011). Neighborhood socioeconomic conditions are associated with psychosocial functioning in older black and white adults. *Health Place, 17*(3), 793-800. doi:10.1016/j.healthplace.2011.02.007

Fernández-Blázquez, M. A., Noriega-Ruiz, B., Ávila-Villanueva, M., Valentí-Soler, M., Frades-Payo, B., Del Ser, T., & Gómez-Ramírez, J. (2020). Impact of individual and neighborhood dimensions of socioeconomic status on the prevalence of mild cognitive impairment over seven-year follow-up. *Aging Ment Health*, 1-10. doi:10.1080/13607863.2020.1725803

Fox, K. R., Hillsdon, M., Sharp, D., Cooper, A. R., Coulson, J. C., Davis, M., . . . Thompson, J. L. (2011). Neighbourhood deprivation and physical activity in UK older adults. *Health Place, 17*(2), 633-640. doi:10.1016/j.healthplace.2011.01.002

Franse, C. B., van Grieken, A., Qin, L., Melis, R. J. F., Rietjens, J. A. C., & Raat, H. (2017). Socioeconomic inequalities in frailty and frailty components among community-dwelling older citizens. *PLoS One, 12*(11), e0187946. doi:10.1371/journal.pone.0187946

Gale, C. R., Dennison, E. M., Cooper, C., & Sayer, A. A. (2011). Neighbourhood environment and positive mental health in older people: the Hertfordshire Cohort Study. *Health Place, 17*(4), 867-874. doi:10.1016/j.healthplace.2011.05.003

Garcia, L., Lee, A., Zeki Al Hazzouri, A., Neuhaus, J., Epstein, M., & Haan, M. (2015). The Impact of Neighborhood Socioeconomic Position on Prevalence of Diabetes and Prediabetes in Older Latinos: The Sacramento Area Latino Study on Aging. *Hisp Health Care Int, 13*(2), 77-85. doi:10.1891/1540-4153.13.2.77

Garcia, L., Lee, A., Zeki Al Hazzouri, A., Neuhaus, J. M., Moyce, S., Aiello, A., . . . Haan, M. N. (2016). Influence of neighbourhood socioeconomic position on the transition to type II diabetes in older Mexican Americans: the Sacramento Area Longitudinal Study on Aging. *BMJ Open, 6*(8), e010905. doi:10.1136/bmjopen-2015-010905

Giehl, M. C. G., Hallal, P. C., Weber Corseuil, C., Schneider, I. J., & d'Orsi, E. (2016). Built Environment and Walking Behavior Among Brazilian Older Adults: A Population-Based Study. *J Phys Act Health, 13*(6), 617-624. doi:10.1123/jpah.2015-0355

Guo, Y., Chan, C. H., Chang, Q., Liu, T., & Yip, P. S. F. (2019). Neighborhood environment and cognitive function in older adults: A multilevel analysis in Hong Kong. *Health Place, 58*, 102146. doi:10.1016/j.healthplace.2019.102146

Guo, Y., Chang, S. S., Chan, C. H., Chang, Q., Hsu, C. Y., & Yip, P. S. F. (2019). Association of neighbourhood social and physical attributes with depression in older adults in Hong Kong: a multilevel analysis. *J Epidemiol Community Health, 74*(2), 120-129. doi:10.1136/jech-2019-212977

Hannon, L., 3rd, Sawyer, P., & Allman, R. M. (2012). Housing, the Neighborhood Environment, and Physical Activity among Older African Americans. *J Health Dispar Res Pract, 5*(3), 27-41.

Hawkesworth, S., Silverwood, R. J., Armstrong, B., Pliakas, T., Nanchalal, K., Jefferis, B. J., . . . Lock, K. (2018). Investigating associations between the built environment and physical activity among older people in 20 UK towns. *J Epidemiol Community Health, 72*(2), 121-131. doi:10.1136/jech-2017-209440

Hazzouri, A. Z. A., Haan, M. N., Osypuk, T., Abdou, C., Hinton, L., & Aiello, A. E. (2011). Neighborhood socioeconomic context and cognitive decline among older Mexican Americans: results from the Sacramento Area Latino Study on Aging. *Am J Epidemiol, 174*(4), 423-431. doi:10.1093/aje/kwr095

Huang, Y., Meyer, P., & Jin, L. (2018). Neighborhood socioeconomic characteristics, healthcare spatial access, and emergency department visits for ambulatory care sensitive conditions for elderly. *Prev Med Rep, 12*, 101-105. doi:10.1016/j.pmedr.2018.08.015

Hybels, C. F., Blazer, D. G., Pieper, C. F., Burchett, B. M., Hays, J. C., Fillenbaum, G. G., . . . Berkman, L. F. (2006). Sociodemographic characteristics of the neighborhood and depressive symptoms in older adults: using multilevel modeling in geriatric psychiatry. *Am J Geriatr Psychiatry, 14*(6), 498-506. doi:10.1097/01.Jgp.0000194649.49784.29

Joshi, S., Mooney, S. J., Rundle, A. G., Quinn, J. W., Beard, J. R., & Cerdá, M. (2017). Pathways from neighborhood poverty to depression among older adults. *Health Place, 43*, 138-143. doi:10.1016/j.healthplace.2016.12.003

Jung, D., Kind, A., Robert, S., Buckingham, W., & DuGoff, E. (2018). Linking Neighborhood Context and Health in Community-Dwelling Older Adults in the Medicare Advantage Program. *J Am Geriatr Soc, 66*(6), 1158-1164. doi:10.1111/jgs.15366

Kelley-Moore, J. A., Cagney, K. A., Skarupski, K. A., Everson-Rose, S. A., & Mendes de Leon, C. F. (2016). Do Local Social Hierarchies Matter for Mental Health? A Study of Neighborhood Social Status and Depressive Symptoms in Older Adults. *J Gerontol B Psychol Sci Soc Sci, 71*(2), 369-377. doi:10.1093/geronb/gbv047

Kim, G. H., Lee, H. A., Park, H., Lee, D. Y., Jo, I., Choi, S. H., . . . Jeong, J. H. (2017). Effect of Individual and District-level Socioeconomic Disparities on Cognitive Decline in Community-dwelling Elderly in Seoul. *J Korean Med Sci, 32*(9), 1508-1515. doi:10.3346/jkms.2017.32.9.1508

Ko, J. E., Jang, Y., Park, N. S., Rhew, S. H., & Chiriboga, D. A. (2014). Neighborhood effects on the self-rated health of older adults from four racial/ethnic groups. *Soc Work Public Health, 29*(2), 89-99. doi:10.1080/19371918.2013.829760

Kubzansky, L. D., Subramanian, S. V., Kawachi, I., Fay, M. E., Soobader, M. J., & Berkman, L. F. (2005). Neighborhood contextual influences on depressive symptoms in the elderly. *Am J Epidemiol, 162*(3), 253-260. doi:10.1093/aje/kwi185

Kwag, K. H., Jang, Y., Rhew, S. H., & Chiriboga, D. A. (2011). Neighborhood Effects on Physical and Mental Health: A Study of Korean American Older Adults. *Asian Am J Psychol, 2*(2), 91-100. doi:10.1037/a0023656

Lang, I. A., Gibbs, S. J., Steel, N., & Melzer, D. (2008). Neighbourhood deprivation and dental service use: a cross-sectional analysis of older people in England. *J Public Health (Oxf), 30*(4), 472-478. doi:10.1093/pubmed/fdn047

Lang, I. A., Hubbard, R. E., Andrew, M. K., Llewellyn, D. J., Melzer, D., & Rockwood, K. (2009). Neighborhood deprivation, individual socioeconomic status, and frailty in older adults. *J Am Geriatr Soc, 57*(10), 1776-1780. doi:10.1111/j.1532-5415.2009.02480.x

Lang, I. A., Llewellyn, D. J., Langa, K. M., Wallace, R. B., Huppert, F. A., & Melzer, D. (2008). Neighborhood deprivation, individual socioeconomic status, and cognitive function in older people: analyses from the English Longitudinal Study of Ageing. *J Am Geriatr Soc, 56*(2), 191-198. doi:10.1111/j.1532-5415.2007.01557.x

Lang, I. A., Llewellyn, D. J., Langa, K. M., Wallace, R. B., & Melzer, D. (2008). Neighbourhood deprivation and incident mobility disability in older adults. *Age Ageing, 37*(4), 403-410. doi:10.1093/ageing/afn092

Lawlor, D. A., Davey Smith, G., Patel, R., & Ebrahim, S. (2005). Life-course socioeconomic position, area deprivation, and coronary heart disease: findings from the British Women's Heart and Health Study. *Am J Public Health, 95*(1), 91-97. doi:10.2105/AJPH.2003.035592

Letellier, N., Carrière, I., Gutierrez, L. A., Gabelle, A., Dartigues, J. F., Dufouil, C., . . . Berr, C. (2019). Influence of activity space on the association between neighborhood characteristics and dementia risk: results from the 3-City study cohort. *BMC Geriatr, 19*(1), 4. doi:10.1186/s12877-018-1017-7

Letellier, N., Gutierrez, L. A., Carrière, I., Gabelle, A., Dartigues, J. F., Dufouil, C., . . . Berr, C. (2017). Sex-specific association between neighborhood characteristics and dementia: The Three-City cohort. *Alzheimers Dement, 14*(4), 473-482. doi:10.1016/j.jalz.2017.09.015

Li, W., Procter-Gray, E., Lipsitz, L. A., Leveille, S. G., Hackman, H., Biondolillo, M., & Hannan, M. T. (2014). Utilitarian walking, neighborhood environment, and risk of outdoor falls among older adults. *Am J Public Health, 104*(9), e30-37. doi:10.2105/ajph.2014.302104

Lo, A. X., Rundle, A. G., Buys, D., Kennedy, R. E., Sawyer, P., Allman, R. M., & Brown, C. J. (2016). Neighborhood Disadvantage and Life-Space Mobility Are Associated with Incident Falls in Community-Dwelling Older Adults. *J Am Geriatr Soc, 64*(11), 2218-2225. doi:10.1111/jgs.14353

Lönn, S. L., Melander, O., Crump, C., & Sundquist, K. (2019). Accumulated neighbourhood deprivation and coronary heart disease: a nationwide cohort study from Sweden. *BMJ Open, 9*(9), e029248. doi:10.1136/bmjopen-2019-029248

Meijer, M., Kejs, A. M., Stock, C., Bloomfield, K., Ejstrud, B., & Schlattmann, P. (2012). Population density, socioeconomic environment and all-cause mortality: a multilevel survival analysis of 2.7 million individuals in Denmark. *Health Place, 18*(2), 391-399. doi:10.1016/j.healthplace.2011.12.001

Menec, V. H., Shooshtari, S., Nowicki, S., & Fournier, S. (2010). Does the relationship between neighborhood socioeconomic status and health outcomes persist into very old age? A population-based study. *J Aging Health, 22*(1), 27-47. doi:10.1177/0898264309349029

Merkin, S. S., Diez Roux, A. V., Coresh, J., Fried, L. F., Jackson, S. A., & Powe, N. R. (2007). Individual and neighborhood socioeconomic status and progressive chronic kidney disease in an elderly population: The Cardiovascular Health Study. *Soc Sci Med, 65*(4), 809-821. doi:10.1016/j.socscimed.2007.04.011

Meyer, O. L., Sisco, S. M., Harvey, D., Zahodne, L. B., Glymour, M. M., Manly, J. J., & Marsiske, M. (2017). Neighborhood Predictors of Cognitive Training Outcomes and Trajectories in ACTIVE. *Res Aging, 39*(3), 443-467. doi:10.1177/0164027515618242

Miao, J., Wu, X., & Sun, X. (2019). Neighborhood, social cohesion, and the Elderly's depression in Shanghai. *Soc Sci Med, 229*, 134-143. doi:10.1016/j.socscimed.2018.08.022

Michael, Y. L., Nagel, C. L., Gold, R., & Hillier, T. A. (2014). Does change in the neighborhood environment prevent obesity in older women? *Soc Sci Med, 102*, 129-137. doi:10.1016/j.socscimed.2013.11.047

Mooney, S. J., Joshi, S., Cerdá, M., Kennedy, G. J., Beard, J. R., & Rundle, A. G. (2017). Contextual Correlates of Physical Activity among Older Adults: A Neighborhood Environment-Wide Association Study (NE-WAS). *Cancer Epidemiol Biomarkers Prev, 26*(4), 495-504. doi:10.1158/1055-9965.Epi-16-0827

Moser, A., Panczak, R., Zwahlen, M., Clough-Gorr, K. M., Spoerri, A., Stuck, A. E., & Egger, M. (2014). What does your neighbourhood say about you? A study of life expectancy in 1.3 million Swiss neighbourhoods. *J Epidemiol Community Health, 68*(12), 1125-1132. doi:10.1136/jech-2014-204352

Mõttus, R., Gale, C. R., Starr, J. M., & Deary, I. J. (2012). 'On the street where you live': Neighbourhood deprivation and quality of life among community-dwelling older people in Edinburgh, Scotland. *Soc Sci Med, 74*(9), 1368-1374. doi:10.1016/j.socscimed.2011.12.050

Nguyen, H. V. (2016). Keeping Up with the Joneses: Neighbourhood Wealth and Hypertension. *Journal of Happiness Studies, 17*(3), 1255-1271. doi:10.1007/s10902-015-9641-9

Nicklett, E. J., Szanton, S., Sun, K., Ferrucci, L., Fried, L. P., Guralnik, J. M., & Semba, R. D. (2011). Neighborhood socioeconomic status is associated with serum carotenoid concentrations in older, community-dwelling women. *J Nutr, 141*(2), 284-289. doi:10.3945/jn.110.129684

Nordstrom, C. K., Diez Roux, A. V., Jackson, S. A., & Gardin, J. M. (2004). The association of personal and neighborhood socioeconomic indicators with subclinical cardiovascular disease in an elderly cohort. The cardiovascular health study. *Soc Sci Med, 59*(10), 2139-2147. doi:10.1016/j.socscimed.2004.03.017

Nordstrom, C. K., Diez Roux, A. V., Schulz, R., Haan, M. N., Jackson, S. A., & Balfour, J. L. (2007). Socioeconomic position and incident mobility impairment in the Cardiovascular Health Study. *BMC Geriatr, 7*, 11. doi:10.1186/1471-2318-7-11

Omariba, W. R. (2010). Neighbourhood characteristics, individual attributes and self-rated health among older Canadians. *Health and Place, 16*(5), 986-995. doi:10.1016/j.healthplace.2010.06.003

Ostir, G. V., Eschbach, K., Markides, K. S., & Goodwin, J. S. (2003). Neighbourhood composition and depressive symptoms among older Mexican Americans. *J Epidemiol Community Health, 57*(12), 987-992. doi:10.1136/jech.57.12.987

Patel, K. V., Eschbach, K., Rudkin, L. L., Peek, M. K., & Markides, K. S. (2003). Neighborhood context and self-rated health in older Mexican Americans. *Ann Epidemiol, 13*(9), 620-628. doi:10.1016/S1047-2797(03)00060-7

Pearce, J., Cherrie, M., Shortt, N., Deary, I., & Thompson, C. W. (2018). Life course of place: A longitudinal study of mental health and place. *Transactions of the Institute of British Geographers, 43*(4), 555-572. doi:10.1111/tran.12246

Powell, W. R., Buckingham, W. R., Larson, J. L., Vilen, L., Yu, M., Salamat, M. S., . . . Kind, A. J. H. (2020). Association of Neighborhood-Level Disadvantage With Alzheimer Disease Neuropathology. *JAMA Netw Open, 3*(6), e207559. doi:10.1001/jamanetworkopen.2020.7559

Purser, J. L., Kuchibhatla, M. N., Miranda, M. L., Blazer, D. G., Cohen, H. J., & Fillenbaum, G. G. (2008). Geographical segregation and IL-6: a marker of chronic inflammation in older adults. *Biomark Med, 2*(4), 335-348. doi:10.2217/17520363.2.4.335

Ramsay, S. E., Morris, R. W., Whincup, P. H., Subramanian, S. V., Papacosta, A. O., Lennon, L. T., & Wannamethee, S. G. (2015). The influence of neighbourhood-level socioeconomic deprivation on cardiovascular disease mortality in older age: longitudinal multilevel analyses from a cohort of older British men. *J Epidemiol Community Health, 69*(12), 1224-1231. doi:10.1136/jech-2015-205542

Reimers, A., & Laflamme, L. (2007). Hip fractures among the elderly: personal and contextual social factors that matter. *J Trauma, 62*(2), 365-369. doi:10.1097/01.ta.0000221669.26191.59

Ribeiro, A. I., Pires, A., Carvalho, M. S., & Pina, M. F. (2015). Distance to parks and non-residential destinations influences physical activity of older people, but crime doesn't: a cross-sectional study in a southern European city. *BMC Public Health, 15*, 593. doi:10.1186/s12889-015-1879-y

Rosso, A. L., Flatt, J. D., Carlson, M. C., Lovasi, G. S., Rosano, C., Brown, A. F., . . . Gianaros, P. J. (2016). Neighborhood Socioeconomic Status and Cognitive Function in Late Life. *Am J Epidemiol, 183*(12), 1088-1097. doi:10.1093/aje/kwv337

Salvatore, M. A., & Grundy, E. (2021). Area deprivation, perceived neighbourhood cohesion and mental health at older ages: A cross lagged analysis of UK longitudinal data. *Health Place, 67*, 102470. doi:10.1016/j.healthplace.2020.102470

Sarkar, C., Gallacher, J., & Webster, C. (2013). Urban built environment configuration and psychological distress in older men: Results from the Caerphilly study. *BMC Public Health, 13*(1). doi:10.1186/1471-2458-13-695

Schieman, S., Pearlin, L. I., & Meersman, S. C. (2006). Neighborhood disadvantage and anger among older adults: social comparisons as effect modifiers. *J Health Soc Behav, 47*(2), 156-172. doi:10.1177/002214650604700205

Sheffield, K. M., & Peek, M. K. (2009). Neighborhood context and cognitive decline in older Mexican Americans: results from the Hispanic Established Populations for Epidemiologic Studies of the Elderly. *Am J Epidemiol, 169*(9), 1092-1101. doi:10.1093/aje/kwp005

Shih, R. A., Ghosh-Dastidar, B., Margolis, K. L., Slaughter, M. E., Jewell, A., Bird, C. E., . . . Espeland, M. A. (2011). Neighborhood socioeconomic status and cognitive function in women. *Am J Public Health, 101*(9), 1721-1728. doi:10.2105/AJPH.2011.300169

Sisco, S. M., & Marsiske, M. (2012). Neighborhood Influences on Late Life Cognition in the ACTIVE Study. *J Aging Res, 2012*, 435826. doi:10.1155/2012/435826

Stroope, S., Cohen, I. F. A., Tom, J. C., Franzen, A. B., Valasik, M. A., & Markides, K. S. (2017). Neighborhood perception and self-rated health among Mexican American older adults. *Geriatr Gerontol Int, 17*(12), 2559-2564. doi:10.1111/ggi.13089

Stroope, S., Martinez, B. C., Eschbach, K., Peek, M. K., & Markides, K. S. (2015). Neighborhood Ethnic Composition and Problem Drinking Among Older Mexican American Men: Results from the Hispanic Established Populations for the Epidemiologic Study of the Elderly. *J Immigr Minor Health, 17*(4), 1055-1060. doi:10.1007/s10903-014-0033-8

Subramanian, S. V., Kubzansky, L., Berkman, L., Fay, M., & Kawachi, I. (2006). Neighborhood effects on the self-rated health of elders: uncovering the relative importance of structural and service-related neighborhood environments. *J Gerontol B Psychol Sci Soc Sci, 61*(3), S153-160. doi:10.1093/geronb/61.3.s153

Timmermans, E., Motoc, I., Noordzij, J. M., Beenackers, M. A., Wissa, R., Sarr, A., . . . Huisman, M. (2020). Social and physical neighbourhood characteristics and loneliness among older adults: results from the MINDMAP project. *J Epidemiol Community Health*. doi:10.1136/jech-2020-214217

Van Dyck, D., Barnett, A., Van Cauwenberg, J., Zhang, C. J. P., Sit, C. H. P., & Cerin, E. (2020). Main and interacting effects of physical activity and sedentary time on older adults' BMI: The moderating roles of socio-demographic and environmental attributes. *PLoS One, 15*(7), e0235833. doi:10.1371/journal.pone.0235833

Vogt, S., Mielck, A., Berger, U., Grill, E., Peters, A., Döring, A., . . . Maier, W. (2015). Neighborhood and healthy aging in a German city: distances to green space and senior service centers and their associations with physical constitution, disability, and health-related quality of life. *Eur J Ageing, 12*(4), 273-283. doi:10.1007/s10433-015-0345-0

Wagner, K. J., Boing, A. F., Subramanian, S. V., Höfelmann, D. A., & D'Orsi, E. (2016). Effects of neighborhood socioeconomic status on blood pressure in older adults. *Rev Saude Publica, 50*, 78. doi:10.1590/s1518-8787.2016050006595

Walters, K., Breeze, E., Wilkinson, P., Price, G. M., Bulpitt, C. J., & Fletcher, A. (2004). Local area deprivation and urban-rural differences in anxiety and depression among people older than 75 years in Britain. *Am J Public Health, 94*(10), 1768-1774. doi:10.2105/ajph.94.10.1768

Wee, L. E., Yeo, W. X., Yang, G. R., Hannan, N., Lim, K., Chua, C., . . . Shen, H. M. (2012). Individual and Area Level Socioeconomic Status and Its Association with Cognitive Function and Cognitive Impairment (Low MMSE) among Community-Dwelling Elderly in Singapore. *Dement Geriatr Cogn Dis Extra, 2*(1), 529-542. doi:10.1159/000345036

Wee, L. E., Yong, Y. Z., Chng, M. W., Chew, S. H., Cheng, L., Chua, Q. H., . . . Koh, G. C. (2014). Individual and area-level socioeconomic status and their association with depression amongst community-dwelling elderly in Singapore. *Aging Ment Health, 18*(5), 628-641. doi:10.1080/13607863.2013.866632

Wight, R. G., Aneshensel, C. S., Miller-Martinez, D., Botticello, A. L., Cummings, J. R., Karlamangla, A. S., & Seeman, T. E. (2006). Urban neighborhood context, educational attainment, and cognitive function among older adults. *Am J Epidemiol, 163*(12), 1071-1078. doi:10.1093/aje/kwj176

Wight, R. G., Cummings, J. R., Karlamangla, A. S., & Aneshensel, C. S. (2009). Urban neighborhood context and change in depressive symptoms in late life. *J Gerontol B Psychol Sci Soc Sci, 64*(2), 247-251. doi:10.1093/geronb/gbn016

Wight, R. G., Cummings, J. R., Karlamangla, A. S., & Aneshensel, C. S. (2010). Urban neighborhood context and mortality in late life. *J Aging Health, 22*(2), 197-218. doi:10.1177/0898264309355980

Wight, R. G., Cummings, J. R., Miller-Martinez, D., Karlamangla, A. S., Seeman, T. E., & Aneshensel, C. S. (2008). A multilevel analysis of urban neighborhood socioeconomic disadvantage and health in late life. *Soc Sci Med, 66*(4), 862-872. doi:10.1016/j.socscimed.2007.11.002

Wörn, J., Ellwardt, L., Aartsen, M., & Huisman, M. (2017). Cognitive functioning among Dutch older adults: Do neighborhood socioeconomic status and urbanity matter? *Soc Sci Med, 187*, 29-38. doi:10.1016/j.socscimed.2017.05.052

Wu, Y. T., Prina, A. M., Jones, A. P., Barnes, L. E., Matthews, F. E., & Brayne, C. (2015). Community environment, cognitive impairment and dementia in later life: results from the Cognitive Function and Ageing Study. *Age Ageing, 44*(6), 1005-1011. doi:10.1093/ageing/afv137

Yan, T., Escarce, J. J., Liang, L. J., Longstreth, W. T., Jr., Merkin, S. S., Ovbiagele, B., . . . Brown, A. F. (2013). Exploring psychosocial pathways between neighbourhood characteristics and stroke in older adults: the cardiovascular health study. *Age Ageing, 42*(3), 391-397. doi:10.1093/ageing/afs179

Yao, L., & Robert, S. A. (2008). The contributions of race, individual socioeconomic status, and Neighborhood socioeconomic context on the self-rated health trajectories and mortality of older adults. *Res Aging, 30*(2), 251-273. doi:10.1177/0164027507311155

Yao, L., & Robert, S. A. (2011). Examining the Racial Crossover in Mortality between African American and White Older Adults: A Multilevel Survival Analysis of Race, Individual Socioeconomic Status, and Neighborhood Socioeconomic Context. *J Aging Res, 2011*, 132073. doi:10.4061/2011/132073
